# Automation of gene assignments to metabolic pathways using high-throughput expression data

**DOI:** 10.1186/1471-2105-6-217

**Published:** 2005-08-31

**Authors:** Liviu Popescu, Golan Yona

**Affiliations:** 1 Department of Computer Science, Cornell University, Ithaca, NY

## Abstract

**Background:**

Accurate assignment of genes to pathways is essential in order to understand the functional role of genes and to map the existing pathways in a given genome. Existing algorithms predict pathways by extrapolating experimental data in one organism to other organisms for which this data is not available. However, current systems classify all genes that belong to a specific EC family to all the pathways that contain the corresponding enzymatic reaction, and thus introduce ambiguity.

**Results:**

Here we describe an algorithm for assignment of genes to cellular pathways that addresses this problem by selectively assigning **specific **genes to pathways. Our algorithm uses the set of experimentally elucidated metabolic pathways from MetaCyc, together with statistical models of enzyme families and expression data to assign genes to enzyme families and pathways by optimizing correlated co-expression, while minimizing conflicts due to shared assignments among pathways. Our algorithm also identifies alternative ("backup") genes and addresses the multi-domain nature of proteins.

We apply our model to assign genes to pathways in the Yeast genome and compare the results for genes that were assigned experimentally. Our assignments are consistent with the experimentally verified assignments and reflect characteristic properties of cellular pathways.

**Conclusion:**

We present an algorithm for automatic assignment of genes to metabolic pathways. The algorithm utilizes expression data and reduces the ambiguity that characterizes assignments that are based only on EC numbers.

## Background

Pathways are cellular procedures that are associated with a specific functionality in the cell, such as amino acid synthesis and degradation, energy metabolism, signal transduction, molecular oxidation, and more. The complexity of a cell is a function of its underlying procedures. Therefore, there is a strong interest in identifying the active pathways in an organism. This knowledge can not only shed light on the mechanisms the cell uses to acquire its functional role; by assigning genes to pathways one can also better understand the exact role of these genes, and identify key genes whose existence is crucial to sustain normal cell functionality.

A wealth of experimental data about molecular complexes and cellular processes that has been accumulated in the literature initiated several projects that attempted to compile the existing knowledge into publicly available databases. Among these are EMP [1], MPW [2], WIT [3], UM-BBD [4], KEGG [5], MetaCyc [6], ERGO [7] and SEED [8]. These databases store valuable information about hundreds of pathways and cellular processes.

Much of the research on pathways so far focused on extrapolating pathways from one organism to other. The goal of this research goes beyond just storing, analyzing and extrapolating the biochemical information and strives to improve the known data by discovering variations to pathways in different organisms as well as to discover novel pathways.

Attempting to complement the experimental data and extend its utility to other systems and newly sequenced genomes, several methods were developed for pathway prediction. One approach to pathway reconstruction is to utilize the existing knowledge on enzymatic reactions to create a complete graph of a possible metabolic network [9-12]. However, this approach is sought with complexity problems and it is hard to verify the validity of these predictions. Several studies manually constructed and curated the metabolic networks for organisms like Escherichia coli [13-15], Haemophilus influenzae [16] and Saccharomyces cerevisiae [17,18], from a variety of data sources and literature. These studies have an advantage over automated pathway reconstruction, as the reconstructed networks are more likely to be biologically plausible. However, this approach requires close human intervention.

Perhaps the most popular approach for pathway prediction is based on extrapolation. Procedures developed by WIT, KEGG, MetaCyc and ERGO use blueprints of pathways collected either from biochemical charts or from actually observed pathways in different organisms, and assign genes to pathways based on homology between genes across organisms, database annotations, and manual curration. Specifically, many known pathways are metabolic pathways that consist mostly of sets of reactions catalyzed by specific enzymes that are designated by their Enzyme Classification (EC) number [19]. Most of the existing methods for metabolic pathway prediction that are based on pathway blueprints assign the vast majority of genes to pathways based on their EC designation (some address also the problem of finding missing enzymes [20-24]). However, since certain reactions appear in multiple pathways, this method will assign all enzymes that can catalyze a certain reaction (termed *isozymes*) to all pathways that contain this reaction. For example, genes that are designated as malate dehydrogenase (EC 1.1.1.37) are classified to several different pathways (including mixed acid fermentation, gluconeogenesis, superpathway of fatty acid oxidation and glyoxylate cycle, respiration, and more), all of which use the same oxidization reaction that is catalyzed by these genes.

Clearly, this nondiscriminatory assignment of genes to pathways is suboptimal, as it is unlikely that *all *genes with the same EC designation are used in *all *pathways that contains the corresponding reaction. Rather, it is more likely that different genes are used in different pathways, and it has been suggested [25] that "the primary role of isozymes is to allow for differential regulation of the reactions that are shared by different processes". However, this information is sparse and without additional experiments it is very hard to make this type of functional differentiation. The extent of this problem is not negligible. For example, of the 469 pathways in MetaCyc, 336 have at least one reaction in common with another pathway. Since in most genomes there are multiple instances of some enzyme families, the common method for pathway prediction (that is based only on EC numbers) results in *many-to-many *ambiguous mapping between genes and pathways.

Pathway assignment can be aided by the existence of microarray technology [26-29]. This technology enables genome-wide measurements of cell activity, providing us with snapshots of the molecular machinery at different times along the cell cycle and under different experimental conditions. This data can help to identify groups of genes that are co-expressed, i.e. that are likely to exist in the cell at the same time or under the same set of conditions. Although the sequence of reactions in a pathway does not take place simultaneously, given the time-resolution of the mRNA expression measurements these reactions can be considered to occur instantly and simultaneously for all practical purposes. Therefore, it is expected that genes that participate in the same pathway will have similar expression profiles, i.e. they will co-exist and will be concurrently available at the cell's disposal to complete the pathway. Indeed, correlation in expression profiles has been observed for *linear pathways *that consist of sequences of reactions [25]. It has been also shown that prediction based on search in the pathway space improves when pathways are scored using expression data [30]. Other studies used expression data to score gene classes and pathways, in search of interesting classes or modules of genes or to verify the existence of certain pathways in a genome [31-38]. Expression data can also suggest the existence of control mechanisms and pathway switches. For example, when a pathway has a fork, isozymes might be used to switch between the alternate routes, resulting in anti-correlation [25]. A detailed discussion of these studies and others in the field of pathway prediction and analysis appears in Appendix A.

Here, based on this premise, we propose a method for improving the gene-to-pathway assignment problem and refining the large-scale predictions of pathways provided by systems like WIT, Pathway Tools and KEGG (that use EC designation only). Our method utilizes pathway blueprints, statistical models of protein families and expression data. As opposed to previous methods, our algorithm focuses on elucidating the correct assignment of genes to pathways and expression data is used to score *assignments *rather than pathways. Our algorithm predicts all assignments simultaneously, while resolving possible conflicts and optimizing the correlated expression.

The paper is organized as follows. We first describe our model and the prediction algorithm. Next we evaluate our methods by running a full-scale prediction on the yeast genome. Finally we compare our predictions to the few assignments that were verified experimentally.

## Results

Our model organism is Yeast. This choice was motivated by the myriad of experimental data available for the Yeast genome, and specifically, time-series expression data which is not readily available for other genomes. Our study integrates pathway data with expression data and sequence data. Information on the datasets used in this study is available in the 'Methods' section.

There are many definitions of pathways in the literature and on-line, depending on the context in which they are used. In our work we adopt the same definition that is used in many other studies and underlies the pathways in databases such as MetaCyc and KEGG. As was characterized concisely in [39]: "A metabolic pathway is a sequence of consecutive enzymatic reactions that brings about the synthesis, breakdown, or transformation of a metabolite from a key intermediate to some terminal compound. A metabolic pathway may be linear, cyclic, branched, tiered, directly reversible, or indirectly reversible."

We formalize the concept of a metabolic pathway according to this definition and it is assumed that each pathway *P *consists of a set of enzymatic reactions which together perform a certain function. Each reaction can be catalyzed by enzymes that are typically associated with one Enzyme family *F*.

### Pathway assignments – algorithm overview

Our algorithm for assigning genes to pathways takes as input

• A genome *G *= {*g*_1_, *g*_2_, ..., *g*_*N*_}

• Expression data **E **= {**E_i_**} where **E_i _**is the expression profile of gene *g*_*i*_

• An assignment of genes to enzyme families **F **= {*F*_1_, .., *F*_*J*_}

• A set of metabolic pathways **P **= {*P*_1_, .., *P*_*K*_}.

Our method consists of the following steps:

1. Search for probable pathways. For each pathway *P*_*k *_∈ **P**:

(a) match enzymes with the reactions that make up the pathway;

(b) eliminate the pathway if more than *θ *of the reactions cannot be associated with genes (here we set *θ *= 0.5).

The resulting set of pathways is denoted **P'**

2. Compute initial pathway assignments and sort assignments according to the score from high to low.

3. Refine assignments. Given the assignments from the previous step:

(a) compute the conflict graph;

(b) compute the connected components in the conflict graph;

(c) solve the conflicts within each connected component.

We now proceed to describe each step in detail.

### Search for probable pathways

To assign genes to pathways in a given sequenced and annotated genome, we use the descriptions of the pathways from MetaCyc and the classification of genes to enzyme families (based on annotations or statistical models, as described in 'Methods') to initially match each enzyme with a reaction and therefore with a pathway.

We denote by **F**(*P*_*k*_) = {*F*_1_, *F*_2_, ..., *F*_*m*_} the set of **pathway families **– the protein families that catalyze the reactions that make up pathway *P*_*k*_, where *m *is the number of different reactions (the number of reactions need not be equal to the number of families, however, each reaction in a pathway is usually associated with one family). A pathway is kept if at least *m*/2 of its reactions can be assigned with enzymes. Formally, denote by **F**(*G*) the set of families that can be associated with at least one gene in the genome *G*. A pathway *P*_*k *_is considered *probable *in the genome *G *if |**F**(*P*_*k*_) ∩ **F**(*G*)| ≥ *m*/2. We denote the set of probable pathways by **P'**, and our algorithm proceeds only with pathways in **P'**. Note that at this stage there might be multiple genes assigned to the same reaction.

### Initial pathway assignments

After eliminating the improbable pathways we generate initial assignments by computing the best individual assignment for each candidate pathway, independently. We are given a pathway *P*_*k *_with *m *reactions. In search for the optimal assignment we consider all genes in each one of the families *F*_1_, *F*_2_, ..., *F*_*m *_∈ **F**(*P*_*k*_), resulting in |*F*_1_| × |*F*_2_| × ...|*F*_*m*_| possible assignments. Each possible combination is considered and we evaluate its significance by computing the total correlation score between genes. I.e. the score of assignment **A **= (*g*_1_, *g*_2_, ..., *g*_*m*_) s.t. *g*_*i *_∈ *F*_*i *_is defined as the average co-expression score



where *sim*(**E**_*i*_, **E**_*j*_) is the expression similarity of genes *g*_*i *_and *g*_*j *_as described in 'Methods' and *w*_*i *_is the weight that represents the likelihood that gene *g*_*i *_belongs to family *F*_*i *_and is defined as  where *evalue*(*i*) is the significance of the match between gene *i *and the statistical model of family *F*_*i *_(see 'Methods'). For example, assume the best match with family *F*_*i *_is observed for an annotated gene with evalue of 10^-20^. Then a gene that is classified to that family with evalue of 10^-10 ^is assigned a weight of 0.5.

After computing all the assignment scores we sort them in the order from best to worst. The best assignment is selected as the one that maximizes the average score.

#### Multi-domain proteins

Of the 71,638 proteins in our database with an EC designation (see section 'Data sets' in 'Methods'), about 1241 have multiple enzymatic domains. Of which, the majority (1076 proteins) are two-domain proteins that form 173 unique combinations. A simple statistical analysis reveals that these proteins are more likely to contribute all their domains to the same pathway. Specifically, we computed the fraction of two-domain enzymes that can be completely mapped to a single pathway (i.e. there exist at least one pathway such that all the enzymatic domains take part in). Of the 173 two-domain combinations, 67 are combinations of domains that are in our pathway data set. Of which 48 (72%) can be mapped completely to a single pathway. The *expected *fraction is estimated assuming that the two domains are chosen at random from the domain library, and computing how many random pairs appear in the same pathway. Of the 6786 possible combinations of domain pairs (using the domain library derived from the set of 67 combinations used above) only 199 (3%) are mapped to a single pathway. The significant difference (72% vs. 3%) indicates a clear bias for multi-domain proteins.

This is not surprising, as multi-functional proteins would be thermodynamically favorable in pathways. If two reactions in a pathway can be catalyzed by the same protein, the efficiency of the reaction can significantly increase, since it saves the need to localize and control the expression of multiple proteins. If the two reactions are consecutive, it is quite likely that the output of one reaction is immediately transferred as an input to the second reaction catalyzed by the second domain. To account for this scenario in our model and create a natural bias toward multi-domain proteins, we use the self-similarity score when assigning these genes to two (or more) different reactions within the same pathway. With that bias, multi-domain proteins will be preferred whenever some or all their domains can be utilized in the same pathway.

#### Computational issues

To find the best assignment of genes to a given pathway we exhaustively enumerate all possible assignments. This is possible for most pathways, genomes and families. For example, most of the pathways in Yeast have less than a hundred possible assignments in our current setting. However, some of the protein families are fairly large (hundreds and even thousands of members), resulting in a large number of possible assignments. The maximum number of pathway assignments in Yeast is observed for the tRNA charging pathway which has 49,152 possible assignments. Considering all possible combinations in the cross-product is computationally intensive, and also unnecessary. To reduce the number of assignments that are considered one can first compute the similarity scores of all possible pairs and use only pairs that have significant similarity score (see the 'Metrics' section in 'Methods') or limit the analysis to the top N scoring pairs. In practice, given the size of a typical pathway as well as the number of possible genes catalyzing a reaction, the complete enumeration of assignments is possible in a reasonable time (a matter of minutes).

### Refining the assignments

The initial set of assignments is likely to produce a good unique mapping between genes and pathways (see 'Discussion'). However, since each pathway is analyzed independently it might happen that the same gene is assigned to the same reaction in multiple pathways. Each such assignment is considered a **conflict**. Although in some cases the same gene might play the same functional role in different pathways, our hypothesis is that if there are multiple enzymes in the same genome that can catalyze the same reaction, and that reaction takes place in multiple pathways, then it is more probable that each enzyme is "specialized" to catalyze this reaction in a different pathway. To eliminate the conflicts we revisit the assignments and resolve them whenever it is possible, as described next.

#### The pathway conflict graph

**Figure 1:**
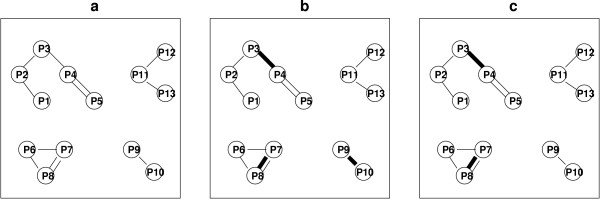
**Pathway graphs**. Left: the pathway relation graph. Each pathway is represented as a node, and an edge is drawn between two pathways for each reaction that they share in common. Middle: the pathway conflict graph. Thick edges represent conflicts (i.e. the same gene was assigned to catalyze the same reaction in both pathways connected by the edge). Right: the final conflict graph. The edge between pathways *P*9 and *P*10 is a flat edge (no alternative assignments exist for that reaction) and therefore it is unmarked. At the end we are left with only two connected components with possibly solvable conflicts.

We start by constructing the *pathway relation graph*. In this graph each pathway is a node, and two nodes are connected by an edge if the two pathways represented by the nodes share a reaction (see Figure 1a). We introduce one edge for each such reaction (i.e. there might be multiple edges connecting the same two nodes). The **pathway conflict graph **is derived from this graph: we mark an edge as a **conflict **if the corresponding reaction is associated with the same gene in both pathways, based on the initial assignments (see Figure 1b).

#### Connected components

The pathway conflict-graph can be split into connected components, each of which is composed of several pathways connected by edges (reactions), some of which are marked and indicate possible conflicts. If several genes are associated with such a reaction, it might be possible to resolve this conflict. Clearly, the assignments in one connected component have no effect on the other connected components and therefore we can revisit these assignments independently for each connected component.

It should be noted that not all conflicts can be resolved. If in the given genome there is only a single gene that can be associated with a specific reaction, then clearly it is not possible to refine conflicts associated with that reaction. An edge that is linked with such a reaction is referred to as a **flat **edge. Since for flat edges no alternative assignments exist (given the gene data), we unmark these edges in the conflict graph. Our algorithm operates *only on connected components with marked edges *(see Figure 1c).

#### Assignment of genes to pathways in a connected component

To find the best non-conflicting assignment of genes to pathways in a connected component we generalize our scoring function such that the score of an assignment is the sum of the scores of the assignments to pathways contained in the component, with the restriction that no enzyme can be used twice to catalyze the same reaction in different pathways. We ignore inter-pathway expression data correlations, assuming different pathways are associated with different cellular processes and therefore are not expected to be correlated.

Formally, given a set of pathways **P **= *P*_1_, *P*_2_, .., *P*_*k *_and an assignment **A**, the assignment score is simply the total weighted co-expression score



where **A**(*P*_*i*_) is the subset of genes assigned to pathway *P*_*i *_and *Score*(**A**(*P*_*i*_)) is as defined previously. As before we enumerate all possible assignments of genes to pathway families and each assignment is also marked with the number of conflicts it introduces.

Ideally we would like to find high scoring assignments that are conflict-free. However, this ideal situation is not always attainable as some shared reactions are central and are best catalyzed by the same enzyme. For example, reaction 2.6.1.1 that is shared by the asparagine and aspartate biosynthesis pathways is catalyzed in both pathways by gene AAT2, although there exist another gene (AAT1) that can catalyze this reaction (see 'Discussion'). Moreover, some pathways are superpathways of other pathways and are naturally composed of the same genes. Therefore, not all conflicts can and should be resolved. To accommodate these possible scenarios we consider all assignments that are within Δ from the maximal score that is obtained when conflicts are allowed, and pick the one that has the minimal number of conflicts within that range (without a better methodology at this point, the exact value of Δ is currently set ad-hoc to 1). A significant drop in the score of a conflict-free assignment (compared to the highest scoring assignment) suggests that some reactions are indeed catalyzed by the same gene, despite the fact that alternative genes do exist to perform similar functions.

## Discussion

Evaluating our pathway prediction algorithm requires the availability of well studied and annotated genome for which high-quality expression data and empirical knowledge of pathways exist. Since most pathway databases assign genes to pathways collectively based on the EC designation it was hard to find an extensive set of literature-curated pathways. We used one of the curated PGDB Yeast Biochemical Pathways [44] at the Saccharomyces Genome Database (SGD) [45]. This database was computationally derived from the Yeast sequenced and annotated genome using the Pathway Tools software [46] and the pathway blueprints from the Metacyc database [6], and was then manually curated by mining the literature. Not all the assignments were done based on direct phenotype experiments and the confidence in the assignments varies depends on the type of the evidence used. The database contains 58 pathways, many of which did not exist in the Metacyc database or did not match perfectly with the pathway blueprints in Metacyc. A few other pathways contained genes that we were not able to map to our Yeast protein database, and were eliminated as well. This left us with 25 pathways that were used for testing. Each curated pathway in the SGD database describes a sequence of reactions as well as the genes that catalyze the reactions. Some reactions are not associated with a specific gene and were not considered when evaluating the correctness of an assignment. Also, some of the reactions are unclassified reactions that either have an incomplete EC number or do not have an EC number at all. These reactions are currently ignored in our experiments.

It should be noted that some of the curated pathways associate multiple genes with the same reaction. In general, it seems that there are two possible explanations. It might be the case that a complex of proteins catalyzes the reaction and the genes associated with the reaction are part of this complex. In this case we want to assign all proteins to the reaction. This is not taken into account in our algorithm currently. The other more common case is when each protein can catalyze the reaction by itself, for example under different specific cellular conditions. This can be verified in knockout experiments and has been observed in several systems (e.g. [47]). While it is possible that all these genes are used concurrently, our assumption is that only a few of them actually do. In these cases, our algorithm can assess the "affinity" of each gene with the pathway. In the next sections we discuss our results and compare them with the curated assignments of the pathways in the test set.

### Pathway assignment for curated pathways

To test our predictions, we run the algorithm on the Yeast genome, using the time-series expression data and the blueprints of the 25 pathways in our test set. The EC annotations were updated to be consistent with those used by SGD. It should be noted that for many pathways *all *possible assignments are curated as valid assignments by SGD. A summary of the results is given in Table 1.

**Table 1:** Summary of pathway assignments. For each pathway in the test set we report the number of reactions, the number of assignments considered, the number of curated (SGD verified) assignments, and the maximal and minimal assignment scores. The score reported is the weighted average score per pair of compared enzymes. The score reflects the average significance of a pairwise relation within a pathway. The larger the score the more significant is the relation. Negative scores suggest anti-correlation and near-zero scores provide no evidence that the two genes are related. Pathways are sorted based on assignment score.

Pathway	Number of reactions	Number of assignments	Number of curated assignments	Max(Min) Score Normalized
methionine and *S*-adenosylmethionine synthesis	2	2	2	10.45 (7.34)
isoleucine biosynthesis I	5	12	4	10.32 (3.00)
leucine biosynthesis	4	4	4	10.08 (4.01)
valine biosynthesis	4	4	4	10.14 (4.99)
asparagine biosynthesis I	2	4	4	8.80 (-4.88)
proline biosynthesis I	3	1	1	8.43 (8.43)
homoserine methionine biosynthesis	2	1	1	7.33 (7.33)
tryptophan biosynthesis	5	2	2	5.29 (4.13)
aspartate biosynthesis II	2	4	4	4.85 (0.75)
non-oxidative branch of the pentose phosphate pathway	5	8	8	4.82 (0.84)
folic acid biosynthesis	11	48	32	4.63 (1.26)
glutamate biosynthesis I	2	2	2	4.08 (-4.88)
glutathione biosynthesis	2	1	1	4.03 (4.03)
glutamate degradation VIII	5	1	1	3.92 (3.92)
serine biosynthesis	3	2	2	3.58 (-0.58)
purine biosynthesis 2	14	16	8	2.50 (2.06)
homocysteine and cysteine interconversion	3	2	1	2.35 (2.01)
biotin biosynthesis I	3	1	1	2.27 (2.27)
homocysteine degradation I	2	1	1	2.01 (2.01)
threonine biosynthesis from homoserine	2	1	1	0.87 (0.87)
glutamine – glutamate pathway II	1	1	1	0.00 (0.00)
tyrosine biosynthesis I	3	2	2	-0.53 (-0.58)
glycine biosynthesis I	2	2	2	-0.91 (-3.60)
phenylalanine biosynthesis I	3	2	2	-2.09 (-2.80)

Almost all curated assignments are assigned high positive scores (results not shown). There are some exceptions and a few curated assignments have a negative score. In these cases most or all other assignments have negative scores as well. For 13 out of the 25 pathways the maximum normalized score is greater than 4. The score is an indication of how significant is the similarity of two expression profiles [43]. An average score greater than 4 means that the enzymes assigned to the pathways are similarly expressed with high confidence and are likely to be functionally linked. Moreover, for 10 out of the 25 pathways, all pairwise scores (for all pairwise relations) in the top-scoring assignment are positive. These results support our assumption that proteins that participate in the same cellular process are similarly expressed. It is also observed that curated assignments are assigned better scores than the non curated assignments and the best assignment is usually a curated assignment. In the next subsections we take a closer look at some interesting pathways.

### The isoleucine biosynthesis pathway

**Figure 2:**
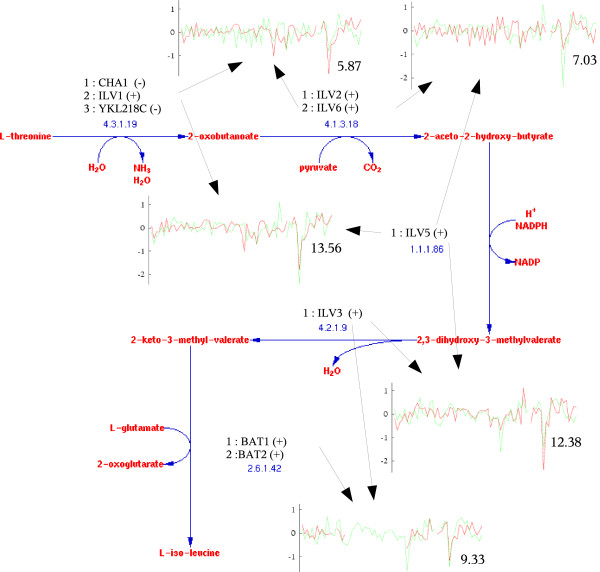
**The Isoleucine Biosynthesis pathway diagram**. The pathway layout is retrieved from the MetaCyc database. For each reaction we list the genes that can catalyze the reaction. A plus or minus sign indicates if the gene was assigned to the pathway in SGD. The expression profiles and their similarity score are shown for selected pairs of genes. Mapping between gene names and Biozon identifiers is given in Table 6.

As Table 1 indicates, the expression data strongly supports the existing knowledge about pathways and can be used for prediction. The isoleucine biosynthesis pathway is one such example (Figure 2). This pathway consists of 5 reactions. A total of 12 assignments are considered, of which 4 are curated and are considered true assignments, and 8 are considered false assignments. Table 2 lists detailed information about each candidate assignment. Note that curated assignments are assigned a high positive score, and the normalized score of the best assignment is well over 4. Moreover, the true and false assignments are well separated in the sorted list. The baseline score is determined by the two enzymes (EC 1.1.1.86 and EC 4.2.1.9) that have no alternative genes and are shared by all assignments. Looking at the break-up of pairwise similarities within the pathway we note that almost all of them have positive scores for curated assignments, while false assignments contain more pairs with negative pairwise scores.

**Table 2:** Assignments for the pathway isoleucine biosynthesis I. Only reactions with alternative assignments are listed (last column), and the selection number refers to Figure 2. For example, the top assignment selects the second gene (ILV1) to catalyze reaction 4.3.1.19. Assignments are sorted based on the normalized score. Second column marks which assignments are true assignments, and which are considered false assignments. For each assignment we list the total number of pairwise similarities, the number of positive and negative scoring pairs and the number of zero scoring pairs (when no expression data is available).

Number	Match	Normalized Score	Number of Pairs	Positive Pairs	Negative Pairs	Zero Pairs	Assignments
1	+	10.32	10	10	0	0	4.3.1.19 : 2 4.1.3.18 : 2 2.6.1.42 : 1
2	+	9.54	10	10	0	0	4.3.1.19 : 2 4.1.3.18 : 1 2.6.1.42 : 1
3	+	7.37	10	10	0	0	4.3.1.19 : 2 4.1.3.18 : 2 2.6.1.42 : 2
4	-	6.49	10	8	2	0	4.3.1.19 : 3 4.1.3.18 : 2 2.6.1.42 : 1
5	+	6.30	10	9	1	0	4.3.1.19 : 2 4.1.3.18 : 1 2.6.1.42 : 2
6	-	6.08	10	6	0	4	4.3.1.19 : 1 4.1.3.18 : 2 2.6.1.42 : 1
7	-	5.52	10	6	0	4	4.3.1.19 : 1 4.1.3.18 : 1 2.6.1.42 : 1
8	-	5.00	10	7	3	0	4.3.1.19 : 3 4.1.3.18 : 1 2.6.1.42 : 1
9	-	4.78	10	8	2	0	4.3.1.19 : 3 4.1.3.18 : 2 2.6.1.42 : 2
10	-	3.84	10	6	0	4	4.3.1.19 : 1 4.1.3.18 : 2 2.6.1.42 : 2
11	-	3.00	10	6	4	0	4.3.1.19 : 3 4.1.3.18 : 1 2.6.1.42 : 2
12	-	3.00	10	5	1	4	4.3.1.19 : 1 4.1.3.18 : 1 2.6.1.42 : 2

Note that the first two assignments are both assigned high positive scores. These two assignments differ in the gene used to catalyze the EC reaction 4.1.3.18. The first is using ILV2 while the second is using ILV6 Interestingly, these proteins form a complex which catalyzes the reaction 4.1.3.18 [45]. This and similar cases will be handled in future versions of our algorithm (see the Conclusion section). Of the curated assignments, the fourth one leads to one negative pairwise score of -3.04 for proteins ILV6 and BAT2. Protein BAT2 can catalyze the reaction 2.6.1.42. This reaction can also be catalyzed by protein BAT1, and its selection results in better assignment scores. The two proteins are very similar in sequence (77% identity), however, the former is highly expressed during stationary phase of the cell-cycle and down-regulated during the logarithmic phase of growth (as is documented in the SwissProt record of that gene), while the later exhibits the opposite behavior. In view of the expression data it is unlikely that the two genes participate in this pathway at the same time, and gene BAT2 is probably assigned only during the stationary phase where the pathway activity is reduced.

We compared our results with the reconstructed metabolic network of Saccharomyces cerevisiae, as described in [18] (see 'Related Studies'). This network is not compartmentalized into separate metabolic pathways, however, the reactions are grouped according to the cellular process they are involved with. The comparison revealed discrepancies between the pathway data from Metacyc and SGD and the metabolic network model, which complicated the comparison of the results.

For example, the "isoleucine biosynthesis I" MetaCyc pathway overlaps with the reaction group "Valine, leucine, and isoleucine metabolism". The group has 24 reactions while the MetaCyc pathway has five, of which four are part of the group and the fifth (reaction 4.3.1.19 which appears first) is part of the "Threonine and Lysine Metabolism" group. Moreover, while the EC numbers and the sequence of reactions with respect to the EC numbers are the same in MetaCyc and the network model, the reactions are different because they do not use the same substrates as intermediary metabolites.

**Figure 3:**
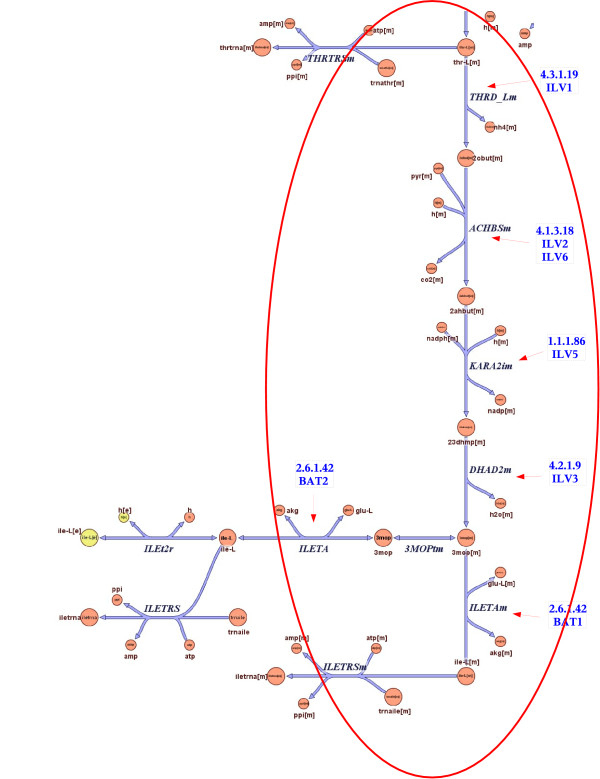
**The Isoleucine Biosynthesis pathway from the reconstructed metabolic network of Saccharomyces Cerevisiae [18]**. Reproduced with permission from Cold Spring Harbor Laboratory ^©^2004 (Duarte et al. 2004 [18]). The EC numbers and the genes associated with the reactions were added to diagram. The part that overlaps with the MetaCyc isoleucine biosynthesis pathway is circled.

Interestingly, the first four reactions in the isoleucine biosynthesis MetaCyc pathway take place inside the mitochondrion, while the last step of the pathway, reaction 2.6.1.42 (catalyzed by BAT1 and BAT2), takes place both in the mitochondrion and in the cytoplasm. Indeed, it has been verified experimentally that BAT1 resides in the mitochondrion while BAT2 resides in the cytoplasm (see Figure 3). In order to obtain cytoplasmic isoleucine, a transport reaction is necessary to transfer the final intermediary metabolite. That might explain why BAT2 is slightly uncorrelated with the other genes in the pathway. Such situations lead to "forks", where two branches are uncoupled even if they have the same EC number. Our assignments are consistent with these observations.

### The folic acid biosynthesis

The metabolic network along with other cellular processes form the computational elements of the cell and as with any computation of this magnitude it needs to be regulated and synchronized. This regulation is reflected in the expression levels of genes. A possible synchronization device might require for example that reaction A is not started until reaction B is completed. Therefore, the enzymes that can catalyze A and B are not expected to be active at the same time, a state that can be achieved by controlling the expression levels of the corresponding genes. This type of mechanism will create a functional anti-correlation which will be awarded with a negative score by our scoring system. Beyond controlling timing of reactions, anti-correlation might also reflect a control mechanism that is used to govern pathway activity and metabolic rate.

**Figure 4:**
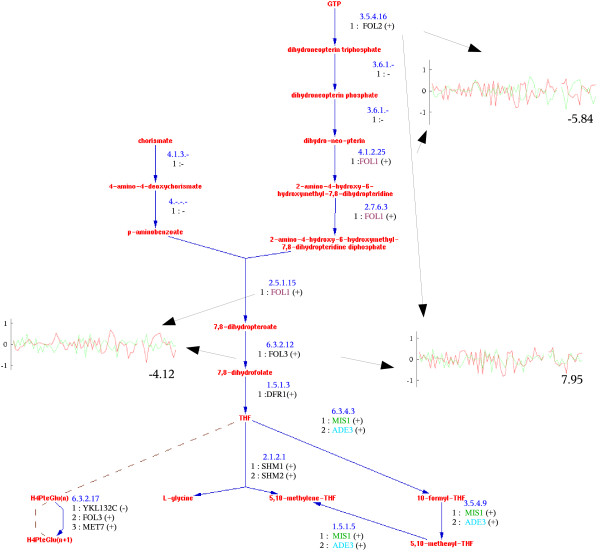
**The folic acid biosynthesis pathway diagram**. See Figure 2 for description. Note that FOL1, ADE3 and MIS1 are multi-functional enzymes.

An illustration of this mechanism is the pathway *folic acid biosynthesis *whose trajectory traverses both the mitochondrion and the cytoplasm. This is a quite complex pathway (see Figure 4) and the mechanism might occur between genes FOL1, FOL2 and FOL3. FOL1 is present in the mitochondrion while FOL2 and FOL3 are in the cytoplasm. Gene FOL1 is strongly anti-correlated with genes FOL2 and FOL3 while these two genes are strongly correlated between them. Note that the input to the last reaction catalyzed by FOL1 is the output from two different parallel branches of the pathway (top part). The anti-correlation might serve as a synchronization mechanism to control the reactants flow in the pathway in the presence of forks (that split into or merge different branches).

Interestingly, gene FOL1 is an enzyme with multiple enzymatic functions positioned between the reactions catalyzed by FOL2 and FOL3. FOL1 has three different enzymatic domains, classified as 4.1.2.25, 2.7.6.3 and 2.5.1.15. There are no other genes that are classified (based on database annotations or sequence similarity) to either of these three enzyme classes. This further supports our assumption that multi-domain enzymes are more likely to catalyze several reactions in the same pathway, are are preferred over different enzymes, each assigned to a different reaction.

Multi-domain enzymes are also used in the lower part of the pathway. Both MIS1 and ADE3 catalyze three different consecutive reactions. Both are assigned to this pathway by SGD, however, surprisingly, their mutual expression similarity is negative (-2.09), indicating anti-correlation. Interestingly, the three reactions are shared with other pathways (glycine degradation, formylTHF biosynthesis and carbon monoxide dehydrogenase pathway), and it is hypothesized that the two isozymes, MIS1 and ADE3, serve as switches, to control the pathway activity and its coupling with other pathways. Indeed, such a mechanism has been suggested in [25] to control pathway flow.

**Figure 5:**
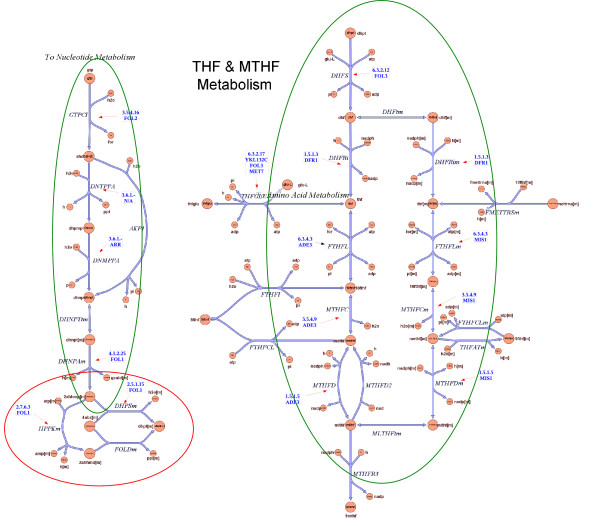
**The folic acid biosynthesis pathway from the reconstructed metabolic network of Saccharomyces Cerevisiae [18]**. Reproduced with permission from Cold Spring Harbor Laboratory ^©^2004 (Duarte et al. 2004 [18]). The EC numbers and the genes associated with the reactions were added to diagram. The parts that overlap with the MetaCyc folic acid biosynthesis pathway are circled. The green circles indicate consistency while the red one indicates inconsistency.

To better understand these mechanisms we compared our results with the metabolic network model of [18]. The pathway from MetaCyc has 15 reactions, of which 12 overlap with the reaction group "Folate Metabolism", which has 29 reactions. Four out of these 12 reactions are duplicated in the metabolic network model with one instance in the cytoplasm and one in the mitochondrion. The discrepancy between MetaCyc and the network model involves the sequence of reactions 4.1.2.25, 2.7.6.3 and 2.5.1.15, all catalyzed by FOL1 gene, which are differently connected in the network model (see Figure 5). The network model also shows a fourth catalytic function for FOL1. The location of the enzymes and reactions in the network model indicate the intricate trajectory between cytoplasm and mitochondrion.

In view of these discrepancies, choosing the "right" model for this pathway is difficult. However, our results confirm the interplay between cytoplasm and mitochondrion and can help distinguish between mitochondrial and cytoplasmic genes, as each subgroup is mutually co-expressed, suggesting that the pathway expression is controlled by two distinct regulatory programs.

### The asparagine biosynthesis pathway

Not always it is possible to explain negative pathway scores (anti-correlation or no correlation). Sometimes, a gene that can catalyze a specific reaction in a pathway is not coordinated with the other genes in the pathway. This might be due to the fact that the gene functions in the pathway only under certain conditions while inactive under others [27]. Or the gene might serve as a backup gene that is activated only when the main one is missing or is malfunctioning [47]. This problem is especially pronounced if the main gene has not been identified yet. Indeed, despite extensive annotation efforts, many genes have not been characterized yet.

**Figure 6:**
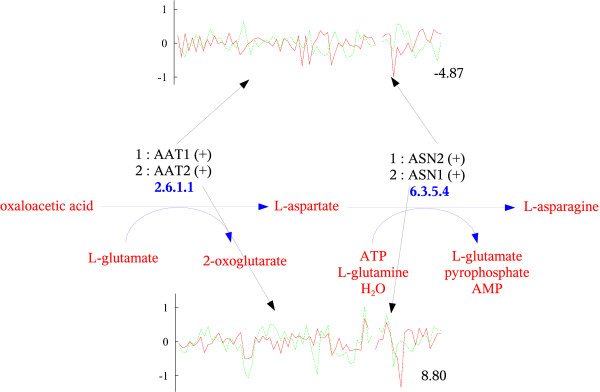
**The asparagine biosynthesis pathway**. See Figure 2 for description. Both ASN1 and ASN2 are correlated with AAT2 but are anti-correlated with AAT1 (selected pairwise similarities are shown). The later is localized to a different cellular compartment than the others, and is likely to be involved in other pathways (see text for details).

**Figure 7:**
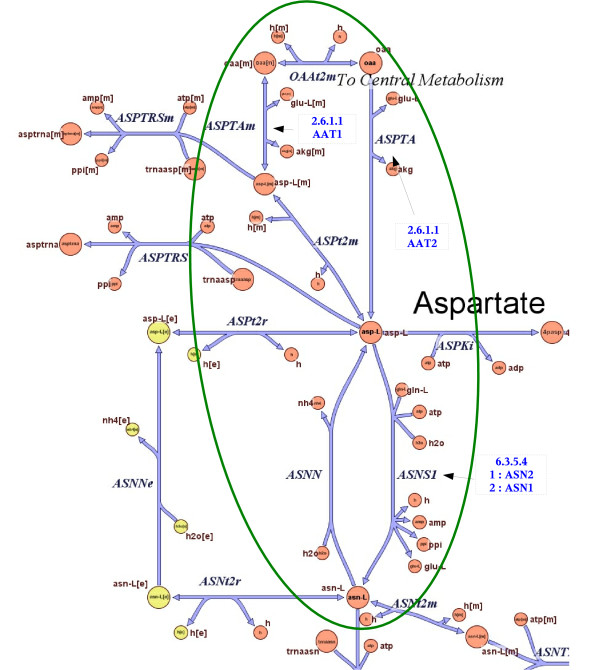
**The asparagine biosynthesis pathway from the reconstructed metabolic network of Saccharomyces Cerevisiae 18**. Reproduced with permission from Cold Spring Harbor Laboratory ^©^2004 (Duarte et al. 2004 [18]). The part that overlaps with the MetaCyc asparagine biosynthesis pathway is circled.

By analyzing pairwise scores within a pathway, our method can suggest which genes fit together better in the context of the pathway and which genes are unlikely to work together. Moreover, if the overall assignment score is negative then it might be the case that the pathway is not active in the expression data collected or the pathway might not exist in the organism at all. Negative scores might also expose errors in pathway assignments. One interesting example is the asparagine biosynthesis pathway (Figure 6). This pathway is intriguing, having four curated assignments, two of them with negative scores. This is a small pathway with only two reactions. It is gene AAT1, which catalyzes the first reaction of the pathway (2.6.1.1), that is responsible for the negative scores of two assignments. This gene is strongly anti-correlated with genes ASN1 (-2.24) and ASN2 (-4.87), which catalyze the second reaction. On the contrary, gene AAT2 is strongly correlated with both genes ASN1 (8.80) and ASN2 (8.56). Interestingly, the reaction 2.6.1.1 is shared with other three pathways (asparagine degradation, aspartate biosynthesis and glutamate degradation VII). Our results suggest that the two isozymes, which can catalyze the same reaction, are used selectively in different pathways; AAT2 is involved in asparagine and aspartate biosynthesis, while AAT1 is involved in asparagine and glutamate degradation (where it is assigned a high positive score). But why was AAT1 assigned to the asparagine biosynthesis pathway? A closer look at the entry for AAT1 in the SGD database reveals that the curator assigned this enzyme to the pathway based on its enzymatic activity only, which was determined experimentally. In the literature AAT1 is associated with aspartate degradation. Obviously synthesis and degradation cannot appear at the same time and hence the anti-correlation between AAT1 and genes ASN1 and ASN2. This is a clear example of the assignment problem, suggesting that even curated assignments can be further improved using our method. The metabolic network model [18] confirms the previous conclusions. This pathway has two reactions that are entirely contained in the "Alanine and aspartate metabolism" group, which has 15 reactions (see Figure 7). There are 3 instances of the reaction 2.6.1.1 in the network model, one in peroxisome (catalyzed by AAT2), the second in cytoplasm (also catalyzed by AAT2) and the third in mitochondrion (catalyzed by AAT1) (see Figure 7). On the other hand, the reaction 6.3.5.4 takes place in cytoplasm. The expression profiles are in agreement with these subcellular locations and indeed the cytoplasm genes ASN1/ASN2 are highly correlated with AAT2, while anti-correlated with the mitochondrion AAT1.

### Genome wide results

We repeated our analysis, this time with a larger set of pathways from the MetaCyc database, to generate genome wide assignment of genes to pathways. Most pathways were not represented in the Yeast genome, and we restricted our analysis to pathways for which we could assign genes to all reactions (64 pathways). We eliminated pathways that had only one fully characterized reaction since our algorithm is based most dominantly on expression similarity, and therefore assumes at least two reactions in a pathway. Reactions with incomplete EC number or which could not be assigned to a gene in the Yeast genome were ignored. In total, 52 pathways were considered.

We ran our procedure using the two different expression data sets (see the 'Data sets' section). The results are summarized in Table 3, where the pathways are divided into four categories based on their assignment score. Note that the majority of the pathways is assigned a high positive score > 4, indicating strong correlation between the expression profiles of members in these pathways, and supporting our very initial assumption. Only a few pathways are assigned negative scores. These are usually short pathways where one gene is highly anti-correlated with the others. It should be noted that both expression datasets generate very similar results. However, since the Rosetta data set is based on many more experiments than the time-series data set, the pairwise expression similarity scores are much more significant, resulting in higher pathway assignment scores (results not shown). In other words, our confidence in the assignments is stronger with the Rosetta dataset (detailed information about assignments is available at [48] and will be later made available at the Biozon website at [41].

**Table 3:** Distribution of pathway assignment scores. For each data set we ran our algorithm for pathway assignment. The algorithm considers all pathways simultaneously attempting to maximize expression similarity while minimizing the number of conflicts. The final normalized pathway assignment scores *Score*(**A**(*P*)) are divided into four categories based on the average expression similarity of their genes: strongly correlated genes (4 ≤ *Score*), mildly correlated genes (1 <*Score *< 4), weakly or uncorrelated genes (-1 ≤ *Score *≤ 1) and anti-correlated genes (*Score *< -1).

Data Set	Assignment *Score *< -1	score -1 ≤ *Score *≤ 1	1 <*Score *< 4	4 ≤ *Score*
Time-series	4	5	11	32
Rosetta	2	2	7	41

Lastly, it is interesting to compare the assignments before and after resolving conflicts. The pathway relation graph for the 52 pathways contains 10 connected components and 30 singletons. When using the time-series data to assign genes to pathways, we observe conflicts for 9 connected components. The final conflict graph contains 9 connected components and possibly 12 resolvable conflicts (shared edges). 12 of these conflicts are resolved with a small decrease in the assignment score, as is reported in Table 4. Information on the final assignments is given in Table 5. Overall, only a few additional negative pairs are reported after conflicts are resolved, with 32 of the 52 pathways consisting solely of positively scoring pairs (compared to 33 pathways, before conflicts are resolved).

**Table 4:** Genome wide analysis. Connected components' scores before and after resolving conflicts. For each component we list the names of the constituent pathways, the number of conflicts (shared assignments) and the component score. Note that not all conflicts are solvable. For example, the first connected component contains three pathways, and the best initial assignment results in 6 conflicts. Of these only two are solvable (i.e. there are multiple enzymes that can be assigned to these reactions). The final assignment resolves these conflicts while reducing the score of the connected component only slightly (9.31 compared to 10.22).

Component Number	Pathways	Number of Conflicts (solvable conflicts)		Component score	
		
		Before	After	Before	After
1	isoleucine biosynthesis I valine biosynthesis leucine biosynthesis	6(2)	4	10.22	9.31
2	aerobic glycerol degradation II glycolysis	5(3)	2	7.58	7.22
3	asparagine biosynthesis I glutamate – aspartate pathway glutamate degradation VI aspartate biosynthesis II aspartate biosynthesis and degradation	4(0)	4	6.82	6.82
4	trehalose anabolism galactose metabolism UDP-glucose conversion trehalose biosynthesis	4(1)	3	6.64	6.62
5	pentose phosphate pathway, Mycoplasma pneumoniae ribose degradation non-oxidative branch of the pentose phosphate pathway	5(2)	3	5.60	4.78
6	serine biosynthesis cysteine biosynthesis II	3(0)	3	3.98	3.98
7	glycine biosynthesis I glycine cleavage folic acid biosynthesis	3(2)	1	3.41	3.41
8	arginine biosynthesis, Bacillus subtilis de novo biosynthesis of pyrimidine ribonucleotides	0(0)	0	2.08	2.08
9	alanine degradation 3 alanine biosynthesis II	1(1)	0	0	0
10	phenylalanine biosynthesis I tyrosine biosynthesis I	2(1)	1	-1.31	-1.33

**Table 5:** Genome wide analysis. Statistics of the final assignments. For each pathway we list the number of possible assignments, the maximum and minimum scores observed over these assignments, and the final score (note that the final score might not be the maximum score, due to conflicts that were resolved at the refinement stage). The last column gives the number of pairwise relations considered in each assignment, and the number of negative-scoring pairs in the final assignment (in parentheses). Negative scores indicate anti or no correlation. Pathways are sorted by the final assignment score. Note that most pathways are assigned a high positive score, and almost all pairs in the final assignments are positive pairs.

Pathway	Number of Assignments	Max Score	Min Score	Final Score	Number of pairs (negative Pairs)
pentose phosphate pathway, Mycoplasma pneumoniae	2	11.03	-0.73	11.03	1(0)
sulfate assimilation 2	1	11.02	11.02	11.02	1(0)
methionine and S-adenosylmethionine synthesis	2	10.45	7.34	10.45	1(0)
isoleucine biosynthesis I	12	10.32	3.00	10.32	10(0)
valine biosynthesis	4	10.14	4.99	10.14	6(0)
trehalose biosynthesis	2	10.02	9.65	9.65	1(0)
glutamate degradation I	1	9.64	9.64	9.64	3(0)
arginine biosynthesis I	1	9.58	9.58	9.58	3(0)
chorismate biosynthesis	2	9.24	8.19	9.24	21(0)
glycolysis	180	8.98	3.16	8.87	28(0)
asparagine biosynthesis I	4	8.80	-4.88	8.80	1(0)
trehalose anabolism	8	8.80	0.27	8.80	6(0)
proline biosynthesis I	1	8.43	8.43	8.43	3(0)
galactose metabolism	4	7.91	4.62	7.91	6(0)
glycine degradation III	2	7.73	7.73	7.73	1(0)
methylglyoxal degradation	2	7.59	0.98	7.59	1(0)
tRNA charging pathway	49152	7.41	1.69	7.41	171 (2)
glyoxylate cycle	72	7.37	0.27	7.37	10(0)
homoserine methionine biosynthesis	1	7.33	7.33	7.33	1(0)
pyruvate dehydrogenase	2	6.43	4.33	6.43	1(0)
removal of superoxide radicals	4	5.93	-0.45	5.93	1(0)
aerobic glycerol degradation II	180	6.19	1.64	5.58	28(1)
aspartate biosynthesis II	4	4.85	0.75	4.85	1(0)
non-oxidative branch of the pentose phosphate pathway	8	4.82	0.84	4.82	10(1)
oxidative branch of the pentose phosphate pathway	6	4.78	1.07	4.78	3(0)
arginine biosynthesis, Bacillus subtilis	3	4.55	2.69	4.55	34(4)
leucine biosynthesis	4	10.08	4.01	4.31	3(0)
UDP-N-acetylglucosamine biosynthesis	1	4.22	4.22	4.22	1(0)
cysteine biosynthesis II	2	4.19	0.94	4.19	6(0)
tryptophan biosynthesis	2	4.13	3.99	4.13	10(1)
glutamate biosynthesis I	2	4.08	-4.88	4.08	1(0)
glutathione biosynthesis	1	4.03	4.03	4.03	1(0)
arginine degradation I	1	4.00	4.00	4.00	3(0)
arginine proline degradation	1	3.84	3.84	3.84	3(0)
serine biosynthesis	2	3.58	-0.58	3.58	3(0)
folic acid biosynthesis	48	3.49	0.23	3.49	55 (12)
histidine biosynthesis I	1	2.48	2.48	2.48	12(2)
purine biosynthesis 2	16	2.43	2.01	2.43	90 (27)
homocysteine and cysteine interconversion	2	2.35	2.01	2.35	3(1)
biotin biosynthesis I	1	2.27	2.27	2.27	3(2)
homocysteine degradation I	1	2.01	2.01	2.01	1(0)
glutamate degradation VIII	1	1.86	1.86	1.86	8(2)
homoserine biosynthesis	1	1.14	1.14	1.14	3(1)
threonine biosynthesis from homoserine	1	0.87	0.87	0.87	1(0)
de novo biosynthesis of pyrimidine ribonucleotides	12	0.14	-0.69	0.14	43 (25)
ornithine spermine biosynthesis	2	-0.24	-2.48	-0.24	3(2)
tyrosine biosynthesis I	2	-0.53	-0.58	-0.58	3(1)
glycine biosynthesis I	2	-0.91	-3.60	-0.91	1(1)
UDP-glucose conversion	4	-1.32	-2.04	-1.32	3(2)
ribose degradation	2	8.06	-1.74	-1.74	1(1)
phenylalanine biosynthesis I	2	-2.09	-2.80	-2.09	3(2)
tryptophan kynurenine degradation	1	-2.46	-2.46	-2.46	1(1)

## Conclusion

Ongoing sequencing and annotation efforts produce a wealth of data consisting of genes and their products. On the other hand, new types of biological data such as expression and interaction data provide new insights into the mechanisms governing cellular activity. In this light, data integration is necessary in order to accurately analyze the function of genes and other biological entities. The study of biochemical pathways is especially central to these efforts.

Information on cellular pathways is available for several genomes that were studied extensively. However, for most genomes pathway information is not available, what triggered the development of pathway prediction algorithms. Pathway prediction is a difficult problem; Since pathways are not a physical entity, there is no consensus on the definition of a pathway. The pathways that are defined by databases like BioCyc are small subgraphs of a large network of reactions. However, in reality these pathways do not function independently but are rather linked and coordinated with other subnetworks. In an attempt to understand the processes involving metabolism the network has been traditionally divided into smaller subnetworks that can be associated with specific functions. These subnetworks can be considered as the building blocks of the metabolic network and the whole network can be partially reconstructed by integrating the metabolic knowledge contained in these pathways.

In attempt to extrapolate metabolic pathways from one organism to another, several studies developed procedures for assigning genes to pathways. However these procedures ambiguously assign genes to pathways as they usually rely solely on the enzyme class of genes and therefore assign each gene to all the pathways that contain the reactions it can catalyze.

In this paper we present an algorithm for accurate assignment of genes to pathways that attempts to eliminate this ambiguity. For this task, our algorithm utilizes expression data. It has been argued that the metabolic network is co-expressed locally, and an enzyme is co-expressed with the genes catalyzing reactions upstream and downstream of the reaction it catalyzes. We further assume that for the most part pathways are local neighborhoods in the metabolic network and therefore genes assigned to each pathway tend to be co-expressed. Based on this premise, our algorithm assigns genes by maximizing the co-expression of genes that participate in the same pathway. Our algorithm addresses the assignment problem on a genome level, by simultaneously optimizing the co-expression scores for multiple pathways while minimizing the number of conflicts (genes that are shared between different pathways). Our assumption is that if there are multiple genes that can catalyze the same reaction, and that reaction is used in multiple pathways, then each gene is optimized for a different pathway. Conflicts that are detected after initial assignment are reconsidered and our algorithm proceeds by refining the assignment of genes to pathways within connected components in the pathway conflict graph.

Our tests show that for most pathways it is possible to identify a group of genes that can catalyze the pathway reactions and are similarly expressed. Our algorithm can find the most probable assignment of specific genes for each pathway, detect erroneous assignments and suggest control mechanisms of pathways, given a specific expression dataset. The algorithm tackles also the special case of multi-functional enzymes. Since it is difficult to analyze the global network, an alternative approach to detecting pathways of prescribed functions is to search for subnetworks or local neighborhoods in the metabolic network that consist of co-expressed genes, regardless of pathway blueprints. Finding the co-expressed subnetworks of a metabolic network is the methodology of [49] and other studies (as discussed on 'Related Studies' in the paper). However, while this assumption is valid in general it does not always hold and unfortunately these co-expressed subnetworks do not necessarily correspond or overlap with known pathways (as is also indicated by some of our examples). This discrepancy makes it difficult to assess and compare pathway prediction algorithms.

The manually curated pathways that are stored in databases such as MetaCyc and SGD provide an excellent benchmark and perhaps the most accurate reflection of the existing biochemical knowledge, as of today. Our goal is extrapolate that knowledge when predicting pathways in organisms that haven't been studied so far and refine procedures that rely on pathway blueprints and use just EC numbers. Since our algorithm does not rely on manual analysis, it can be most successfully applied to the genomes of organisms that have not fully characterized, once expression data for these genomes becomes available. With the pace in which new genomes are revealed it would be impossible to peruse manual analysis for all and the need for automated procedures becomes evident. The examples we provided prove the effectiveness of our method.

While our algorithm makes advances in the field of pathway prediction it is also faced with several problems. For example, when isozymes are similarly expressed our method picks the best assignment (given the expression data) and only one isozyme is associated with every reaction. However, in some cases multiple isozymes might participate in the same pathway in response to slightly different conditions and substrates. Future versions of our algorithm will handle these cases and estimate the affinity of each isozyme to each pathway that contains the corresponding reaction.

A host of other problems add to the ambiguity of gene-to-pathway assignments, not all them can be addressed with expression data. For example, some enzymes have low specificity and can accept diverse substrates and therefore participate in several different reactions. On the other hand an EC number might specify not a single reaction but rather a class of reactions having common characteristics. One such example is the alcohol dehydrogenases which oxidize a variety of alcohols. The corresponding EC number 1.1.1.1 represents the class of reactions in which either a primary or a secondary alcohol is oxidized, and all alcohol dehydrogenases are annotated with the EC number 1.1.1.1. In yeast there are 6 enzymes annotated with 1.1.1.1. These are the genes ADH1, ADH2, ADH3, ADH4, ADH5 and SFA1. All ADH genes can catalyze the reactions reducing the aldehydes indole acetaldehyde, phenylacetaldehyde and acetaldehyde into the respective alcohols (indole-3-ethanol, phenylethanol and ethanol). However, SFA1 takes as substrate only indole-3-ethanol and phenylethanol. Therefore, as this example demonstrates, EC numbers might not be specific enough, and even database annotations may not be sufficient to differentiate between the different functions of these enzymes.

Our method uses a collection of data sets, including pathways, expression data and statistical models of protein families. We intend to augment these data sets with other relevant biological data sets. For example, integration of interaction data and regulator-regulatee data is necessary in order to predict the global structure of pathways correctly in situations as the one described in 'Discussion' for the isoleucine biosynthesis pathway. Future versions of our algorithm will also account for the topology of the network within pathways and the subcellular location of genes. Other future enhancements include better methods for prediction of enzyme domain families from sequence, to detect new candidates for assignments (thus improving the accuracy of our method) and better mapping procedures from protein annotations to reactions. It should be noted though that our method can be easily extended to other pathways with non-enzymatic reactions. Finally, we are working on probabilistic algorithms which are based on the Expectation-Maximization algorithm, to predict simultaneously gene functions, the existence of pathways, and gene assignments.

## Methods

### Data sets

#### Pathways

As the pathway blueprints we used the set of 468 pathways in the MetaCyc database [6] as of May 2003. This database contains a complete biochemical description of pathways that are observed in different organisms. These descriptions are used as templates when predicting similar pathways in other organisms. We extracted from these descriptions the composition of a pathway as a collection of EC classes. It should be noted that most of the pathways in the MetaCyc database were observed experimentally and are linear as opposed to the pathways in KEGG where a reference pathway might integrate the metabolic information from multiple alternative pathways.

#### Expression data

We used two different expression data sets. The first is the publicly available cell-cycle data set from the Saccharomyces cerevisiae website [26,40]. From this data set we extracted four time series of synchronized S. cerevisiae cells going through the cell cycle. In our analysis each ORF is represented by an extended expression profile derived by concatenating these time series together. The dimensions of these expression vectors range from 1 to 73. This data set has been normalized by [26] to correct for experimental variation between the different microarrays. The second set is the Rosetta Inpharmatics Yeast compendium data [27]. This data set consists of 300 different conditions, mostly deletion mutants. We refer to this set as the Rosetta data set.

#### Sequence data

Our sequence data is the set of protein sequences in the Yeast sequence database with a total of 6298 proteins. Almost all (5894 out of 6298) of the ORFs in the expression data sets can be mapped to genes in the Yeast sequence database through the ORF label.

#### Enzyme families

Each pathway is associated with a set of families, usually a list of enzyme families with their enzyme classification (EC) numbers. To assign proteins to EC families we use a composite non-redundant (NR) database that contains more than 1 million unique sequence entries compiled from more than 20 different databases (the database is available at Biozon [41]). Based on the annotations in these databases, 71,638 proteins can be assigned to one (or more) of 2051 EC families. A total of 70,397 are assigned to a single enzyme family, 1241 are possibly multi-domain proteins with at least two different EC designations, and 498 are ambiguous (or suspicious) in the sense that different databases assign them to different EC families (but within the same level of the EC hierarchy, i.e. the first two levels are identical).

To assign Yeast genes to EC families we match the Yeast sequence database against this composite database. Of the 6298 Yeast genes, 832 can be assigned an EC number, either based on their annotation or the annotation of entries with identical sequences from the other databases. Of these genes, 27 are proteins with multiple enzymatic domains.

#### Predicted EC membership

We extend the set of enzymes by creating a model (sequence profile) for each EC family. The profiles are generated by first grouping proteins with the same, known EC designation from the Biozon NR database. For each EC family we then use an iterative PSI-BLAST procedure [42] to generate a profile. It should be noted that in most cases several profiles are needed to cover all members of the protein family. This is because of the large sequence diversity observed in enzyme families, some of which are composed of several subfamilies that do not exhibit any apparent sequence similarity [22]. Of the 2051 EC families, 597 are composed of more than one subfamily. These models are searched against the Yeast genome, and all genes that are detected as similar with evalue < 0.001 are assigned to the corresponding family, with a confidence value that depends on the evalue.

### Metrics

In a previous study [43] we analyzed and assessed the sensitivity and accuracy of different measures of similarity between expression profiles. The measures were assessed in terms of their ability to detect functional links between genes, such as protein-protein interactions, pathway membership, promoter co-regulation, and sequence homology. Our analysis showed that the z-score based measure that combines the Pearson correlation and the Euclidean metric has the maximal information content. Formally, given two expression vectors **V **and **U **of dimension d, denote by *Dist*(**V**, **U**) the normalized Euclidean metric



and denote by *Corr*(**V**, **U**) the Pearson correlation of the two vectors



The two distance measures are converted to zscores based on the permutation method described in [43]. This method provides reliable measure of significance as it adjusts to the "compositions" of the vectors compared. The zscores are then summed to determine the final similarity score. Since higher correlation scores are assigned positive zscores, and smaller Euclidean distances are assigned negative zscores, the final score is defined as

*sim*(**V**, **U**) = *Z *[*Corr*(**V**, **U**)] - *Z *[*Dist*(**V**, **U**)]

with higher scores indicating stronger similarity. For analysis and performance evaluation see [43]

## Appendix – Related work

Metabolic processes make up a substantial part of the cell's activity, and therefore much of the research on pathways so far focused on creating new databases for metabolic pathways as well as extrapolating the known biochemical information from one organism to other. The goal of this research goes beyond just storing, analyzing and extrapolating the metabolic information and strives to improve the known data by discovering variations to pathways in different organisms as well as to discover novel pathways. In this section we review the literature on the main pathway databases and metabolic pathway reconstruction methods and especially methods that use microarray expression data to analyze pathways.

### Pathway databases

Most pathway databases were created by compiling metabolic information from different literature sources. Among the first such databases was the Enzymes and Metabolic Pathways (EMP) database [1,50]. It contained information about enzymes and metabolic pathways from over 10000 journal articles, and as of 1996 it stored 2180 pathways from about 1400 organisms. This database was later replaced by the Metabolic Pathways Database (EMP/MPW) [2]. The latter was used as the reference database for metabolic pathway prediction in the WIT ("What Is There?") system [3]. This collection contains 2800 pathway diagrams and their logical structure is encoded in terms used for electronic circuits. Another metabolic database is KEGG [5,51,52]. This database is represented as a graph structure based on binary relations between data items [53]. The pathway database consists of more than 200 reference diagrams taken from the biochemical charts that represent all known realizations of a pathway. The database has three parts: the pathway part, the genes part and the reaction and compound part [54]. BioCyc [55] is composed of a family of databases called Pathway Genome databases (PGDB) where each one is centered around a specific genome. The exception is MetaCyc [6,56] that contains over 491 pathways from multiple organisms.

It is also worth mentioning The University of Minnesota Biocatalysis/Biodegradation Database (UM-BBD) that specializes microbial catabolic metabolism of xenobiotic organic compounds [4,57]. Other pathway databases include aMAZE [58], NCGR PathDB [59], ExPASy – Biochemical Pathways [60] and Biocarta [61].

### Pathway prediction based on pathway blueprints

One approach for pathway prediction/recovery in a new organism is based on associating genes that encode enzymes with blueprints of pathways collected either from biochemical charts or from actually observed pathways in different organisms. For example, in order to predict pathways in new genomes, WIT matches the identified enzymes in that genomes with the pathway diagrams from the MPW database [3]. Recently WIT was extended in systems like PUMA2, SEED and ERGO. For example, PUMA2 [62] uses comparative evolutionary analysis of genomes and MPW pathways to extrapolate pathways to new genomes. SEED [8] is an open source system for manual genome annotation where experts can annotate a specific subsystem in multiple genomes at once. It is built around the notion of a "molecular subsystem" which is a collection of functional roles that together fulfill a function. ERGO [7] is a private domain tool that is based on similar principles, and integrates different types of data such as genomic data, regulatory data and essentiality data. No details are available as for the procedures that are used for functional annotation or pathway reconstruction. KEGG matches enzymes to the reference pathways and depending on the degree of completion it assumes that the pathway exists or not [63]. PathFinder [64] is a system that predicts and visualize pathways using the KEGG pathway blueprints using a similar methodology. In UM-BBD, biodegradation pathways of chemical compounds are predicted using a knowledge-based system that matches the compound to a set of biotransformation rules [65]. A biotransformation rule is composed of a sequence of biotransformation functions that transform a compound into its products. The prediction is completed when the resulting compound can no longer be transformed using the rules in the knowledge base or it is one of the termination compounds. In BioCyc, the MetaCyc database is used as the blueprint for the pathway prediction software Pathologic [46,66] which matches enzyme coding genes in a specific genome to reactions in known pathways (but unlike KEGG, they do not re-annotate genes but rather use only existing annotations). Applying Pathologic on a genome results in the creation of a computationally derived PGDB. After creation, a PGDB is curated by mining the literature and new pathways are studied and added to the database. The curated pathways are integrated into MetaCyc to improve the diversity of the database.

All these programs try to address also the problem of finding missing enzymes either by considering alternative reactions or by looking for similar proteins based on sequence similarity or using machine learning models [20-22,24].

### Reconstructing pathways from metabolic networks

Reconstructing pathways from metabolic networks is an emerging direction in pathway prediction that does not use the previously known pathway blueprints. This approach uses existing knowledge on reactions and enzymes and chemical rules to create a complete graph of a possible metabolic network, where pathways are defined as sequences of reactions that transform a metabolite into another. For example, in [9] each metabolite is considered a state and a reaction is considered as a transformation from one state to another. The reactions are compiled from the KEGG Ligand database [54]. This state space is searched heuristically for pathways that link metabolites using the A* algorithm with a cost function that is based on the chemical efficiency of the pathway. Similarly, in [10] the metabolic information is structured as a directed graph with two types of nodes: participants (substrates, enzymes) and events (reactions), and edges link reactions to their constituents. Reactions are weighted with the probability that an enzyme catalyzing this reaction exists in the input genome, using sequence similarity. This graph is then searched for maximally weighted pathways using a depth-first strategy. A similar graph is built in [11], who assert pathways from clusters of co-regulated genes that correspond to connected subgraphs. In [12] the authors represent the metabolic information as Petri nets which are bipartite graphs where nodes are of two types: places and transitions. Reactions correspond to transitions and metabolites to places. Pathways are then generated as sequences of transitions in these Petri nets. Related to the prediction of pathways is the analysis of the topological properties of metabolic networks [67]. They shows that metabolic networks from different organisms have the same scaling properties. Furthermore these networks comply with the design principles of scale-free networks.

Similar principles were used in several studies that constructed genome-wide metabolic networks for organisms such as Escherichia coli [13-15], Haemophilus infiuenzae [16], Helicobacter pylori [68], and Saccharomyces cerevisiae [17,18]. In contrast to the automatic methods described above, these studies were based on manual analysis of multiple data sources and mostly the literature. Though time-consuming and expensive, manual analysis is also more accurate and the constructed networks enabled realistic simulations of metabolic networks. For example, in [17] the Saccharomyces cerevisiae metabolic network is reconstructed and its basic features are analyzed. The information was compiled from databases such as KEGG, YPD, SGD and the literature and was augmented with manual functional annotations. The pathways in the model are compartmentalized between cytosol, mitochondria and extra-cellular, and transport steps are added to the model. Extended information about the reactions such as stoichiometry, reversibility and cofactors is also added to the model in order to facilitate the analysis later on. This model is extensively analyzed in [69] and the phenotype of yeast is simulated using a procedure that considered stoichiometric, thermodynamic and reaction capacity constraints. They tested the effect of gene loss and different growth media on the network viability. Most of the simulations were in agreement with the experimental data. In [18] the model is extended by fully compartmentalizing the metabolic reactions by adding five more cellular locations to the model and revising functional assignments for gene products. They also refine the definitions of reactions to include factors such as mass conservation and charge balance. Their results were quite consistent with the experimental data.

### Expression data and pathway prediction

Another approach to pathway prediction is based on the analysis of expression data. The main idea behind this approach is that genes participating in the same cellular process are functionally interconnected and this interconnection can be induced from expression data by clustering (e.g. [70]). For example, in [31] the authors cluster genes using expression data, and if multiple genes from a cluster belong to a certain pathway they infer that the other members of the cluster might also belong there. Clustering is also used in [32]. The authors define a distance function between enzyme coding genes that is a combination of the distance between the two reactions they catalyze in the pathway reaction graph, and the correlation-distance between their expression profiles. Similarly, in [33] the expression data and the metabolic information is encoded into two kernel functions and canonical correlation analysis is used to search for correlations between pathways and expression data and therefore identify active pathways. The work is extended in [71], by including a kernel function based on protein-protein interactions. An approach for filing holes in pathways based on expression data is presented in [23]. In this paper a scoring function based on a distance function between expression profiles and the topology of the metabolic network is used to score candidate genes.

Module discovery from expression data is another approach to pathway prediction related to clustering. The assumption is that each cellular process is a module involving multiple genes that are co-regulated and hence are co-expressed. Moreover, the same gene may participate in more than one process (module) and therefore each process accounts for a fraction of the genes expression at a particular measurement. In [72], a probabilistic relational model for each processes is defined and an algorithm to train it is introduced. A similar model based on combined expression data and protein-protein interaction data is developed in [34]. A model for the discovery of transcriptional modules and their common binding site motifs as well as the learning algorithm is developed in [73]. The work is extended to co-regulated gene modules and their regulation program (a small common set of regulators) in [74]. In that work the modules were considered disjoint but in [75] a new model is developed which considers overlapping processes and tries to find the regulation program for each process. All the above models are probabilistic graphical models that employ EM like learning methods.

A different approach is taken in [49], who focus on metabolites as the driving force behind the evolution of metabolic regulation. They search for metabolites around which the most significant transcriptional changes occur (as measured by the expression data of the genes that catalyze reactions in which this metabolite is involved) and identify significantly correlated subnetworks of enzymes. In [25], the authors study regulation in metabolic networks and construct a hierarchy of pathways based on their mutual correlation as measured by expression data. The authors also suggest that correlation in expression profiles is an indication of linear pathways that consist of sequences of reactions. Different isozymes might be independently co-regulated with different groups of genes and therefore might be used to switch between the alternate routes or in the differential regulation of reactions that are shared between different pathways. Expression data was used not only for pathway prediction but also in pathway analysis. Efforts for integrating expression data with metabolic information started by trying to visualize the expression data on top of the pathways diagrams. In Pathway Processor [36] the system tries to assess the probability that the expression of a large number of genes in any given pathway is significantly changed in a given experiment and each pathway is scored using this probability. Similarly, MAPPFinder [76] annotates the GO hierarchy with expression data. The method first associate the GO terms with genes and then calculates the percentage of the genes that meet a user specified criterion. A zscore is computed in order to quantify the significance of the obtained percentage. PathMAPA [77] is a system that visualizes metabolic pathways and expression data in Arabidopsis Thaliana, where pathways are represented in terms of enzymes annotated with EC numbers. The tool estimates the significance of a pathway being up regulated or down regulated in a given experiment. In [35] the authors suggest three functions to score pathways: based on the activity of the genes in the pathway, co-regulations of the genes and the topology of the pathway. The method is then applied to putative pathways in the KEGG database in order to asses the biological significance of these pathways. In [37] the authors present a scoring method for classes of genes. These classes are based on Gene Ontology classification and the scoring is based on expression data. Three types of scores are proposed: co-expression of genes in the same class, statistical significance of gene expression changes, and the learnability of the classification. The scores are converted to p-values to assess their statistical significance, in search of classes with significant scores. Similarly, the biological significance of the pathways asserted in [12] (see previous subsection) is computed by using a scoring function based on expression data in [30]. They score both pathways and genes using two different types of scores (conspicuousness of the expression profile and the synchrony), and the scores are used to asses which are the most probable pathways. Another pathway scoring approach was developed in [38], in search of active pathways. This approach scores a gene set (the set of genes which catalyze reactions in a pathway) by summing all pairwise similarity of the genes in the set. The score obtained is then transformed to a pvalue. All these approaches are related to our approach. However, our method does not score pathways but rather it scores gene assignments to determine the best assignment and identify alternative assignments. Furthermore, our algorithm is geared toward simultaneous prediction of multiple pathways while minimizing shared assignments.

## Authors' contributions

LP implemented the model, ran experiments, compared to other models and analyzed the result sets. GY conceived of the study, designed the model and analyzed the results.

**Table 6:** The correspondence of genes to Biozon NR identifiers. We refer to genes using their unique and stable Biozon NR identifiers, at  [41]. To view an entry with identifier *x *follow the URL: *x*.

Gene	NR Identifiers
ILV1	005760000068
CHA1	003600000165
YKL218C	003260000219
ILV2	003090000098
ILV6	006870000019
ILV5	003950000069
ILV3	005850000040
BAT1	003930000034
BAT2	003760000122
FOL2	002430000075
FOL1	008640000008
FOL3	004270000071
DFR1	002110001504
MIS1	009750000001
ADE3	009460000003
SHM1	005650000392
SHM2	004690000046
YKL132C	004300000053
MET7	005480000035
AAT1	004510000006 004510000730
AAT2	004320000601 004170000010
ASN2	005720000349 005710000020
ASN1	005720000348 005710000019

## Supplementary Material

Additional File 1 Assignments of genes to pathways with the time series dataset. For each pathway we list the 10 highest scoring and the 10 lowest scoring assignments (or all assignments, if the number of assignments is 100 or less). Click here for file

Additional File 2 Assignments of genes to pathways with the rosetta dataset. For each pathway we list the 10 highest scoring and the 10 lowest scoring assignments (or all assignments, if the number of assignments is 100 or less). Click here for file
