# Supplementary material for: Automation of gene assignments to metabolic pathways using high-throughput expression data
Source: BMC Bioinformatics. 2005 Aug 31;6:217. doi: 10.1186/1471-2105-6-217 (PMC1239907; doi:10.1186/1471-2105-6-217)
Supplement: Additional File 1 — Assignments of genes to pathways with the time series dataset. For each pathway we list the 10 highest scoring and the 10 lowest scoring assignments (or all assignments, if the number of assignments is 100 or less). [file 1471-2105-6-217-S1.pdf]

Number	Normalized Score	Number of Pairs	Positive Pairs	Negative Pairs	Zero Pairs	Assignments
1	7.41	171	169	2	19	6.1.1.14 : YBR121C<br>6.1.1.21 : YPR033C<br>6.1.1.6 : YDR037W<br>6.1.1.10 : YGR264C<br>6.1.1.20 : YLR060W<br>6.1.1.15 : YHR020W<br>6.1.1.11 : YDR023W<br>6.1.1.3 : 2924<br>6.1.1.2 : YOL097C<br>6.1.1.1 : YGR185C<br>6.1.1.12 : YLL018C<br>6.1.1.22 : YHR019C<br>6.1.1.7 : YOR335C<br>6.1.1.9 : YGR094W<br>6.1.1.4 : YPL160W<br>6.1.1.5 : YBL076C<br>6.1.1.17 : YGL245W<br>6.1.1.18 : YOR168W<br>6.1.1.16 : YNL247W<br>6.1.1.19 : YDR341C
2	7.28	153	151	2	37	6.1.1.14 : YBR121C<br>6.1.1.21 : YPR033C<br>6.1.1.6 : YDR037W<br>6.1.1.10 : YGR264C<br>6.1.1.20 : 1887<br>6.1.1.15 : YHR020W<br>6.1.1.11 : YDR023W<br>6.1.1.3 : 2924<br>6.1.1.2 : YOL097C<br>6.1.1.1 : YGR185C<br>6.1.1.12 : YLL018C<br>6.1.1.22 : YHR019C<br>6.1.1.7 : YOR335C<br>6.1.1.9 : YGR094W<br>6.1.1.4 : YPL160W<br>6.1.1.5 : YBL076C<br>6.1.1.17 : YGL245W<br>6.1.1.18 : YOR168W<br>6.1.1.16 : YNL247W<br>6.1.1.19 : YDR341C

Assignments for the pathway ”tRNA charging pathway” (time series data set). There are 49152 possible assignments of genes to reactions(only the 10 highest scoring and the 10 lowest scoring assignments are displayed).

Number	Normalized Score	Number of Pairs	Positive Pairs	Negative Pairs	Zero Pairs	Assignments
3	7.26	171	164	7	19	6.1.1.14 : YBR121C<br>6.1.1.21 : YPR033C<br>6.1.1.6 : YDR037W<br>6.1.1.10 : YGR264C<br>6.1.1.20 : YLR060W<br>6.1.1.15 : YHR020W<br>6.1.1.11 : YDR023W<br>6.1.1.3 : 2924<br>6.1.1.2 : YOL097C<br>6.1.1.1 : YGR185C<br>6.1.1.12 : YLL018C<br>6.1.1.22 : YHR019C<br>6.1.1.7 : YOR335C<br>6.1.1.9 : YGR094W<br>6.1.1.4 : YPL160W<br>6.1.1.5 : YBL076C<br>6.1.1.17 : YOL033W<br>6.1.1.18 : YOR168W<br>6.1.1.16 : YNL247W<br>6.1.1.19 : YDR341C
4	7.10	153	146	7	37	6.1.1.14 : YBR121C<br>6.1.1.21 : YPR033C<br>6.1.1.6 : YDR037W<br>6.1.1.10 : YGR264C<br>6.1.1.20 : 1887<br>6.1.1.15 : YHR020W<br>6.1.1.11 : YDR023W<br>6.1.1.3 : 2924<br>6.1.1.2 : YOL097C<br>6.1.1.1 : YGR185C<br>6.1.1.12 : YLL018C<br>6.1.1.22 : YHR019C<br>6.1.1.7 : YOR335C<br>6.1.1.9 : YGR094W<br>6.1.1.4 : YPL160W<br>6.1.1.5 : YBL076C<br>6.1.1.17 : YOL033W<br>6.1.1.18 : YOR168W<br>6.1.1.16 : YNL247W<br>6.1.1.19 : YDR341C

Assignments for the pathway "tRNA charging pathway" (time series data set). There are 49152 possible assignments of genes to reactions(only the 10 highest scoring and the 10 lowest scoring assignments are displayed).

Number	Normalized Score	Number of Pairs	Positive Pairs	Negative Pairs	Zero Pairs	Assignments
5	7.04	171	164	7	19	6.1.1.14 : YBR121C<br>6.1.1.21 : YPR033C<br>6.1.1.6 : YNL073W<br>6.1.1.10 : YGR264C<br>6.1.1.20 : YLR060W<br>6.1.1.15 : YHR020W<br>6.1.1.11 : YDR023W<br>6.1.1.3 : 2924<br>6.1.1.2 : YOL097C<br>6.1.1.1 : YGR185C<br>6.1.1.12 : YLL018C<br>6.1.1.22 : YHR019C<br>6.1.1.7 : YOR335C<br>6.1.1.9 : YGR094W<br>6.1.1.4 : YPL160W<br>6.1.1.5 : YBL076C<br>6.1.1.17 : YGL245W<br>6.1.1.18 : YOR168W<br>6.1.1.16 : YNL247W<br>6.1.1.19 : YDR341C
6	6.97	171	160	11	19	6.1.1.14 : YBR121C<br>6.1.1.21 : YPR033C<br>6.1.1.6 : YNL073W<br>6.1.1.10 : YGR264C<br>6.1.1.20 : YLR060W<br>6.1.1.15 : YHR020W<br>6.1.1.11 : YDR023W<br>6.1.1.3 : 2924<br>6.1.1.2 : YOL097C<br>6.1.1.1 : YGR185C<br>6.1.1.12 : YLL018C<br>6.1.1.22 : YHR019C<br>6.1.1.7 : YOR335C<br>6.1.1.9 : YGR094W<br>6.1.1.4 : YPL160W<br>6.1.1.5 : YBL076C<br>6.1.1.17 : YOL033W<br>6.1.1.18 : YOR168W<br>6.1.1.16 : YNL247W<br>6.1.1.19 : YDR341C

Assignments for the pathway "tRNA charging pathway" (time series data set). There are 49152 possible assignments of genes to reactions(only the 10 highest scoring and the 10 lowest scoring assignments are displayed).

Number	Normalized Score	Number of Pairs	Positive Pairs	Negative Pairs	Zero Pairs	Assignments
7	6.94	171	159	12	19	6.1.1.14 : YBR121C<br>6.1.1.21 : YPR033C<br>6.1.1.6 : YDR037W<br>6.1.1.10 : YGR264C<br>6.1.1.20 : YLR060W<br>6.1.1.15 : YHR020W<br>6.1.1.11 : YDR023W<br>6.1.1.3 : 2924<br>6.1.1.2 : YOL097C<br>6.1.1.1 : YGR185C<br>6.1.1.12 : YPL104W<br>6.1.1.22 : YHR019C<br>6.1.1.7 : YOR335C<br>6.1.1.9 : YGR094W<br>6.1.1.4 : YPL160W<br>6.1.1.5 : YBL076C<br>6.1.1.17 : YGL245W<br>6.1.1.18 : YOR168W<br>6.1.1.16 : YNL247W<br>6.1.1.19 : YDR341C
8	6.87	153	146	7	37	6.1.1.14 : YBR121C<br>6.1.1.21 : YPR033C<br>6.1.1.6 : YNL073W<br>6.1.1.10 : YGR264C<br>6.1.1.20 : 1887<br>6.1.1.15 : YHR020W<br>6.1.1.11 : YDR023W<br>6.1.1.3 : 2924<br>6.1.1.2 : YOL097C<br>6.1.1.1 : YGR185C<br>6.1.1.12 : YLL018C<br>6.1.1.22 : YHR019C<br>6.1.1.7 : YOR335C<br>6.1.1.9 : YGR094W<br>6.1.1.4 : YPL160W<br>6.1.1.5 : YBL076C<br>6.1.1.17 : YGL245W<br>6.1.1.18 : YOR168W<br>6.1.1.16 : YNL247W<br>6.1.1.19 : YDR341C

Assignments for the pathway "tRNA charging pathway" (time series data set). There are 49152 possible assignments of genes to reactions(only the 10 highest scoring and the 10 lowest scoring assignments are displayed).

Number	Normalized Score	Number of Pairs	Positive Pairs	Negative Pairs	Zero Pairs	Assignments
9	6.84	171	155	16	19	6.1.1.14 : YBR121C<br>6.1.1.21 : YPR033C<br>6.1.1.6 : YDR037W<br>6.1.1.10 : YGR264C<br>6.1.1.20 : YLR060W<br>6.1.1.15 : YHR020W<br>6.1.1.11 : YDR023W<br>6.1.1.3 : 2924<br>6.1.1.2 : YOL097C<br>6.1.1.1 : YGR185C<br>6.1.1.12 : YPL104W<br>6.1.1.22 : YHR019C<br>6.1.1.7 : YOR335C<br>6.1.1.9 : YGR094W<br>6.1.1.4 : YPL160W<br>6.1.1.5 : YBL076C<br>6.1.1.17 : YOL033W<br>6.1.1.18 : YOR168W<br>6.1.1.16 : YNL247W<br>6.1.1.19 : YDR341C
10	6.80	171	166	5	19	6.1.1.14 : YBR121C<br>6.1.1.21 : YPR033C<br>6.1.1.6 : YDR037W<br>6.1.1.10 : YGR264C<br>6.1.1.20 : YLR060W<br>6.1.1.15 : YHR020W<br>6.1.1.11 : YDR023W<br>6.1.1.3 : 2924<br>6.1.1.2 : YOL097C<br>6.1.1.1 : YGR185C<br>6.1.1.12 : YLL018C<br>6.1.1.22 : YHR019C<br>6.1.1.7 : YOR335C<br>6.1.1.9 : YGR094W<br>6.1.1.4 : YPL160W<br>6.1.1.5 : YBL076C<br>6.1.1.17 : YGL245W<br>6.1.1.18 : YOR168W<br>6.1.1.16 : YNL247W<br>6.1.1.19 : YHR091C
...<br>...						

Assignments for the pathway "tRNA charging pathway" (time series data set). There are 49152 possible assignments of genes to reactions(only the 10 highest scoring and the 10 lowest scoring assignments are displayed).

Number	Normalized Score	Number of Pairs	Positive Pairs	Negative Pairs	Zero Pairs	Assignments
49143	1.79	171	105	66	19	6.1.1.14 : YPR081C<br>6.1.1.21 : YPR033C<br>6.1.1.6 : YNL073W<br>6.1.1.10 : YGR171C<br>6.1.1.20 : 1887<br>6.1.1.15 : YER087W<br>6.1.1.11 : YDR023W<br>6.1.1.3 : YKL194C<br>6.1.1.2 : YDR268W<br>6.1.1.1 : YPL097W<br>6.1.1.12 : YLL018C<br>6.1.1.22 : YCR024C<br>6.1.1.7 : YOR335C<br>6.1.1.9 : YGR094W<br>6.1.1.4 : YLR382C<br>6.1.1.5 : YPL040C<br>6.1.1.17 : YGL245W<br>6.1.1.18 : YOR168W<br>6.1.1.16 : YNL247W<br>6.1.1.19 : YDR341C
49144	1.78	171	107	64	19	6.1.1.14 : YPR081C<br>6.1.1.21 : YPR033C<br>6.1.1.6 : YDR037W<br>6.1.1.10 : YGR171C<br>6.1.1.20 : 1887<br>6.1.1.15 : YER087W<br>6.1.1.11 : YDR023W<br>6.1.1.3 : YKL194C<br>6.1.1.2 : YDR268W<br>6.1.1.1 : YPL097W<br>6.1.1.12 : YLL018C<br>6.1.1.22 : YCR024C<br>6.1.1.7 : YOR335C<br>6.1.1.9 : YGR094W<br>6.1.1.4 : YLR382C<br>6.1.1.5 : YPL040C<br>6.1.1.17 : YGL245W<br>6.1.1.18 : YOR168W<br>6.1.1.16 : YNL247W<br>6.1.1.19 : YHR091C

Assignments for the pathway "tRNA charging pathway" (time series data set). There are 49152 possible assignments of genes to reactions(only the 10 highest scoring and the 10 lowest scoring assignments are displayed).

Number	Normalized Score	Number of Pairs	Positive Pairs	Negative Pairs	Zero Pairs	Assignments
49145	1.78	171	103	68	19	6.1.1.14 : YPR081C<br>6.1.1.21 : YPR033C<br>6.1.1.6 : YDR037W<br>6.1.1.10 : YGR171C<br>6.1.1.20 : YPR047W<br>6.1.1.15 : YER087W<br>6.1.1.11 : YDR023W<br>6.1.1.3 : 2924<br>6.1.1.2 : YDR268W<br>6.1.1.1 : YPL097W<br>6.1.1.12 : YLL018C<br>6.1.1.22 : YCR024C<br>6.1.1.7 : YOR335C<br>6.1.1.9 : YGR094W<br>6.1.1.4 : YLR382C<br>6.1.1.5 : YPL040C<br>6.1.1.17 : YGL245W<br>6.1.1.18 : YOR168W<br>6.1.1.16 : YNL247W<br>6.1.1.19 : YDR341C
49146	1.76	171	103	68	19	6.1.1.14 : YPR081C<br>6.1.1.21 : YPR033C<br>6.1.1.6 : YDR037W<br>6.1.1.10 : YGR171C<br>6.1.1.20 : 1887<br>6.1.1.15 : YER087W<br>6.1.1.11 : YHR011W<br>6.1.1.3 : YKL194C<br>6.1.1.2 : YDR268W<br>6.1.1.1 : YPL097W<br>6.1.1.12 : YLL018C<br>6.1.1.22 : YCR024C<br>6.1.1.7 : YOR335C<br>6.1.1.9 : YGR094W<br>6.1.1.4 : YLR382C<br>6.1.1.5 : YPL040C<br>6.1.1.17 : YGL245W<br>6.1.1.18 : YOR168W<br>6.1.1.16 : YNL247W<br>6.1.1.19 : YDR341C

Assignments for the pathway "tRNA charging pathway" (time series data set). There are 49152 possible assignments of genes to reactions(only the 10 highest scoring and the 10 lowest scoring assignments are displayed).

Number	Normalized Score	Number of Pairs	Positive Pairs	Negative Pairs	Zero Pairs	Assignments
49147	1.74	153	95	58	37	6.1.1.14 : YPR081C<br>6.1.1.21 : YPR033C<br>6.1.1.6 : YNL073W<br>6.1.1.10 : YGR171C<br>6.1.1.20 : 1887<br>6.1.1.15 : YER087W<br>6.1.1.11 : YHR011W<br>6.1.1.3 : 2924<br>6.1.1.2 : YDR268W<br>6.1.1.1 : YPL097W<br>6.1.1.12 : YLL018C<br>6.1.1.22 : YCR024C<br>6.1.1.7 : YOR335C<br>6.1.1.9 : YGR094W<br>6.1.1.4 : YLR382C<br>6.1.1.5 : YPL040C<br>6.1.1.17 : YGL245W<br>6.1.1.18 : YOR168W<br>6.1.1.16 : YNL247W<br>6.1.1.19 : YDR341C
49148	1.73	171	102	69	19	6.1.1.14 : YPR081C<br>6.1.1.21 : YPR033C<br>6.1.1.6 : YDR037W<br>6.1.1.10 : YGR171C<br>6.1.1.20 : 1887<br>6.1.1.15 : YER087W<br>6.1.1.11 : YDR023W<br>6.1.1.3 : YKL194C<br>6.1.1.2 : YDR268W<br>6.1.1.1 : YPL097W<br>6.1.1.12 : YLL018C<br>6.1.1.22 : YCR024C<br>6.1.1.7 : YOR335C<br>6.1.1.9 : YGR094W<br>6.1.1.4 : YLR382C<br>6.1.1.5 : YPL040C<br>6.1.1.17 : YGL245W<br>6.1.1.18 : YOR168W<br>6.1.1.16 : YNL247W<br>6.1.1.19 : YDR341C

Assignments for the pathway "tRNA charging pathway" (time series data set). There are 49152 possible assignments of genes to reactions(only the 10 highest scoring and the 10 lowest scoring assignments are displayed).

Number	Normalized Score	Number of Pairs	Positive Pairs	Negative Pairs	Zero Pairs	Assignments
49149	1.73	153	95	58	37	6.1.1.14 : YPR081C<br>6.1.1.21 : YPR033C<br>6.1.1.6 : YNL073W<br>6.1.1.10 : YGR171C<br>6.1.1.20 : 1887<br>6.1.1.15 : YER087W<br>6.1.1.11 : YDR023W<br>6.1.1.3 : 2924<br>6.1.1.2 : YDR268W<br>6.1.1.1 : YPL097W<br>6.1.1.12 : YLL018C<br>6.1.1.22 : YCR024C<br>6.1.1.7 : YOR335C<br>6.1.1.9 : YGR094W<br>6.1.1.4 : YLR382C<br>6.1.1.5 : YPL040C<br>6.1.1.17 : YGL245W<br>6.1.1.18 : YOR168W<br>6.1.1.16 : YNL247W<br>6.1.1.19 : YDR341C
49150	1.72	153	93	60	37	6.1.1.14 : YPR081C<br>6.1.1.21 : YPR033C<br>6.1.1.6 : YDR037W<br>6.1.1.10 : YGR171C<br>6.1.1.20 : 1887<br>6.1.1.15 : YER087W<br>6.1.1.11 : YDR023W<br>6.1.1.3 : 2924<br>6.1.1.2 : YDR268W<br>6.1.1.1 : YPL097W<br>6.1.1.12 : YLL018C<br>6.1.1.22 : YCR024C<br>6.1.1.7 : YOR335C<br>6.1.1.9 : YGR094W<br>6.1.1.4 : YLR382C<br>6.1.1.5 : YPL040C<br>6.1.1.17 : YGL245W<br>6.1.1.18 : YOR168W<br>6.1.1.16 : YNL247W<br>6.1.1.19 : YDR341C

Assignments for the pathway "tRNA charging pathway" (time series data set). There are 49152 possible assignments of genes to reactions(only the 10 highest scoring and the 10 lowest scoring assignments are displayed).

Number	Normalized Score	Number of Pairs	Positive Pairs	Negative Pairs	Zero Pairs	Assignments
49151	1.72	153	97	56	37	6.1.1.14 : YPR081C<br>6.1.1.21 : YPR033C<br>6.1.1.6 : YDR037W<br>6.1.1.10 : YGR171C<br>6.1.1.20 : 1887<br>6.1.1.15 : YER087W<br>6.1.1.11 : YDR023W<br>6.1.1.3 : 2924<br>6.1.1.2 : YDR268W<br>6.1.1.1 : YPL097W<br>6.1.1.12 : YLL018C<br>6.1.1.22 : YCR024C<br>6.1.1.7 : YOR335C<br>6.1.1.9 : YGR094W<br>6.1.1.4 : YLR382C<br>6.1.1.5 : YPL040C<br>6.1.1.17 : YGL245W<br>6.1.1.18 : YOR168W<br>6.1.1.16 : YNL247W<br>6.1.1.19 : YHR091C
49152	1.69	153	93	60	37	6.1.1.14 : YPR081C<br>6.1.1.21 : YPR033C<br>6.1.1.6 : YDR037W<br>6.1.1.10 : YGR171C<br>6.1.1.20 : 1887<br>6.1.1.15 : YER087W<br>6.1.1.11 : YHR011W<br>6.1.1.3 : 2924<br>6.1.1.2 : YDR268W<br>6.1.1.1 : YPL097W<br>6.1.1.12 : YLL018C<br>6.1.1.22 : YCR024C<br>6.1.1.7 : YOR335C<br>6.1.1.9 : YGR094W<br>6.1.1.4 : YLR382C<br>6.1.1.5 : YPL040C<br>6.1.1.17 : YGL245W<br>6.1.1.18 : YOR168W<br>6.1.1.16 : YNL247W<br>6.1.1.19 : YDR341C

Table 1: **Assignments for the pathway "tRNA charging pathway" (time series data set). There are 49152 possible assignments of genes to reactions(only the 10 highest scoring and the 10 lowest scoring assignments are displayed).**

Number	Normalized Score	Number of Pairs	Positive Pairs	Negative Pairs	Zero Pairs	Assignments
1	6.19	28	27	1	8	1.1.99.5 : YIL155C<br>2.7.1.40 : YAL038W<br>4.2.1.11 : YGR254W<br>5.4.2.1 : YKL152C<br>2.7.2.3 : 639<br>1.2.1.12 : YGR192C<br>5.3.1.1 : YDR050C<br>1.1.1.8 : YDL022W<br>2.7.1.30 : YHL032C
2	5.90	28	27	1	8	1.1.99.5 : YIL155C<br>2.7.1.40 : YAL038W<br>4.2.1.11 : YHR174W<br>5.4.2.1 : YKL152C<br>2.7.2.3 : 639<br>1.2.1.12 : YGR192C<br>5.3.1.1 : YDR050C<br>1.1.1.8 : YDL022W<br>2.7.1.30 : YHL032C
3	5.83	28	27	1	8	1.1.99.5 : YIL155C<br>2.7.1.40 : YOR347C<br>4.2.1.11 : YGR254W<br>5.4.2.1 : YKL152C<br>2.7.2.3 : 639<br>1.2.1.12 : YGR192C<br>5.3.1.1 : YDR050C<br>1.1.1.8 : YDL022W<br>2.7.1.30 : YHL032C
4	5.75	28	27	1	8	1.1.99.5 : YIL155C<br>2.7.1.40 : YAL038W<br>4.2.1.11 : YGR254W<br>5.4.2.1 : YKL152C<br>2.7.2.3 : 639<br>1.2.1.12 : YJR009C<br>5.3.1.1 : YDR050C<br>1.1.1.8 : YDL022W<br>2.7.1.30 : YHL032C
5	5.71	28	27	1	8	1.1.99.5 : YIL155C<br>2.7.1.40 : YAL038W<br>4.2.1.11 : YGR254W<br>5.4.2.1 : YKL152C<br>2.7.2.3 : 639<br>1.2.1.12 : YJL052W<br>5.3.1.1 : YDR050C<br>1.1.1.8 : YDL022W<br>2.7.1.30 : YHL032C

Assignments for the pathway "aerobic glycerol degradation II" (time series data set). There are 180 possible assignments of genes to reactions(only the 10 highest scoring and the 10 lowest scoring assignments are displayed).

Number	Normalized Score	Number of Pairs	Positive Pairs	Negative Pairs	Zero Pairs	Assignments
6	5.58	28	27	1	8	1.1.99.5 : YIL155C<br>2.7.1.40 : YOR347C<br>4.2.1.11 : YGR254W<br>5.4.2.1 : YDL021W<br>2.7.2.3 : 639<br>1.2.1.12 : YGR192C<br>5.3.1.1 : YDR050C<br>1.1.1.8 : YDL022W<br>2.7.1.30 : YHL032C
7	5.53	28	26	2	8	1.1.99.5 : YIL155C<br>2.7.1.40 : YAL038W<br>4.2.1.11 : YGR254W<br>5.4.2.1 : YKL152C<br>2.7.2.3 : 639<br>1.2.1.12 : YGR192C<br>5.3.1.1 : YDR050C<br>1.1.1.8 : YOL059W<br>2.7.1.30 : YHL032C
8	5.50	28	27	1	8	1.1.99.5 : YIL155C<br>2.7.1.40 : YOR347C<br>4.2.1.11 : YHR174W<br>5.4.2.1 : YDL021W<br>2.7.2.3 : 639<br>1.2.1.12 : YGR192C<br>5.3.1.1 : YDR050C<br>1.1.1.8 : YDL022W<br>2.7.1.30 : YHL032C
9	5.49	28	27	1	8	1.1.99.5 : YIL155C<br>2.7.1.40 : YAL038W<br>4.2.1.11 : YHR174W<br>5.4.2.1 : YKL152C<br>2.7.2.3 : 639<br>1.2.1.12 : YJR009C<br>5.3.1.1 : YDR050C<br>1.1.1.8 : YDL022W<br>2.7.1.30 : YHL032C
10	5.43	28	27	1	8	1.1.99.5 : YIL155C<br>2.7.1.40 : YOR347C<br>4.2.1.11 : YGR254W<br>5.4.2.1 : YKL152C<br>2.7.2.3 : 639<br>1.2.1.12 : YJL052W<br>5.3.1.1 : YDR050C<br>1.1.1.8 : YDL022W<br>2.7.1.30 : YHL032C
...<br>...						

Assignments for the pathway "aerobic glycerol degradation II" (time series data set). There are 180 possible assignments of genes to reactions(only the 10 highest scoring and the 10 lowest scoring assignments are displayed).

Number	Normalized Score	Number of Pairs	Positive Pairs	Negative Pairs	Zero Pairs	Assignments
171	2.12	28	18	10	8	1.1.99.5 : YIL155C<br>2.7.1.40 : YOR347C<br>4.2.1.11 : YOR393W<br>5.4.2.1 : YOL056W<br>2.7.2.3 : 639<br>1.2.1.12 : YJR009C<br>5.3.1.1 : YDR050C<br>1.1.1.8 : YOL059W<br>2.7.1.30 : YHL032C
172	2.08	28	17	11	8	1.1.99.5 : YIL155C<br>2.7.1.40 : YAL038W<br>4.2.1.11 : YOR393W<br>5.4.2.1 : YOL056W<br>2.7.2.3 : 639<br>1.2.1.12 : YGR192C<br>5.3.1.1 : YDR050C<br>1.1.1.8 : YOL059W<br>2.7.1.30 : YHL032C
173	2.04	28	18	10	8	1.1.99.5 : YIL155C<br>2.7.1.40 : YOR347C<br>4.2.1.11 : YPL281C<br>5.4.2.1 : YOL056W<br>2.7.2.3 : 639<br>1.2.1.12 : YJL052W<br>5.3.1.1 : YDR050C<br>1.1.1.8 : YOL059W<br>2.7.1.30 : YHL032C
174	1.97	28	18	10	8	1.1.99.5 : YIL155C<br>2.7.1.40 : YAL038W<br>4.2.1.11 : YOR393W<br>5.4.2.1 : YOL056W<br>2.7.2.3 : 639<br>1.2.1.12 : YJL052W<br>5.3.1.1 : YDR050C<br>1.1.1.8 : YDL022W<br>2.7.1.30 : YHL032C
175	1.96	28	18	10	8	1.1.99.5 : YIL155C<br>2.7.1.40 : YAL038W<br>4.2.1.11 : YOR393W<br>5.4.2.1 : YOL056W<br>2.7.2.3 : 639<br>1.2.1.12 : YJR009C<br>5.3.1.1 : YDR050C<br>1.1.1.8 : YDL022W<br>2.7.1.30 : YHL032C

Assignments for the pathway "aerobic glycerol degradation II" (time series data set). There are 180 possible assignments of genes to reactions(only the 10 highest scoring and the 10 lowest scoring assignments are displayed).

Number	Normalized Score	Number of Pairs	Positive Pairs	Negative Pairs	Zero Pairs	Assignments
176	1.92	28	18	10	8	1.1.99.5 : YIL155C<br>2.7.1.40 : YOR347C<br>4.2.1.11 : YPL281C<br>5.4.2.1 : YOL056W<br>2.7.2.3 : 639<br>1.2.1.12 : YJR009C<br>5.3.1.1 : YDR050C<br>1.1.1.8 : YOL059W<br>2.7.1.30 : YHL032C
177	1.83	28	18	10	8	1.1.99.5 : YIL155C<br>2.7.1.40 : YAL038W<br>4.2.1.11 : YPL281C<br>5.4.2.1 : YOL056W<br>2.7.2.3 : 639<br>1.2.1.12 : YJL052W<br>5.3.1.1 : YDR050C<br>1.1.1.8 : YOL059W<br>2.7.1.30 : YHL032C
178	1.79	28	18	10	8	1.1.99.5 : YIL155C<br>2.7.1.40 : YAL038W<br>4.2.1.11 : YPL281C<br>5.4.2.1 : YOL056W<br>2.7.2.3 : 639<br>1.2.1.12 : YJR009C<br>5.3.1.1 : YDR050C<br>1.1.1.8 : YOL059W<br>2.7.1.30 : YHL032C
179	1.65	28	17	11	8	1.1.99.5 : YIL155C<br>2.7.1.40 : YAL038W<br>4.2.1.11 : YOR393W<br>5.4.2.1 : YOL056W<br>2.7.2.3 : 639<br>1.2.1.12 : YJL052W<br>5.3.1.1 : YDR050C<br>1.1.1.8 : YOL059W<br>2.7.1.30 : YHL032C
180	1.64	28	17	11	8	1.1.99.5 : YIL155C<br>2.7.1.40 : YAL038W<br>4.2.1.11 : YOR393W<br>5.4.2.1 : YOL056W<br>2.7.2.3 : 639<br>1.2.1.12 : YJR009C<br>5.3.1.1 : YDR050C<br>1.1.1.8 : YOL059W<br>2.7.1.30 : YHL032C

Table 2: **Assignments for the pathway "aerobic glycerol degradation II" (time series data set). There are 180 possible assignments of genes to reactions(only the 10 highest scoring and the 10 lowest scoring assignments are displayed).**

Number	Normalized Score	Number of Pairs	Positive Pairs	Negative Pairs	Zero Pairs	Assignments
1	8.98	28	28	0	8	5.3.1.9 : YBR196C<br>2.7.1.11 : YGR240C<br>4.1.2.13 : YKL060C<br>5.3.1.1 : YDR050C<br>1.2.1.12 : YGR192C<br>2.7.2.3 : 639<br>5.4.2.1 : YKL152C<br>4.2.1.11 : YHR174W<br>2.7.1.40 : YAL038W
2	8.97	28	28	0	8	5.3.1.9 : YBR196C<br>2.7.1.11 : YGR240C<br>4.1.2.13 : YKL060C<br>5.3.1.1 : YDR050C<br>1.2.1.12 : YGR192C<br>2.7.2.3 : 639<br>5.4.2.1 : YKL152C<br>4.2.1.11 : YGR254W<br>2.7.1.40 : YAL038W
3	8.87	28	28	0	8	5.3.1.9 : YBR196C<br>2.7.1.11 : YGR240C<br>4.1.2.13 : YKL060C<br>5.3.1.1 : YDR050C<br>1.2.1.12 : YJR009C<br>2.7.2.3 : 639<br>5.4.2.1 : YKL152C<br>4.2.1.11 : YHR174W<br>2.7.1.40 : YAL038W
4	8.82	28	28	0	8	5.3.1.9 : YBR196C<br>2.7.1.11 : YGR240C<br>4.1.2.13 : YKL060C<br>5.3.1.1 : YDR050C<br>1.2.1.12 : YJR009C<br>2.7.2.3 : 639<br>5.4.2.1 : YKL152C<br>4.2.1.11 : YGR254W<br>2.7.1.40 : YAL038W
5	8.10	28	28	0	8	5.3.1.9 : YBR196C<br>2.7.1.11 : YGR240C<br>4.1.2.13 : YKL060C<br>5.3.1.1 : YDR050C<br>1.2.1.12 : YJL052W<br>2.7.2.3 : 639<br>5.4.2.1 : YKL152C<br>4.2.1.11 : YGR254W<br>2.7.1.40 : YAL038W

Assignments for the pathway "glycolysis" (time series data set). There are 180 possible assignments of genes to reactions(only the 10 highest scoring and the 10 lowest scoring assignments are displayed).

Number	Normalized Score	Number of Pairs	Positive Pairs	Negative Pairs	Zero Pairs	Assignments
6	8.10	28	28	0	8	5.3.1.9 : YBR196C<br>2.7.1.11 : YGR240C<br>4.1.2.13 : YKL060C<br>5.3.1.1 : YDR050C<br>1.2.1.12 : YJR009C<br>2.7.2.3 : 639<br>5.4.2.1 : YKL152C<br>4.2.1.11 : YMR323W<br>2.7.1.40 : YAL038W
7	8.08	28	28	0	8	5.3.1.9 : YBR196C<br>2.7.1.11 : YGR240C<br>4.1.2.13 : YKL060C<br>5.3.1.1 : YDR050C<br>1.2.1.12 : YGR192C<br>2.7.2.3 : 639<br>5.4.2.1 : YKL152C<br>4.2.1.11 : YMR323W<br>2.7.1.40 : YAL038W
8	7.99	28	28	0	8	5.3.1.9 : YBR196C<br>2.7.1.11 : YGR240C<br>4.1.2.13 : YKL060C<br>5.3.1.1 : YDR050C<br>1.2.1.12 : YJL052W<br>2.7.2.3 : 639<br>5.4.2.1 : YKL152C<br>4.2.1.11 : YHR174W<br>2.7.1.40 : YAL038W
9	7.98	28	26	2	8	5.3.1.9 : YBR196C<br>2.7.1.11 : YGR240C<br>4.1.2.13 : YKL060C<br>5.3.1.1 : YDR050C<br>1.2.1.12 : YJR009C<br>2.7.2.3 : 639<br>5.4.2.1 : YDL021W<br>4.2.1.11 : YHR174W<br>2.7.1.40 : YAL038W
10	7.96	28	26	2	8	5.3.1.9 : YBR196C<br>2.7.1.11 : YGR240C<br>4.1.2.13 : YKL060C<br>5.3.1.1 : YDR050C<br>1.2.1.12 : YGR192C<br>2.7.2.3 : 639<br>5.4.2.1 : YDL021W<br>4.2.1.11 : YHR174W<br>2.7.1.40 : YAL038W
...<br>...						

Assignments for the pathway "glycolysis" (time series data set). There are 180 possible assignments of genes to reactions(only the 10 highest scoring and the 10 lowest scoring assignments are displayed).

Number	Normalized Score	Number of Pairs	Positive Pairs	Negative Pairs	Zero Pairs	Assignments
171	3.81	28	21	7	8	5.3.1.9 : YBR196C<br>2.7.1.11 : YMR205C<br>4.1.2.13 : YKL060C<br>5.3.1.1 : YDR050C<br>1.2.1.12 : YGR192C<br>2.7.2.3 : 639<br>5.4.2.1 : YOL056W<br>4.2.1.11 : YOR393W<br>2.7.1.40 : YOR347C
172	3.78	28	21	7	8	5.3.1.9 : YBR196C<br>2.7.1.11 : YMR205C<br>4.1.2.13 : YKL060C<br>5.3.1.1 : YDR050C<br>1.2.1.12 : YJR009C<br>2.7.2.3 : 639<br>5.4.2.1 : YOL056W<br>4.2.1.11 : YOR393W<br>2.7.1.40 : YAL038W
173	3.75	28	20	8	8	5.3.1.9 : YBR196C<br>2.7.1.11 : YMR205C<br>4.1.2.13 : YKL060C<br>5.3.1.1 : YDR050C<br>1.2.1.12 : YJR009C<br>2.7.2.3 : 639<br>5.4.2.1 : YOL056W<br>4.2.1.11 : YPL281C<br>2.7.1.40 : YOR347C
174	3.71	28	21	7	8	5.3.1.9 : YBR196C<br>2.7.1.11 : YMR205C<br>4.1.2.13 : YKL060C<br>5.3.1.1 : YDR050C<br>1.2.1.12 : YJR009C<br>2.7.2.3 : 639<br>5.4.2.1 : YOL056W<br>4.2.1.11 : YOR393W<br>2.7.1.40 : YOR347C
175	3.63	28	20	8	8	5.3.1.9 : YBR196C<br>2.7.1.11 : YMR205C<br>4.1.2.13 : YKL060C<br>5.3.1.1 : YDR050C<br>1.2.1.12 : YJL052W<br>2.7.2.3 : 639<br>5.4.2.1 : YOL056W<br>4.2.1.11 : YMR323W<br>2.7.1.40 : YOR347C

Assignments for the pathway ”glycolysis” (time series data set). There are 180 possible assignments of genes to reactions(only the 10 highest scoring and the 10 lowest scoring assignments are displayed).

Number	Normalized Score	Number of Pairs	Positive Pairs	Negative Pairs	Zero Pairs	Assignments
176	3.61	28	19	9	8	5.3.1.9 : YBR196C<br>2.7.1.11 : YGR240C<br>4.1.2.13 : YKL060C<br>5.3.1.1 : YDR050C<br>1.2.1.12 : YJL052W<br>2.7.2.3 : 639<br>5.4.2.1 : YOL056W<br>4.2.1.11 : YOR393W<br>2.7.1.40 : YOR347C
177	3.56	28	20	8	8	5.3.1.9 : YBR196C<br>2.7.1.11 : YMR205C<br>4.1.2.13 : YKL060C<br>5.3.1.1 : YDR050C<br>1.2.1.12 : YJL052W<br>2.7.2.3 : 639<br>5.4.2.1 : YOL056W<br>4.2.1.11 : YPL281C<br>2.7.1.40 : YAL038W
178	3.22	28	19	9	8	5.3.1.9 : YBR196C<br>2.7.1.11 : YMR205C<br>4.1.2.13 : YKL060C<br>5.3.1.1 : YDR050C<br>1.2.1.12 : YJL052W<br>2.7.2.3 : 639<br>5.4.2.1 : YOL056W<br>4.2.1.11 : YPL281C<br>2.7.1.40 : YOR347C
179	3.16	28	20	8	8	5.3.1.9 : YBR196C<br>2.7.1.11 : YMR205C<br>4.1.2.13 : YKL060C<br>5.3.1.1 : YDR050C<br>1.2.1.12 : YJL052W<br>2.7.2.3 : 639<br>5.4.2.1 : YOL056W<br>4.2.1.11 : YOR393W<br>2.7.1.40 : YOR347C
180	3.16	28	20	8	8	5.3.1.9 : YBR196C<br>2.7.1.11 : YMR205C<br>4.1.2.13 : YKL060C<br>5.3.1.1 : YDR050C<br>1.2.1.12 : YJL052W<br>2.7.2.3 : 639<br>5.4.2.1 : YOL056W<br>4.2.1.11 : YOR393W<br>2.7.1.40 : YAL038W

Table 3: **Assignments for the pathway ”glycolysis” (time series data set). There are 180 possible assignments of genes to reactions(only the 10 highest scoring and the 10 lowest scoring assignments are displayed).**

Number	Normalized Score	Number of Pairs	Positive Pairs	Negative Pairs	Zero Pairs	Assignments
1	7.37	10	10	0	0	4.2.1.3 : YLR304C<br>4.1.3.7 : YCR005C<br>4.1.3.1 : YER065C<br>4.1.3.2 : YIR031C<br>1.1.1.37 : YKL085W
2	7.30	10	10	0	0	4.2.1.3 : YLR304C<br>4.1.3.7 : YCR005C<br>4.1.3.1 : YER065C<br>4.1.3.2 : YNL117W<br>1.1.1.37 : YKL085W
3	7.00	10	10	0	0	4.2.1.3 : YLR304C<br>4.1.3.7 : YCR005C<br>4.1.3.1 : YER065C<br>4.1.3.2 : YNL117W<br>1.1.1.37 : YOL126C
4	6.85	10	10	0	0	4.2.1.3 : YLR304C<br>4.1.3.7 : YCR005C<br>4.1.3.1 : YER065C<br>4.1.3.2 : YNL117W<br>1.1.1.37 : YDL078C
5	6.81	10	10	0	0	4.2.1.3 : YLR304C<br>4.1.3.7 : YCR005C<br>4.1.3.1 : YER065C<br>4.1.3.2 : YIR031C<br>1.1.1.37 : YDL078C
6	6.47	10	10	0	0	4.2.1.3 : YLR304C<br>4.1.3.7 : YNR001C<br>4.1.3.1 : YER065C<br>4.1.3.2 : YNL117W<br>1.1.1.37 : YOL126C
7	6.42	10	10	0	0	4.2.1.3 : YLR304C<br>4.1.3.7 : YCR005C<br>4.1.3.1 : YER065C<br>4.1.3.2 : YIR031C<br>1.1.1.37 : YOL126C
8	6.23	10	10	0	0	4.2.1.3 : YLR304C<br>4.1.3.7 : YCR005C<br>4.1.3.1 : YPR006C<br>4.1.3.2 : YNL117W<br>1.1.1.37 : YOL126C
9	6.15	10	10	0	0	4.2.1.3 : YLR304C<br>4.1.3.7 : YCR005C<br>4.1.3.1 : YPR006C<br>4.1.3.2 : YNL117W<br>1.1.1.37 : YKL085W

Assignments for the pathway "glyoxylate cycle" (time series data set). There are 72 possible assignments of genes to reactions.

Number	Normalized Score	Number of Pairs	Positive Pairs	Negative Pairs	Zero Pairs	Assignments
10	6.10	10	10	0	0	4.2.1.3 : YLR304C<br>4.1.3.7 : YPR001W<br>4.1.3.1 : YER065C<br>4.1.3.2 : YNL117W<br>1.1.1.37 : YOL126C
11	6.07	10	10	0	0	4.2.1.3 : YLR304C<br>4.1.3.7 : YNR001C<br>4.1.3.1 : YPR006C<br>4.1.3.2 : YNL117W<br>1.1.1.37 : YOL126C
12	6.06	10	10	0	0	4.2.1.3 : YLR304C<br>4.1.3.7 : YNR001C<br>4.1.3.1 : YER065C<br>4.1.3.2 : YNL117W<br>1.1.1.37 : YKL085W
13	5.90	10	10	0	0	4.2.1.3 : YLR304C<br>4.1.3.7 : YCR005C<br>4.1.3.1 : YPR006C<br>4.1.3.2 : YIR031C<br>1.1.1.37 : YKL085W
14	5.73	10	10	0	0	4.2.1.3 : YLR304C<br>4.1.3.7 : YPR001W<br>4.1.3.1 : YPR006C<br>4.1.3.2 : YNL117W<br>1.1.1.37 : YOL126C
15	5.56	10	9	1	0	4.2.1.3 : YLR304C<br>4.1.3.7 : YPR001W<br>4.1.3.1 : YER065C<br>4.1.3.2 : YNL117W<br>1.1.1.37 : YKL085W
16	5.51	10	10	0	0	4.2.1.3 : YLR304C<br>4.1.3.7 : YNR001C<br>4.1.3.1 : YER065C<br>4.1.3.2 : YNL117W<br>1.1.1.37 : YDL078C
17	5.49	10	9	1	0	4.2.1.3 : YLR304C<br>4.1.3.7 : YPR001W<br>4.1.3.1 : YER065C<br>4.1.3.2 : YNL117W<br>1.1.1.37 : YDL078C
18	5.37	10	10	0	0	4.2.1.3 : YLR304C<br>4.1.3.7 : YNR001C<br>4.1.3.1 : YER065C<br>4.1.3.2 : YIR031C<br>1.1.1.37 : YKL085W

Assignments for the pathway "glyoxylate cycle" (time series data set). There are 72 possible assignments of genes to reactions.

Number	Normalized Score	Number of Pairs	Positive Pairs	Negative Pairs	Zero Pairs	Assignments
19	5.33	10	10	0	0	4.2.1.3 : YLR304C<br>4.1.3.7 : YCR005C<br>4.1.3.1 : YPR006C<br>4.1.3.2 : YIR031C<br>1.1.1.37 : YOL126C
20	5.28	10	10	0	0	4.2.1.3 : YLR304C<br>4.1.3.7 : YNR001C<br>4.1.3.1 : YPR006C<br>4.1.3.2 : YNL117W<br>1.1.1.37 : YKL085W
21	5.15	10	10	0	0	4.2.1.3 : YLR304C<br>4.1.3.7 : YCR005C<br>4.1.3.1 : YPR006C<br>4.1.3.2 : YNL117W<br>1.1.1.37 : YDL078C
22	5.12	10	10	0	0	4.2.1.3 : YLR304C<br>4.1.3.7 : YNR001C<br>4.1.3.1 : YER065C<br>4.1.3.2 : YIR031C<br>1.1.1.37 : YOL126C
23	5.00	10	9	1	0	4.2.1.3 : YLR304C<br>4.1.3.7 : YPR001W<br>4.1.3.1 : YER065C<br>4.1.3.2 : YIR031C<br>1.1.1.37 : YKL085W
24	4.89	10	10	0	0	4.2.1.3 : YLR304C<br>4.1.3.7 : YPR001W<br>4.1.3.1 : YER065C<br>4.1.3.2 : YIR031C<br>1.1.1.37 : YOL126C
25	4.81	10	9	1	0	4.2.1.3 : YLR304C<br>4.1.3.7 : YPR001W<br>4.1.3.1 : YPR006C<br>4.1.3.2 : YNL117W<br>1.1.1.37 : YKL085W
26	4.80	10	9	1	0	4.2.1.3 : YLR304C<br>4.1.3.7 : YPR001W<br>4.1.3.1 : YER065C<br>4.1.3.2 : YIR031C<br>1.1.1.37 : YDL078C
27	4.78	10	10	0	0	4.2.1.3 : YLR304C<br>4.1.3.7 : YCR005C<br>4.1.3.1 : YPR006C<br>4.1.3.2 : YIR031C<br>1.1.1.37 : YDL078C

Assignments for the pathway ”glyoxylate cycle” (time series data set). There are 72 possible assignments of genes to reactions.

Number	Normalized Score	Number of Pairs	Positive Pairs	Negative Pairs	Zero Pairs	Assignments
28	4.70	10	10	0	0	4.2.1.3 : YLR304C<br>4.1.3.7 : YNR001C<br>4.1.3.1 : YER065C<br>4.1.3.2 : YIR031C<br>1.1.1.37 : YDL078C
29	4.41	10	10	0	0	4.2.1.3 : YLR304C<br>4.1.3.7 : YNR001C<br>4.1.3.1 : YPR006C<br>4.1.3.2 : YIR031C<br>1.1.1.37 : YOL126C
30	4.27	10	10	0	0	4.2.1.3 : YLR304C<br>4.1.3.7 : YNR001C<br>4.1.3.1 : YPR006C<br>4.1.3.2 : YIR031C<br>1.1.1.37 : YKL085W
31	4.20	10	10	0	0	4.2.1.3 : YLR304C<br>4.1.3.7 : YPR001W<br>4.1.3.1 : YPR006C<br>4.1.3.2 : YIR031C<br>1.1.1.37 : YOL126C
32	4.18	10	10	0	0	4.2.1.3 : YLR304C<br>4.1.3.7 : YNR001C<br>4.1.3.1 : YPR006C<br>4.1.3.2 : YNL117W<br>1.1.1.37 : YDL078C
33	4.18	10	9	1	0	4.2.1.3 : YLR304C<br>4.1.3.7 : YPR001W<br>4.1.3.1 : YPR006C<br>4.1.3.2 : YNL117W<br>1.1.1.37 : YDL078C
34	3.92	10	9	1	0	4.2.1.3 : YLR304C<br>4.1.3.7 : YPR001W<br>4.1.3.1 : YPR006C<br>4.1.3.2 : YIR031C<br>1.1.1.37 : YKL085W
35	3.56	10	7	3	0	4.2.1.3 : YJL200C<br>4.1.3.7 : YPR001W<br>4.1.3.1 : YPR006C<br>4.1.3.2 : YNL117W<br>1.1.1.37 : YOL126C
36	3.48	10	6	4	0	4.2.1.3 : YJL200C<br>4.1.3.7 : YPR001W<br>4.1.3.1 : YER065C<br>4.1.3.2 : YNL117W<br>1.1.1.37 : YOL126C

Assignments for the pathway ”glyoxylate cycle” (time series data set). There are 72 possible assignments of genes to reactions.

Number	Normalized Score	Number of Pairs	Positive Pairs	Negative Pairs	Zero Pairs	Assignments
37	3.42	10	6	4	0	4.2.1.3 : YJL200C<br>4.1.3.7 : YCR005C<br>4.1.3.1 : YER065C<br>4.1.3.2 : YNL117W<br>1.1.1.37 : YOL126C
38	3.40	10	6	4	0	4.2.1.3 : YJL200C<br>4.1.3.7 : YCR005C<br>4.1.3.1 : YER065C<br>4.1.3.2 : YIR031C<br>1.1.1.37 : YKL085W
39	3.28	10	6	4	0	4.2.1.3 : YJL200C<br>4.1.3.7 : YCR005C<br>4.1.3.1 : YER065C<br>4.1.3.2 : YIR031C<br>1.1.1.37 : YDL078C
40	3.25	10	7	3	0	4.2.1.3 : YJL200C<br>4.1.3.7 : YNR001C<br>4.1.3.1 : YPR006C<br>4.1.3.2 : YNL117W<br>1.1.1.37 : YOL126C
41	3.19	10	6	4	0	4.2.1.3 : YJL200C<br>4.1.3.7 : YNR001C<br>4.1.3.1 : YER065C<br>4.1.3.2 : YNL117W<br>1.1.1.37 : YOL126C
42	3.19	10	6	4	0	4.2.1.3 : YJL200C<br>4.1.3.7 : YCR005C<br>4.1.3.1 : YER065C<br>4.1.3.2 : YNL117W<br>1.1.1.37 : YDL078C
43	3.18	10	6	4	0	4.2.1.3 : YJL200C<br>4.1.3.7 : YCR005C<br>4.1.3.1 : YER065C<br>4.1.3.2 : YNL117W<br>1.1.1.37 : YKL085W
44	3.18	10	9	1	0	4.2.1.3 : YLR304C<br>4.1.3.7 : YPR001W<br>4.1.3.1 : YPR006C<br>4.1.3.2 : YIR031C<br>1.1.1.37 : YDL078C
45	3.11	10	7	3	0	4.2.1.3 : YJL200C<br>4.1.3.7 : YCR005C<br>4.1.3.1 : YPR006C<br>4.1.3.2 : YNL117W<br>1.1.1.37 : YOL126C

Assignments for the pathway ”glyoxylate cycle” (time series data set). There are 72 possible assignments of genes to reactions.

Number	Normalized Score	Number of Pairs	Positive Pairs	Negative Pairs	Zero Pairs	Assignments
46	3.05	10	10	0	0	4.2.1.3 : YLR304C<br>4.1.3.7 : YNR001C<br>4.1.3.1 : YPR006C<br>4.1.3.2 : YIR031C<br>1.1.1.37 : YDL078C
47	2.99	10	6	4	0	4.2.1.3 : YJL200C<br>4.1.3.7 : YCR005C<br>4.1.3.1 : YER065C<br>4.1.3.2 : YIR031C<br>1.1.1.37 : YOL126C
48	2.77	10	5	5	0	4.2.1.3 : YJL200C<br>4.1.3.7 : YPR001W<br>4.1.3.1 : YER065C<br>4.1.3.2 : YNL117W<br>1.1.1.37 : YDL078C
49	2.49	10	7	3	0	4.2.1.3 : YJL200C<br>4.1.3.7 : YCR005C<br>4.1.3.1 : YPR006C<br>4.1.3.2 : YNL117W<br>1.1.1.37 : YKL085W
50	2.41	10	6	4	0	4.2.1.3 : YJL200C<br>4.1.3.7 : YPR001W<br>4.1.3.1 : YER065C<br>4.1.3.2 : YIR031C<br>1.1.1.37 : YOL126C
51	2.40	10	5	5	0	4.2.1.3 : YJL200C<br>4.1.3.7 : YPR001W<br>4.1.3.1 : YER065C<br>4.1.3.2 : YNL117W<br>1.1.1.37 : YKL085W
52	2.38	10	7	3	0	4.2.1.3 : YJL200C<br>4.1.3.7 : YCR005C<br>4.1.3.1 : YPR006C<br>4.1.3.2 : YIR031C<br>1.1.1.37 : YKL085W
53	2.35	10	7	3	0	4.2.1.3 : YJL200C<br>4.1.3.7 : YCR005C<br>4.1.3.1 : YPR006C<br>4.1.3.2 : YIR031C<br>1.1.1.37 : YOL126C
54	2.24	10	6	4	0	4.2.1.3 : YJL200C<br>4.1.3.7 : YNR001C<br>4.1.3.1 : YER065C<br>4.1.3.2 : YNL117W<br>1.1.1.37 : YKL085W

Assignments for the pathway "glyoxylate cycle" (time series data set). There are 72 possible assignments of genes to reactions.

Number	Normalized Score	Number of Pairs	Positive Pairs	Negative Pairs	Zero Pairs	Assignments
55	2.23	10	5	5	0	4.2.1.3 : YJL200C<br>4.1.3.7 : YPR001W<br>4.1.3.1 : YER065C<br>4.1.3.2 : YIR031C<br>1.1.1.37 : YDL078C
56	2.17	10	7	3	0	4.2.1.3 : YJL200C<br>4.1.3.7 : YPR001W<br>4.1.3.1 : YPR006C<br>4.1.3.2 : YIR031C<br>1.1.1.37 : YOL126C
57	2.14	10	6	4	0	4.2.1.3 : YJL200C<br>4.1.3.7 : YNR001C<br>4.1.3.1 : YER065C<br>4.1.3.2 : YNL117W<br>1.1.1.37 : YDL078C
58	2.10	10	6	4	0	4.2.1.3 : YJL200C<br>4.1.3.7 : YPR001W<br>4.1.3.1 : YPR006C<br>4.1.3.2 : YNL117W<br>1.1.1.37 : YKL085W
59	1.99	10	6	4	0	4.2.1.3 : YJL200C<br>4.1.3.7 : YNR001C<br>4.1.3.1 : YER065C<br>4.1.3.2 : YIR031C<br>1.1.1.37 : YOL126C
60	1.98	10	5	5	0	4.2.1.3 : YJL200C<br>4.1.3.7 : YPR001W<br>4.1.3.1 : YER065C<br>4.1.3.2 : YIR031C<br>1.1.1.37 : YKL085W
61	1.94	10	7	3	0	4.2.1.3 : YJL200C<br>4.1.3.7 : YCR005C<br>4.1.3.1 : YPR006C<br>4.1.3.2 : YNL117W<br>1.1.1.37 : YDL078C
62	1.92	10	6	4	0	4.2.1.3 : YJL200C<br>4.1.3.7 : YPR001W<br>4.1.3.1 : YPR006C<br>4.1.3.2 : YNL117W<br>1.1.1.37 : YDL078C
63	1.92	10	7	3	0	4.2.1.3 : YJL200C<br>4.1.3.7 : YNR001C<br>4.1.3.1 : YPR006C<br>4.1.3.2 : YNL117W<br>1.1.1.37 : YKL085W

Assignments for the pathway "glyoxylate cycle" (time series data set). There are 72 possible assignments of genes to reactions.

Number	Normalized Score	Number of Pairs	Positive Pairs	Negative Pairs	Zero Pairs	Assignments
64	1.72	10	7	3	0	4.2.1.3 : YJL200C<br>4.1.3.7 : YNR001C<br>4.1.3.1 : YPR006C<br>4.1.3.2 : YIR031C<br>1.1.1.37 : YOL126C
65	1.71	10	7	3	0	4.2.1.3 : YJL200C<br>4.1.3.7 : YCR005C<br>4.1.3.1 : YPR006C<br>4.1.3.2 : YIR031C<br>1.1.1.37 : YDL078C
66	1.69	10	6	4	0	4.2.1.3 : YJL200C<br>4.1.3.7 : YNR001C<br>4.1.3.1 : YER065C<br>4.1.3.2 : YIR031C<br>1.1.1.37 : YKL085W
67	1.47	10	6	4	0	4.2.1.3 : YJL200C<br>4.1.3.7 : YNR001C<br>4.1.3.1 : YER065C<br>4.1.3.2 : YIR031C<br>1.1.1.37 : YDL078C
68	1.36	10	6	4	0	4.2.1.3 : YJL200C<br>4.1.3.7 : YPR001W<br>4.1.3.1 : YPR006C<br>4.1.3.2 : YIR031C<br>1.1.1.37 : YKL085W
69	1.27	10	7	3	0	4.2.1.3 : YJL200C<br>4.1.3.7 : YNR001C<br>4.1.3.1 : YPR006C<br>4.1.3.2 : YNL117W<br>1.1.1.37 : YDL078C
70	1.06	10	6	4	0	4.2.1.3 : YJL200C<br>4.1.3.7 : YPR001W<br>4.1.3.1 : YPR006C<br>4.1.3.2 : YIR031C<br>1.1.1.37 : YDL078C
71	1.04	10	7	3	0	4.2.1.3 : YJL200C<br>4.1.3.7 : YNR001C<br>4.1.3.1 : YPR006C<br>4.1.3.2 : YIR031C<br>1.1.1.37 : YKL085W
72	0.27	10	7	3	0	4.2.1.3 : YJL200C<br>4.1.3.7 : YNR001C<br>4.1.3.1 : YPR006C<br>4.1.3.2 : YIR031C<br>1.1.1.37 : YDL078C

Table 4: **Assignments for the pathway "glyoxylate cycle" (time series data set). There are 72 possible assignments of genes to reactions.**

Number	Normalized Score	Number of Pairs	Positive Pairs	Negative Pairs	Zero Pairs	Assignments
1	3.49	55	43	12	0	6.3.4.3 : YBR084W<br>6.3.2.17 : YOR241W<br>3.5.4.16 : YGR267C<br>4.1.2.25 : YNL256W<br>2.7.6.3 : YNL256W<br>1.5.1.3 : YOR236W<br>6.3.2.12 : YMR113W<br>2.1.2.1 : YBR263W<br>3.5.4.9 : YBR084W<br>1.5.1.5 : YBR084W<br>2.5.1.15 : YNL256W
2	2.93	55	37	18	0	6.3.4.3 : YBR084W<br>6.3.2.17 : YMR113W<br>3.5.4.16 : YGR267C<br>4.1.2.25 : YNL256W<br>2.7.6.3 : YNL256W<br>1.5.1.3 : YOR236W<br>6.3.2.12 : YMR113W<br>2.1.2.1 : YBR263W<br>3.5.4.9 : YBR084W<br>1.5.1.5 : YBR084W<br>2.5.1.15 : YNL256W
3	2.93	55	37	18	0	6.3.4.3 : YBR084W<br>6.3.2.17 : YKL132C<br>3.5.4.16 : YGR267C<br>4.1.2.25 : YNL256W<br>2.7.6.3 : YNL256W<br>1.5.1.3 : YOR236W<br>6.3.2.12 : YMR113W<br>2.1.2.1 : YBR263W<br>3.5.4.9 : YBR084W<br>1.5.1.5 : YBR084W<br>2.5.1.15 : YNL256W
4	2.56	55	39	16	0	6.3.4.3 : YBR084W<br>6.3.2.17 : YOR241W<br>3.5.4.16 : YGR267C<br>4.1.2.25 : YNL256W<br>2.7.6.3 : YNL256W<br>1.5.1.3 : YOR236W<br>6.3.2.12 : YMR113W<br>2.1.2.1 : YLR058C<br>3.5.4.9 : YBR084W<br>1.5.1.5 : YBR084W<br>2.5.1.15 : YNL256W

Assignments for the pathway "folic acid biosynthesis" (time series data set). There are 48 possible assignments of genes to reactions.

Number	Normalized Score	Number of Pairs	Positive Pairs	Negative Pairs	Zero Pairs	Assignments
5	2.14	55	34	21	0	6.3.4.3 : YBR084W<br>6.3.2.17 : YMR113W<br>3.5.4.16 : YGR267C<br>4.1.2.25 : YNL256W<br>2.7.6.3 : YNL256W<br>1.5.1.3 : YOR236W<br>6.3.2.12 : YMR113W<br>2.1.2.1 : YLR058C<br>3.5.4.9 : YBR084W<br>1.5.1.5 : YBR084W<br>2.5.1.15 : YNL256W
6	2.14	55	39	16	0	6.3.4.3 : YGR204W<br>6.3.2.17 : YOR241W<br>3.5.4.16 : YGR267C<br>4.1.2.25 : YNL256W<br>2.7.6.3 : YNL256W<br>1.5.1.3 : YOR236W<br>6.3.2.12 : YMR113W<br>2.1.2.1 : YBR263W<br>3.5.4.9 : YBR084W<br>1.5.1.5 : YBR084W<br>2.5.1.15 : YNL256W
7	2.14	55	39	16	0	6.3.4.3 : YBR084W<br>6.3.2.17 : YOR241W<br>3.5.4.16 : YGR267C<br>4.1.2.25 : YNL256W<br>2.7.6.3 : YNL256W<br>1.5.1.3 : YOR236W<br>6.3.2.12 : YMR113W<br>2.1.2.1 : YBR263W<br>3.5.4.9 : YGR204W<br>1.5.1.5 : YBR084W<br>2.5.1.15 : YNL256W
8	2.14	55	39	16	0	6.3.4.3 : YBR084W<br>6.3.2.17 : YOR241W<br>3.5.4.16 : YGR267C<br>4.1.2.25 : YNL256W<br>2.7.6.3 : YNL256W<br>1.5.1.3 : YOR236W<br>6.3.2.12 : YMR113W<br>2.1.2.1 : YBR263W<br>3.5.4.9 : YBR084W<br>1.5.1.5 : YGR204W<br>2.5.1.15 : YNL256W

Assignments for the pathway ”folic acid biosynthesis” (time series data set). There are 48 possible assignments of genes to reactions.

Number	Normalized Score	Number of Pairs	Positive Pairs	Negative Pairs	Zero Pairs	Assignments
9	2.08	55	33	22	0	6.3.4.3 : YBR084W<br>6.3.2.17 : YKL132C<br>3.5.4.16 : YGR267C<br>4.1.2.25 : YNL256W<br>2.7.6.3 : YNL256W<br>1.5.1.3 : YOR236W<br>6.3.2.12 : YMR113W<br>2.1.2.1 : YLR058C<br>3.5.4.9 : YBR084W<br>1.5.1.5 : YBR084W<br>2.5.1.15 : YNL256W
10	1.71	55	34	21	0	6.3.4.3 : YGR204W<br>6.3.2.17 : YMR113W<br>3.5.4.16 : YGR267C<br>4.1.2.25 : YNL256W<br>2.7.6.3 : YNL256W<br>1.5.1.3 : YOR236W<br>6.3.2.12 : YMR113W<br>2.1.2.1 : YBR263W<br>3.5.4.9 : YBR084W<br>1.5.1.5 : YBR084W<br>2.5.1.15 : YNL256W
11	1.71	55	34	21	0	6.3.4.3 : YBR084W<br>6.3.2.17 : YMR113W<br>3.5.4.16 : YGR267C<br>4.1.2.25 : YNL256W<br>2.7.6.3 : YNL256W<br>1.5.1.3 : YOR236W<br>6.3.2.12 : YMR113W<br>2.1.2.1 : YBR263W<br>3.5.4.9 : YGR204W<br>1.5.1.5 : YBR084W<br>2.5.1.15 : YNL256W
12	1.71	55	34	21	0	6.3.4.3 : YBR084W<br>6.3.2.17 : YMR113W<br>3.5.4.16 : YGR267C<br>4.1.2.25 : YNL256W<br>2.7.6.3 : YNL256W<br>1.5.1.3 : YOR236W<br>6.3.2.12 : YMR113W<br>2.1.2.1 : YBR263W<br>3.5.4.9 : YBR084W<br>1.5.1.5 : YGR204W<br>2.5.1.15 : YNL256W

Assignments for the pathway ”folic acid biosynthesis” (time series data set). There are 48 possible assignments of genes to reactions.

Number	Normalized Score	Number of Pairs	Positive Pairs	Negative Pairs	Zero Pairs	Assignments
13	1.57	55	33	22	0	6.3.4.3 : YGR204W<br>6.3.2.17 : YKL132C<br>3.5.4.16 : YGR267C<br>4.1.2.25 : YNL256W<br>2.7.6.3 : YNL256W<br>1.5.1.3 : YOR236W<br>6.3.2.12 : YMR113W<br>2.1.2.1 : YBR263W<br>3.5.4.9 : YBR084W<br>1.5.1.5 : YBR084W<br>2.5.1.15 : YNL256W
14	1.57	55	33	22	0	6.3.4.3 : YBR084W<br>6.3.2.17 : YKL132C<br>3.5.4.16 : YGR267C<br>4.1.2.25 : YNL256W<br>2.7.6.3 : YNL256W<br>1.5.1.3 : YOR236W<br>6.3.2.12 : YMR113W<br>2.1.2.1 : YBR263W<br>3.5.4.9 : YGR204W<br>1.5.1.5 : YBR084W<br>2.5.1.15 : YNL256W
15	1.57	55	33	22	0	6.3.4.3 : YBR084W<br>6.3.2.17 : YKL132C<br>3.5.4.16 : YGR267C<br>4.1.2.25 : YNL256W<br>2.7.6.3 : YNL256W<br>1.5.1.3 : YOR236W<br>6.3.2.12 : YMR113W<br>2.1.2.1 : YBR263W<br>3.5.4.9 : YBR084W<br>1.5.1.5 : YGR204W<br>2.5.1.15 : YNL256W
16	1.54	55	36	19	0	6.3.4.3 : YBR084W<br>6.3.2.17 : YOR241W<br>3.5.4.16 : YGR267C<br>4.1.2.25 : YNL256W<br>2.7.6.3 : YNL256W<br>1.5.1.3 : YOR236W<br>6.3.2.12 : YMR113W<br>2.1.2.1 : YLR058C<br>3.5.4.9 : YGR204W<br>1.5.1.5 : YBR084W<br>2.5.1.15 : YNL256W

Assignments for the pathway ”folic acid biosynthesis” (time series data set). There are 48 possible assignments of genes to reactions.

Number	Normalized Score	Number of Pairs	Positive Pairs	Negative Pairs	Zero Pairs	Assignments
17	1.54	55	36	19	0	6.3.4.3 : YBR084W<br>6.3.2.17 : YOR241W<br>3.5.4.16 : YGR267C<br>4.1.2.25 : YNL256W<br>2.7.6.3 : YNL256W<br>1.5.1.3 : YOR236W<br>6.3.2.12 : YMR113W<br>2.1.2.1 : YLR058C<br>3.5.4.9 : YBR084W<br>1.5.1.5 : YGR204W<br>2.5.1.15 : YNL256W
18	1.54	55	36	19	0	6.3.4.3 : YGR204W<br>6.3.2.17 : YOR241W<br>3.5.4.16 : YGR267C<br>4.1.2.25 : YNL256W<br>2.7.6.3 : YNL256W<br>1.5.1.3 : YOR236W<br>6.3.2.12 : YMR113W<br>2.1.2.1 : YLR058C<br>3.5.4.9 : YBR084W<br>1.5.1.5 : YBR084W<br>2.5.1.15 : YNL256W
19	1.26	55	32	23	0	6.3.4.3 : YGR204W<br>6.3.2.17 : YMR113W<br>3.5.4.16 : YGR267C<br>4.1.2.25 : YNL256W<br>2.7.6.3 : YNL256W<br>1.5.1.3 : YOR236W<br>6.3.2.12 : YMR113W<br>2.1.2.1 : YLR058C<br>3.5.4.9 : YBR084W<br>1.5.1.5 : YBR084W<br>2.5.1.15 : YNL256W
20	1.26	55	32	23	0	6.3.4.3 : YBR084W<br>6.3.2.17 : YMR113W<br>3.5.4.16 : YGR267C<br>4.1.2.25 : YNL256W<br>2.7.6.3 : YNL256W<br>1.5.1.3 : YOR236W<br>6.3.2.12 : YMR113W<br>2.1.2.1 : YLR058C<br>3.5.4.9 : YGR204W<br>1.5.1.5 : YBR084W<br>2.5.1.15 : YNL256W

Assignments for the pathway ”folic acid biosynthesis” (time series data set). There are 48 possible assignments of genes to reactions.

Number	Normalized Score	Number of Pairs	Positive Pairs	Negative Pairs	Zero Pairs	Assignments
21	1.26	55	32	23	0	6.3.4.3 : YBR084W<br>6.3.2.17 : YMR113W<br>3.5.4.16 : YGR267C<br>4.1.2.25 : YNL256W<br>2.7.6.3 : YNL256W<br>1.5.1.3 : YOR236W<br>6.3.2.12 : YMR113W<br>2.1.2.1 : YLR058C<br>3.5.4.9 : YBR084W<br>1.5.1.5 : YGR204W<br>2.5.1.15 : YNL256W
22	1.24	55	37	18	0	6.3.4.3 : YBR084W<br>6.3.2.17 : YOR241W<br>3.5.4.16 : YGR267C<br>4.1.2.25 : YNL256W<br>2.7.6.3 : YNL256W<br>1.5.1.3 : YOR236W<br>6.3.2.12 : YMR113W<br>2.1.2.1 : YBR263W<br>3.5.4.9 : YGR204W<br>1.5.1.5 : YGR204W<br>2.5.1.15 : YNL256W
23	1.24	55	37	18	0	6.3.4.3 : YGR204W<br>6.3.2.17 : YOR241W<br>3.5.4.16 : YGR267C<br>4.1.2.25 : YNL256W<br>2.7.6.3 : YNL256W<br>1.5.1.3 : YOR236W<br>6.3.2.12 : YMR113W<br>2.1.2.1 : YBR263W<br>3.5.4.9 : YGR204W<br>1.5.1.5 : YBR084W<br>2.5.1.15 : YNL256W
24	1.24	55	37	18	0	6.3.4.3 : YGR204W<br>6.3.2.17 : YOR241W<br>3.5.4.16 : YGR267C<br>4.1.2.25 : YNL256W<br>2.7.6.3 : YNL256W<br>1.5.1.3 : YOR236W<br>6.3.2.12 : YMR113W<br>2.1.2.1 : YBR263W<br>3.5.4.9 : YBR084W<br>1.5.1.5 : YGR204W<br>2.5.1.15 : YNL256W

Assignments for the pathway ”folic acid biosynthesis” (time series data set). There are 48 possible assignments of genes to reactions.

Number	Normalized Score	Number of Pairs	Positive Pairs	Negative Pairs	Zero Pairs	Assignments
25	1.06	55	30	25	0	6.3.4.3 : YBR084W<br>6.3.2.17 : YKL132C<br>3.5.4.16 : YGR267C<br>4.1.2.25 : YNL256W<br>2.7.6.3 : YNL256W<br>1.5.1.3 : YOR236W<br>6.3.2.12 : YMR113W<br>2.1.2.1 : YLR058C<br>3.5.4.9 : YBR084W<br>1.5.1.5 : YGR204W<br>2.5.1.15 : YNL256W
26	1.06	55	30	25	0	6.3.4.3 : YGR204W<br>6.3.2.17 : YKL132C<br>3.5.4.16 : YGR267C<br>4.1.2.25 : YNL256W<br>2.7.6.3 : YNL256W<br>1.5.1.3 : YOR236W<br>6.3.2.12 : YMR113W<br>2.1.2.1 : YLR058C<br>3.5.4.9 : YBR084W<br>1.5.1.5 : YBR084W<br>2.5.1.15 : YNL256W
27	1.06	55	30	25	0	6.3.4.3 : YBR084W<br>6.3.2.17 : YKL132C<br>3.5.4.16 : YGR267C<br>4.1.2.25 : YNL256W<br>2.7.6.3 : YNL256W<br>1.5.1.3 : YOR236W<br>6.3.2.12 : YMR113W<br>2.1.2.1 : YLR058C<br>3.5.4.9 : YGR204W<br>1.5.1.5 : YBR084W<br>2.5.1.15 : YNL256W
28	0.98	55	35	20	0	6.3.4.3 : YGR204W<br>6.3.2.17 : YOR241W<br>3.5.4.16 : YGR267C<br>4.1.2.25 : YNL256W<br>2.7.6.3 : YNL256W<br>1.5.1.3 : YOR236W<br>6.3.2.12 : YMR113W<br>2.1.2.1 : YLR058C<br>3.5.4.9 : YGR204W<br>1.5.1.5 : YBR084W<br>2.5.1.15 : YNL256W

Assignments for the pathway "folic acid biosynthesis" (time series data set). There are 48 possible assignments of genes to reactions.

Number	Normalized Score	Number of Pairs	Positive Pairs	Negative Pairs	Zero Pairs	Assignments
29	0.98	55	35	20	0	6.3.4.3 : YGR204W<br>6.3.2.17 : YOR241W<br>3.5.4.16 : YGR267C<br>4.1.2.25 : YNL256W<br>2.7.6.3 : YNL256W<br>1.5.1.3 : YOR236W<br>6.3.2.12 : YMR113W<br>2.1.2.1 : YLR058C<br>3.5.4.9 : YBR084W<br>1.5.1.5 : YGR204W<br>2.5.1.15 : YNL256W
30	0.98	55	35	20	0	6.3.4.3 : YBR084W<br>6.3.2.17 : YOR241W<br>3.5.4.16 : YGR267C<br>4.1.2.25 : YNL256W<br>2.7.6.3 : YNL256W<br>1.5.1.3 : YOR236W<br>6.3.2.12 : YMR113W<br>2.1.2.1 : YLR058C<br>3.5.4.9 : YGR204W<br>1.5.1.5 : YGR204W<br>2.5.1.15 : YNL256W
31	0.94	55	33	22	0	6.3.4.3 : YGR204W<br>6.3.2.17 : YMR113W<br>3.5.4.16 : YGR267C<br>4.1.2.25 : YNL256W<br>2.7.6.3 : YNL256W<br>1.5.1.3 : YOR236W<br>6.3.2.12 : YMR113W<br>2.1.2.1 : YBR263W<br>3.5.4.9 : YGR204W<br>1.5.1.5 : YBR084W<br>2.5.1.15 : YNL256W
32	0.94	55	33	22	0	6.3.4.3 : YGR204W<br>6.3.2.17 : YMR113W<br>3.5.4.16 : YGR267C<br>4.1.2.25 : YNL256W<br>2.7.6.3 : YNL256W<br>1.5.1.3 : YOR236W<br>6.3.2.12 : YMR113W<br>2.1.2.1 : YBR263W<br>3.5.4.9 : YBR084W<br>1.5.1.5 : YGR204W<br>2.5.1.15 : YNL256W

Assignments for the pathway ”folic acid biosynthesis” (time series data set). There are 48 possible assignments of genes to reactions.

Number	Normalized Score	Number of Pairs	Positive Pairs	Negative Pairs	Zero Pairs	Assignments
33	0.94	55	33	22	0	6.3.4.3 : YBR084W<br>6.3.2.17 : YMR113W<br>3.5.4.16 : YGR267C<br>4.1.2.25 : YNL256W<br>2.7.6.3 : YNL256W<br>1.5.1.3 : YOR236W<br>6.3.2.12 : YMR113W<br>2.1.2.1 : YBR263W<br>3.5.4.9 : YGR204W<br>1.5.1.5 : YGR204W<br>2.5.1.15 : YNL256W
34	0.87	55	36	19	0	6.3.4.3 : YGR204W<br>6.3.2.17 : YOR241W<br>3.5.4.16 : YGR267C<br>4.1.2.25 : YNL256W<br>2.7.6.3 : YNL256W<br>1.5.1.3 : YOR236W<br>6.3.2.12 : YMR113W<br>2.1.2.1 : YLR058C<br>3.5.4.9 : YGR204W<br>1.5.1.5 : YGR204W<br>2.5.1.15 : YNL256W
35	0.86	55	34	21	0	6.3.4.3 : YGR204W<br>6.3.2.17 : YMR113W<br>3.5.4.16 : YGR267C<br>4.1.2.25 : YNL256W<br>2.7.6.3 : YNL256W<br>1.5.1.3 : YOR236W<br>6.3.2.12 : YMR113W<br>2.1.2.1 : YLR058C<br>3.5.4.9 : YGR204W<br>1.5.1.5 : YGR204W<br>2.5.1.15 : YNL256W
36	0.83	55	32	23	0	6.3.4.3 : YGR204W<br>6.3.2.17 : YMR113W<br>3.5.4.16 : YGR267C<br>4.1.2.25 : YNL256W<br>2.7.6.3 : YNL256W<br>1.5.1.3 : YOR236W<br>6.3.2.12 : YMR113W<br>2.1.2.1 : YLR058C<br>3.5.4.9 : YGR204W<br>1.5.1.5 : YBR084W<br>2.5.1.15 : YNL256W

Assignments for the pathway "folic acid biosynthesis" (time series data set). There are 48 possible assignments of genes to reactions.

Number	Normalized Score	Number of Pairs	Positive Pairs	Negative Pairs	Zero Pairs	Assignments
37	0.83	55	32	23	0	6.3.4.3 : YGR204W
						6.3.2.17 : YMR113W
						3.5.4.16 : YGR267C
						4.1.2.25 : YNL256W
						2.7.6.3 : YNL256W
						1.5.1.3 : YOR236W
						6.3.2.12 : YMR113W
						2.1.2.1 : YLR058C
						3.5.4.9 : YBR084W
						1.5.1.5 : YGR204W
						2.5.1.15 : YNL256W
38	0.83	55	32	23	0	6.3.4.3 : YBR084W
						6.3.2.17 : YMR113W
						3.5.4.16 : YGR267C
						4.1.2.25 : YNL256W
						2.7.6.3 : YNL256W
						1.5.1.3 : YOR236W
						6.3.2.12 : YMR113W
						2.1.2.1 : YLR058C
						3.5.4.9 : YGR204W
						1.5.1.5 : YGR204W
						2.5.1.15 : YNL256W
39	0.79	55	37	18	0	6.3.4.3 : YGR204W
						6.3.2.17 : YOR241W
						3.5.4.16 : YGR267C
						4.1.2.25 : YNL256W
						2.7.6.3 : YNL256W
						1.5.1.3 : YOR236W
						6.3.2.12 : YMR113W
						2.1.2.1 : YBR263W
						3.5.4.9 : YGR204W
						1.5.1.5 : YGR204W
						2.5.1.15 : YNL256W
40	0.67	55	31	24	0	6.3.4.3 : YGR204W
						6.3.2.17 : YKL132C
						3.5.4.16 : YGR267C
						4.1.2.25 : YNL256W
						2.7.6.3 : YNL256W
						1.5.1.3 : YOR236W
						6.3.2.12 : YMR113W
						2.1.2.1 : YBR263W
						3.5.4.9 : YGR204W
						1.5.1.5 : YBR084W
						2.5.1.15 : YNL256W

Assignments for the pathway ”folic acid biosynthesis” (time series data set). There are 48 possible assignments of genes to reactions.

Number	Normalized Score	Number of Pairs	Positive Pairs	Negative Pairs	Zero Pairs	Assignments
41	0.67	55	31	24	0	6.3.4.3 : YBR084W<br>6.3.2.17 : YKL132C<br>3.5.4.16 : YGR267C<br>4.1.2.25 : YNL256W<br>2.7.6.3 : YNL256W<br>1.5.1.3 : YOR236W<br>6.3.2.12 : YMR113W<br>2.1.2.1 : YBR263W<br>3.5.4.9 : YGR204W<br>1.5.1.5 : YGR204W<br>2.5.1.15 : YNL256W
42	0.67	55	31	24	0	6.3.4.3 : YGR204W<br>6.3.2.17 : YKL132C<br>3.5.4.16 : YGR267C<br>4.1.2.25 : YNL256W<br>2.7.6.3 : YNL256W<br>1.5.1.3 : YOR236W<br>6.3.2.12 : YMR113W<br>2.1.2.1 : YBR263W<br>3.5.4.9 : YBR084W<br>1.5.1.5 : YGR204W<br>2.5.1.15 : YNL256W
43	0.63	55	34	21	0	6.3.4.3 : YGR204W<br>6.3.2.17 : YMR113W<br>3.5.4.16 : YGR267C<br>4.1.2.25 : YNL256W<br>2.7.6.3 : YNL256W<br>1.5.1.3 : YOR236W<br>6.3.2.12 : YMR113W<br>2.1.2.1 : YBR263W<br>3.5.4.9 : YGR204W<br>1.5.1.5 : YGR204W<br>2.5.1.15 : YNL256W
44	0.50	55	29	26	0	6.3.4.3 : YGR204W<br>6.3.2.17 : YKL132C<br>3.5.4.16 : YGR267C<br>4.1.2.25 : YNL256W<br>2.7.6.3 : YNL256W<br>1.5.1.3 : YOR236W<br>6.3.2.12 : YMR113W<br>2.1.2.1 : YLR058C<br>3.5.4.9 : YGR204W<br>1.5.1.5 : YBR084W<br>2.5.1.15 : YNL256W

Assignments for the pathway ”folic acid biosynthesis” (time series data set). There are 48 possible assignments of genes to reactions.

Number	Normalized Score	Number of Pairs	Positive Pairs	Negative Pairs	Zero Pairs	Assignments
45	0.50	55	29	26	0	6.3.4.3 : YGR204W<br>6.3.2.17 : YKL132C<br>3.5.4.16 : YGR267C<br>4.1.2.25 : YNL256W<br>2.7.6.3 : YNL256W<br>1.5.1.3 : YOR236W<br>6.3.2.12 : YMR113W<br>2.1.2.1 : YLR058C<br>3.5.4.9 : YBR084W<br>1.5.1.5 : YGR204W<br>2.5.1.15 : YNL256W
46	0.50	55	29	26	0	6.3.4.3 : YBR084W<br>6.3.2.17 : YKL132C<br>3.5.4.16 : YGR267C<br>4.1.2.25 : YNL256W<br>2.7.6.3 : YNL256W<br>1.5.1.3 : YOR236W<br>6.3.2.12 : YMR113W<br>2.1.2.1 : YLR058C<br>3.5.4.9 : YGR204W<br>1.5.1.5 : YGR204W<br>2.5.1.15 : YNL256W
47	0.40	55	30	25	0	6.3.4.3 : YGR204W<br>6.3.2.17 : YKL132C<br>3.5.4.16 : YGR267C<br>4.1.2.25 : YNL256W<br>2.7.6.3 : YNL256W<br>1.5.1.3 : YOR236W<br>6.3.2.12 : YMR113W<br>2.1.2.1 : YLR058C<br>3.5.4.9 : YGR204W<br>1.5.1.5 : YGR204W<br>2.5.1.15 : YNL256W
48	0.23	55	31	24	0	6.3.4.3 : YGR204W<br>6.3.2.17 : YKL132C<br>3.5.4.16 : YGR267C<br>4.1.2.25 : YNL256W<br>2.7.6.3 : YNL256W<br>1.5.1.3 : YOR236W<br>6.3.2.12 : YMR113W<br>2.1.2.1 : YBR263W<br>3.5.4.9 : YGR204W<br>1.5.1.5 : YGR204W<br>2.5.1.15 : YNL256W

Table 5: **Assignments for the pathway ”folic acid biosynthesis” (time series data set). There are 48 possible assignments of genes to reactions.**

Number	Normalized Score	Number of Pairs	Positive Pairs	Negative Pairs	Zero Pairs	Assignments
1	2.43	90	63	27	1	6.3.5.2 : YMR217W
						1.1.1.205 : YAR073W
						4.3.2.2 : YLR359W
						6.3.4.4 : YNL220W
						3.5.4.10 : YLR028C
						2.1.2.3 : YLR028C
						4.3.2.2 : YLR359W
						6.3.2.6 : YAR015W
						4.1.1.21 : YOR128C
						6.3.3.1 : YGL234W
						6.3.5.3 : YGR061C
						2.1.2.2 : YDR408C
						6.3.4.13 : YGL234W
						2.4.2.14 : YMR300C
2	2.39	90	61	29	1	6.3.5.2 : YMR217W
						1.1.1.205 : YLR432W
						4.3.2.2 : YLR359W
						6.3.4.4 : YNL220W
						3.5.4.10 : YLR028C
						2.1.2.3 : YLR028C
						4.3.2.2 : YLR359W
						6.3.2.6 : YAR015W
						4.1.1.21 : YOR128C
						6.3.3.1 : YGL234W
						6.3.5.3 : YGR061C
						2.1.2.2 : YDR408C
						6.3.4.13 : YGL234W
						2.4.2.14 : YMR300C
3	2.37	77	54	23	14	6.3.5.2 : YMR217W
						1.1.1.205 : 4401
						4.3.2.2 : YLR359W
						6.3.4.4 : YNL220W
						3.5.4.10 : YLR028C
						2.1.2.3 : YLR028C
						4.3.2.2 : YLR359W
						6.3.2.6 : YAR015W
						4.1.1.21 : YOR128C
						6.3.3.1 : YGL234W
						6.3.5.3 : YGR061C
						2.1.2.2 : YDR408C
						6.3.4.13 : YGL234W
						2.4.2.14 : YMR300C

Assignments for the pathway "purine biosynthesis 2" (time series data set). There are 16 possible assignments of genes to reactions.

Number	Normalized Score	Number of Pairs	Positive Pairs	Negative Pairs	Zero Pairs	Assignments
4	2.34	90	60	30	1	6.3.5.2 : YMR217W<br>1.1.1.205 : YHR216W<br>4.3.2.2 : YLR359W<br>6.3.4.4 : YNL220W<br>3.5.4.10 : YLR028C<br>2.1.2.3 : YLR028C<br>4.3.2.2 : YLR359W<br>6.3.2.6 : YAR015W<br>4.1.1.21 : YOR128C<br>6.3.3.1 : YGL234W<br>6.3.5.3 : YGR061C<br>2.1.2.2 : YDR408C<br>6.3.4.13 : YGL234W<br>2.4.2.14 : YMR300C
5	2.17	90	63	27	1	6.3.5.2 : YMR217W<br>1.1.1.205 : YAR073W<br>4.3.2.2 : YLR359W<br>6.3.4.4 : YNL220W<br>3.5.4.10 : YMR120C<br>2.1.2.3 : YMR120C<br>4.3.2.2 : YLR359W<br>6.3.2.6 : YAR015W<br>4.1.1.21 : YOR128C<br>6.3.3.1 : YGL234W<br>6.3.5.3 : YGR061C<br>2.1.2.2 : YDR408C<br>6.3.4.13 : YGL234W<br>2.4.2.14 : YMR300C
6	2.11	90	63	27	1	6.3.5.2 : YMR217W<br>1.1.1.205 : YAR073W<br>4.3.2.2 : YLR359W<br>6.3.4.4 : YNL220W<br>3.5.4.10 : YMR120C<br>2.1.2.3 : YLR028C<br>4.3.2.2 : YLR359W<br>6.3.2.6 : YAR015W<br>4.1.1.21 : YOR128C<br>6.3.3.1 : YGL234W<br>6.3.5.3 : YGR061C<br>2.1.2.2 : YDR408C<br>6.3.4.13 : YGL234W<br>2.4.2.14 : YMR300C

Assignments for the pathway "purine biosynthesis 2" (time series data set). There are 16 possible assignments of genes to reactions.

Number	Normalized Score	Number of Pairs	Positive Pairs	Negative Pairs	Zero Pairs	Assignments
7	2.11	90	63	27	1	6.3.5.2 : YMR217W<br>1.1.1.205 : YAR073W<br>4.3.2.2 : YLR359W<br>6.3.4.4 : YNL220W<br>3.5.4.10 : YLR028C<br>2.1.2.3 : YMR120C<br>4.3.2.2 : YLR359W<br>6.3.2.6 : YAR015W<br>4.1.1.21 : YOR128C<br>6.3.3.1 : YGL234W<br>6.3.5.3 : YGR061C<br>2.1.2.2 : YDR408C<br>6.3.4.13 : YGL234W<br>2.4.2.14 : YMR300C
8	2.10	90	61	29	1	6.3.5.2 : YMR217W<br>1.1.1.205 : YLR432W<br>4.3.2.2 : YLR359W<br>6.3.4.4 : YNL220W<br>3.5.4.10 : YMR120C<br>2.1.2.3 : YMR120C<br>4.3.2.2 : YLR359W<br>6.3.2.6 : YAR015W<br>4.1.1.21 : YOR128C<br>6.3.3.1 : YGL234W<br>6.3.5.3 : YGR061C<br>2.1.2.2 : YDR408C<br>6.3.4.13 : YGL234W<br>2.4.2.14 : YMR300C
9	2.09	77	54	23	14	6.3.5.2 : YMR217W<br>1.1.1.205 : 4401<br>4.3.2.2 : YLR359W<br>6.3.4.4 : YNL220W<br>3.5.4.10 : YMR120C<br>2.1.2.3 : YMR120C<br>4.3.2.2 : YLR359W<br>6.3.2.6 : YAR015W<br>4.1.1.21 : YOR128C<br>6.3.3.1 : YGL234W<br>6.3.5.3 : YGR061C<br>2.1.2.2 : YDR408C<br>6.3.4.13 : YGL234W<br>2.4.2.14 : YMR300C

Assignments for the pathway ”purine biosynthesis 2” (time series data set). There are 16 possible assignments of genes to reactions.

Number	Normalized Score	Number of Pairs	Positive Pairs	Negative Pairs	Zero Pairs	Assignments
10	2.09	90	60	30	1	6.3.5.2 : YMR217W<br>1.1.1.205 : YHR216W<br>4.3.2.2 : YLR359W<br>6.3.4.4 : YNL220W<br>3.5.4.10 : YMR120C<br>2.1.2.3 : YMR120C<br>4.3.2.2 : YLR359W<br>6.3.2.6 : YAR015W<br>4.1.1.21 : YOR128C<br>6.3.3.1 : YGL234W<br>6.3.5.3 : YGR061C<br>2.1.2.2 : YDR408C<br>6.3.4.13 : YGL234W<br>2.4.2.14 : YMR300C
11	2.05	90	61	29	1	6.3.5.2 : YMR217W<br>1.1.1.205 : YLR432W<br>4.3.2.2 : YLR359W<br>6.3.4.4 : YNL220W<br>3.5.4.10 : YMR120C<br>2.1.2.3 : YLR028C<br>4.3.2.2 : YLR359W<br>6.3.2.6 : YAR015W<br>4.1.1.21 : YOR128C<br>6.3.3.1 : YGL234W<br>6.3.5.3 : YGR061C<br>2.1.2.2 : YDR408C<br>6.3.4.13 : YGL234W<br>2.4.2.14 : YMR300C
12	2.05	90	61	29	1	6.3.5.2 : YMR217W<br>1.1.1.205 : YLR432W<br>4.3.2.2 : YLR359W<br>6.3.4.4 : YNL220W<br>3.5.4.10 : YLR028C<br>2.1.2.3 : YMR120C<br>4.3.2.2 : YLR359W<br>6.3.2.6 : YAR015W<br>4.1.1.21 : YOR128C<br>6.3.3.1 : YGL234W<br>6.3.5.3 : YGR061C<br>2.1.2.2 : YDR408C<br>6.3.4.13 : YGL234W<br>2.4.2.14 : YMR300C

Assignments for the pathway "purine biosynthesis 2" (time series data set). There are 16 possible assignments of genes to reactions.

Number	Normalized Score	Number of Pairs	Positive Pairs	Negative Pairs	Zero Pairs	Assignments
13	2.03	90	60	30	1	6.3.5.2 : YMR217W<br>1.1.1.205 : YHR216W<br>4.3.2.2 : YLR359W<br>6.3.4.4 : YNL220W<br>3.5.4.10 : YMR120C<br>2.1.2.3 : YLR028C<br>4.3.2.2 : YLR359W<br>6.3.2.6 : YAR015W<br>4.1.1.21 : YOR128C<br>6.3.3.1 : YGL234W<br>6.3.5.3 : YGR061C<br>2.1.2.2 : YDR408C<br>6.3.4.13 : YGL234W<br>2.4.2.14 : YMR300C
14	2.03	90	60	30	1	6.3.5.2 : YMR217W<br>1.1.1.205 : YHR216W<br>4.3.2.2 : YLR359W<br>6.3.4.4 : YNL220W<br>3.5.4.10 : YLR028C<br>2.1.2.3 : YMR120C<br>4.3.2.2 : YLR359W<br>6.3.2.6 : YAR015W<br>4.1.1.21 : YOR128C<br>6.3.3.1 : YGL234W<br>6.3.5.3 : YGR061C<br>2.1.2.2 : YDR408C<br>6.3.4.13 : YGL234W<br>2.4.2.14 : YMR300C
15	2.01	77	54	23	14	6.3.5.2 : YMR217W<br>1.1.1.205 : 4401<br>4.3.2.2 : YLR359W<br>6.3.4.4 : YNL220W<br>3.5.4.10 : YMR120C<br>2.1.2.3 : YLR028C<br>4.3.2.2 : YLR359W<br>6.3.2.6 : YAR015W<br>4.1.1.21 : YOR128C<br>6.3.3.1 : YGL234W<br>6.3.5.3 : YGR061C<br>2.1.2.2 : YDR408C<br>6.3.4.13 : YGL234W<br>2.4.2.14 : YMR300C

Assignments for the pathway ”purine biosynthesis 2” (time series data set). There are 16 possible assignments of genes to reactions.

Number	Normalized Score	Number of Pairs	Positive Pairs	Negative Pairs	Zero Pairs	Assignments
16	2.01	77	54	23	14	6.3.5.2 : YMR217W<br>1.1.1.205 : 4401<br>4.3.2.2 : YLR359W<br>6.3.4.4 : YNL220W<br>3.5.4.10 : YLR028C<br>2.1.2.3 : YMR120C<br>4.3.2.2 : YLR359W<br>6.3.2.6 : YAR015W<br>4.1.1.21 : YOR128C<br>6.3.3.1 : YGL234W<br>6.3.5.3 : YGR061C<br>2.1.2.2 : YDR408C<br>6.3.4.13 : YGL234W<br>2.4.2.14 : YMR300C

Table 6: **Assignments for the pathway "purine biosynthesis 2" (time series data set). There are 16 possible assignments of genes to reactions.**

Number	Normalized Score	Number of Pairs	Positive Pairs	Negative Pairs	Zero Pairs	Assignments
1	0.14	43	18	25	2	2.7.4.6 : YKL067W<br>2.4.2.10 : YML106W<br>6.3.4.2 : YBL039C<br>2.7.4.6 : YKL067W<br>4.1.1.23 : YEL021W<br>2.4.2.10 : YML106W<br>1.3.3.1 : YKL216W<br>3.5.2.3 : YLR420W<br>2.1.3.2 : YJL130C<br>6.3.5.5 : YJL130C
2	-0.07	43	16	27	2	2.7.4.6 : YKL067W<br>2.4.2.10 : YML106W<br>6.3.4.2 : YBL039C<br>2.7.4.6 : YKL067W<br>4.1.1.23 : YEL021W<br>2.4.2.10 : YML106W<br>1.3.3.1 : YKL216W<br>3.5.2.3 : YLR420W<br>2.1.3.2 : YJL130C<br>6.3.5.5 : YOR303W

Assignments for the pathway "de novo biosynthesis of pyrimidine ribonucleotides" (time series data set). There are 12 possible assignments of genes to reactions.

Number	Normalized Score	Number of Pairs	Positive Pairs	Negative Pairs	Zero Pairs	Assignments
3	-0.13	43	19	24	2	2.7.4.6 : YKL067W<br>2.4.2.10 : YML106W<br>6.3.4.2 : YBL039C<br>2.7.4.6 : YKL067W<br>4.1.1.23 : YEL021W<br>2.4.2.10 : YML106W<br>1.3.3.1 : YKL216W<br>3.5.2.3 : YLR420W<br>2.1.3.2 : YJL130C<br>6.3.5.5 : YJR109C
4	-0.40	43	15	28	2	2.7.4.6 : YKL067W<br>2.4.2.10 : YML106W<br>6.3.4.2 : YJR103W<br>2.7.4.6 : YKL067W<br>4.1.1.23 : YEL021W<br>2.4.2.10 : YML106W<br>1.3.3.1 : YKL216W<br>3.5.2.3 : YLR420W<br>2.1.3.2 : YJL130C<br>6.3.5.5 : YJL130C
5	-0.41	43	15	28	2	2.7.4.6 : YKL067W<br>2.4.2.10 : YMR271C<br>6.3.4.2 : YJR103W<br>2.7.4.6 : YKL067W<br>4.1.1.23 : YEL021W<br>2.4.2.10 : YMR271C<br>1.3.3.1 : YKL216W<br>3.5.2.3 : YLR420W<br>2.1.3.2 : YJL130C<br>6.3.5.5 : YOR303W
6	-0.42	43	16	27	2	2.7.4.6 : YKL067W<br>2.4.2.10 : YMR271C<br>6.3.4.2 : YJR103W<br>2.7.4.6 : YKL067W<br>4.1.1.23 : YEL021W<br>2.4.2.10 : YMR271C<br>1.3.3.1 : YKL216W<br>3.5.2.3 : YLR420W<br>2.1.3.2 : YJL130C<br>6.3.5.5 : YJR109C

Assignments for the pathway ”de novo biosynthesis of pyrimidine ribonucleotides” (time series data set). There are 12 possible assignments of genes to reactions.

Number	Normalized Score	Number of Pairs	Positive Pairs	Negative Pairs	Zero Pairs	Assignments
7	-0.43	43	15	28	2	2.7.4.6 : YKL067W<br>2.4.2.10 : YMR271C<br>6.3.4.2 : YJR103W<br>2.7.4.6 : YKL067W<br>4.1.1.23 : YEL021W<br>2.4.2.10 : YMR271C<br>1.3.3.1 : YKL216W<br>3.5.2.3 : YLR420W<br>2.1.3.2 : YJL130C<br>6.3.5.5 : YJL130C
8	-0.56	43	16	27	2	2.7.4.6 : YKL067W<br>2.4.2.10 : YML106W<br>6.3.4.2 : YJR103W<br>2.7.4.6 : YKL067W<br>4.1.1.23 : YEL021W<br>2.4.2.10 : YML106W<br>1.3.3.1 : YKL216W<br>3.5.2.3 : YLR420W<br>2.1.3.2 : YJL130C<br>6.3.5.5 : YJR109C
9	-0.58	43	16	27	2	2.7.4.6 : YKL067W<br>2.4.2.10 : YMR271C<br>6.3.4.2 : YBL039C<br>2.7.4.6 : YKL067W<br>4.1.1.23 : YEL021W<br>2.4.2.10 : YMR271C<br>1.3.3.1 : YKL216W<br>3.5.2.3 : YLR420W<br>2.1.3.2 : YJL130C<br>6.3.5.5 : YOR303W
10	-0.60	43	16	27	2	2.7.4.6 : YKL067W<br>2.4.2.10 : YMR271C<br>6.3.4.2 : YBL039C<br>2.7.4.6 : YKL067W<br>4.1.1.23 : YEL021W<br>2.4.2.10 : YMR271C<br>1.3.3.1 : YKL216W<br>3.5.2.3 : YLR420W<br>2.1.3.2 : YJL130C<br>6.3.5.5 : YJL130C

Assignments for the pathway "de novo biosynthesis of pyrimidine ribonucleotides" (time series data set). There are 12 possible assignments of genes to reactions.

Number	Normalized Score	Number of Pairs	Positive Pairs	Negative Pairs	Zero Pairs	Assignments
11	-0.60	43	13	30	2	2.7.4.6 : YKL067W<br>2.4.2.10 : YML106W<br>6.3.4.2 : YJR103W<br>2.7.4.6 : YKL067W<br>4.1.1.23 : YEL021W<br>2.4.2.10 : YML106W<br>1.3.3.1 : YKL216W<br>3.5.2.3 : YLR420W<br>2.1.3.2 : YJL130C<br>6.3.5.5 : YOR303W
12	-0.69	43	17	26	2	2.7.4.6 : YKL067W<br>2.4.2.10 : YMR271C<br>6.3.4.2 : YBL039C<br>2.7.4.6 : YKL067W<br>4.1.1.23 : YEL021W<br>2.4.2.10 : YMR271C<br>1.3.3.1 : YKL216W<br>3.5.2.3 : YLR420W<br>2.1.3.2 : YJL130C<br>6.3.5.5 : YJR109C

Table 7: **Assignments for the pathway ”de novo biosynthesis of pyrimidine ribonucleotides” (time series data set). There are 12 possible assignments of genes to reactions.**

Number	Normalized Score	Number of Pairs	Positive Pairs	Negative Pairs	Zero Pairs	Assignments
1	10.32	10	10	0	0	4.2.1.16 : YER086W<br>4.1.3.18 : YMR108W<br>1.1.1.86 : YLR355C<br>2.6.1.42 : YHR208W<br>4.2.1.9 : YJR016C
2	10.14	6	6	0	4	4.2.1.16 : 570<br>4.1.3.18 : YMR108W<br>1.1.1.86 : YLR355C<br>2.6.1.42 : YHR208W<br>4.2.1.9 : YJR016C
3	9.54	10	10	0	0	4.2.1.16 : YER086W<br>4.1.3.18 : YCL009C<br>1.1.1.86 : YLR355C<br>2.6.1.42 : YHR208W<br>4.2.1.9 : YJR016C
4	9.20	6	6	0	4	4.2.1.16 : 570<br>4.1.3.18 : YCL009C<br>1.1.1.86 : YLR355C<br>2.6.1.42 : YHR208W<br>4.2.1.9 : YJR016C

Assignments for the pathway ”isoleucine biosynthesis I” (time series data set). There are 12 possible assignments of genes to reactions.

Number	Normalized Score	Number of Pairs	Positive Pairs	Negative Pairs	Zero Pairs	Assignments
5	7.37	10	10	0	0	4.2.1.16 : YER086W<br>4.1.3.18 : YMR108W<br>1.1.1.86 : YLR355C<br>2.6.1.42 : YJR148W<br>4.2.1.9 : YJR016C
6	6.49	10	8	2	0	4.2.1.16 : YKL218C<br>4.1.3.18 : YMR108W<br>1.1.1.86 : YLR355C<br>2.6.1.42 : YHR208W<br>4.2.1.9 : YJR016C
7	6.40	6	6	0	4	4.2.1.16 : 570<br>4.1.3.18 : YMR108W<br>1.1.1.86 : YLR355C<br>2.6.1.42 : YJR148W<br>4.2.1.9 : YJR016C
8	6.30	10	9	1	0	4.2.1.16 : YER086W<br>4.1.3.18 : YCL009C<br>1.1.1.86 : YLR355C<br>2.6.1.42 : YJR148W<br>4.2.1.9 : YJR016C
9	5.00	10	7	3	0	4.2.1.16 : YKL218C<br>4.1.3.18 : YCL009C<br>1.1.1.86 : YLR355C<br>2.6.1.42 : YHR208W<br>4.2.1.9 : YJR016C
10	4.99	6	5	1	4	4.2.1.16 : 570<br>4.1.3.18 : YCL009C<br>1.1.1.86 : YLR355C<br>2.6.1.42 : YJR148W<br>4.2.1.9 : YJR016C
11	4.78	10	8	2	0	4.2.1.16 : YKL218C<br>4.1.3.18 : YMR108W<br>1.1.1.86 : YLR355C<br>2.6.1.42 : YJR148W<br>4.2.1.9 : YJR016C
12	3.00	10	6	4	0	4.2.1.16 : YKL218C<br>4.1.3.18 : YCL009C<br>1.1.1.86 : YLR355C<br>2.6.1.42 : YJR148W<br>4.2.1.9 : YJR016C

Table 8: **Assignments for the pathway "isoleucine biosynthesis I" (time series data set). There are 12 possible assignments of genes to reactions.**

Number	Normalized Score	Number of Pairs	Positive Pairs	Negative Pairs	Zero Pairs	Assignments
1	4.82	10	9	1	0	1.1.1.44 : YHR183W<br>5.3.1.6 : YOR095C<br>5.1.3.1 : YJL121C<br>2.2.1.1 : YPR074C<br>2.2.1.2 : YLR354C
2	2.55	10	5	5	0	1.1.1.44 : YGR256W<br>5.3.1.6 : YOR095C<br>5.1.3.1 : YJL121C<br>2.2.1.1 : YPR074C<br>2.2.1.2 : YLR354C
3	2.08	10	4	6	0	1.1.1.44 : YGR256W<br>5.3.1.6 : YOR095C<br>5.1.3.1 : YJL121C<br>2.2.1.1 : YBR117C<br>2.2.1.2 : YGR043C
4	1.91	10	6	4	0	1.1.1.44 : YHR183W<br>5.3.1.6 : YOR095C<br>5.1.3.1 : YJL121C<br>2.2.1.1 : YPR074C<br>2.2.1.2 : YGR043C
5	1.81	10	6	4	0	1.1.1.44 : YHR183W<br>5.3.1.6 : YOR095C<br>5.1.3.1 : YJL121C<br>2.2.1.1 : YBR117C<br>2.2.1.2 : YLR354C
6	1.61	10	4	6	0	1.1.1.44 : YGR256W<br>5.3.1.6 : YOR095C<br>5.1.3.1 : YJL121C<br>2.2.1.1 : YPR074C<br>2.2.1.2 : YGR043C
7	1.07	10	4	6	0	1.1.1.44 : YGR256W<br>5.3.1.6 : YOR095C<br>5.1.3.1 : YJL121C<br>2.2.1.1 : YBR117C<br>2.2.1.2 : YLR354C
8	0.84	10	4	6	0	1.1.1.44 : YHR183W<br>5.3.1.6 : YOR095C<br>5.1.3.1 : YJL121C<br>2.2.1.1 : YBR117C<br>2.2.1.2 : YGR043C

Table 9: **Assignments for the pathway "non-oxidative branch of the pentose phosphate pathway" (time series data set). There are 8 possible assignments of genes to reactions.**

Number	Normalized Score	Number of Pairs	Positive Pairs	Negative Pairs	Zero Pairs	Assignments
1	8.80	6	6	0	0	3.1.3.12 : YDR074W<br>2.4.1.15 : YBR126C<br>2.7.7.9 : YKL035W<br>5.4.2.2 : YMR105C
2	8.62	6	6	0	0	3.1.3.12 : YDR074W<br>2.4.1.15 : YML100W<br>2.7.7.9 : YKL035W<br>5.4.2.2 : YMR105C
3	5.66	6	5	1	0	3.1.3.12 : YDR074W<br>2.4.1.15 : YML100W<br>2.7.7.9 : YKL035W<br>5.4.2.2 : YKL127W
4	5.54	6	5	1	0	3.1.3.12 : YDR074W<br>2.4.1.15 : YBR126C<br>2.7.7.9 : YKL035W<br>5.4.2.2 : YKL127W
5	3.47	6	3	3	0	3.1.3.12 : YDR074W<br>2.4.1.15 : YBR126C<br>2.7.7.9 : YHL012W<br>5.4.2.2 : YMR105C
6	3.37	6	3	3	0	3.1.3.12 : YDR074W<br>2.4.1.15 : YML100W<br>2.7.7.9 : YHL012W<br>5.4.2.2 : YMR105C
7	0.47	6	2	4	0	3.1.3.12 : YDR074W<br>2.4.1.15 : YML100W<br>2.7.7.9 : YHL012W<br>5.4.2.2 : YKL127W
8	0.27	6	2	4	0	3.1.3.12 : YDR074W<br>2.4.1.15 : YBR126C<br>2.7.7.9 : YHL012W<br>5.4.2.2 : YKL127W

Table 10: **Assignments for the pathway "trehalose anabolism" (time series data set). There are 8 possible assignments of genes to reactions.**

Number	Normalized Score	Number of Pairs	Positive Pairs	Negative Pairs	Zero Pairs	Assignments
1	4.78	3	3	0	0	1.1.1.49 : YNL241C<br>1.1.1.44 : YGR256W<br>3.1.1.31 : YGR248W
2	3.11	3	3	0	0	1.1.1.49 : YNL241C<br>1.1.1.44 : YGR256W<br>3.1.1.31 : YNR034W
3	2.89	3	3	0	0	1.1.1.49 : YNL241C<br>1.1.1.44 : YHR183W<br>3.1.1.31 : YHR163W

Assignments for the pathway "oxidative branch of the pentose phosphate pathway" (time series data set). There are 6 possible assignments of genes to reactions.

Number	Normalized Score	Number of Pairs	Positive Pairs	Negative Pairs	Zero Pairs	Assignments
4	2.18	3	2	1	0	1.1.1.49 : YNL241C<br>1.1.1.44 : YHR183W<br>3.1.1.31 : YNR034W
5	1.45	3	2	1	0	1.1.1.49 : YNL241C<br>1.1.1.44 : YHR183W<br>3.1.1.31 : YGR248W
6	1.07	3	3	0	0	1.1.1.49 : YNL241C<br>1.1.1.44 : YGR256W<br>3.1.1.31 : YHR163W

Table 11: **Assignments for the pathway "oxidative branch of the pentose phosphate pathway" (time series data set). There are 6 possible assignments of genes to reactions.**

Number	Normalized Score	Number of Pairs	Positive Pairs	Negative Pairs	Zero Pairs	Assignments
1	-1.32	3	1	2	0	3.6.1.1 : YBR011C<br>5.1.3.2 : YBR019C<br>2.7.7.9 : YKL035W
2	-1.80	3	1	2	0	3.6.1.1 : YBR011C<br>5.1.3.2 : YBR019C<br>2.7.7.9 : YHL012W
3	-1.91	3	1	2	0	3.6.1.1 : YMR267W<br>5.1.3.2 : YBR019C<br>2.7.7.9 : YHL012W
4	-2.04	3	1	2	0	3.6.1.1 : YMR267W<br>5.1.3.2 : YBR019C<br>2.7.7.9 : YKL035W

Table 12: **Assignments for the pathway "UDP-glucose conversion" (time series data set). There are 4 possible assignments of genes to reactions.**

Number	Normalized Score	Number of Pairs	Positive Pairs	Negative Pairs	Zero Pairs	Assignments
1	4.85	1	1	0	0	2.6.1.1 : YLR027C<br>6.4.1.1 : YBR218C
2	1.88	1	1	0	0	2.6.1.1 : YKL106W<br>6.4.1.1 : YGL062W
3	0.86	1	1	0	0	2.6.1.1 : YLR027C<br>6.4.1.1 : YGL062W
4	0.75	1	1	0	0	2.6.1.1 : YKL106W<br>6.4.1.1 : YBR218C

Table 13: **Assignments for the pathway "aspartate biosynthesis II" (time series data set). There are 4 possible assignments of genes to reactions.**

Number	Normalized Score	Number of Pairs	Positive Pairs	Negative Pairs	Zero Pairs	Assignments
1	8.80	1	1	0	0	6.3.5.4 : YPR145W<br>2.6.1.1 : YLR027C
2	8.56	1	1	0	0	6.3.5.4 : YGR124W<br>2.6.1.1 : YLR027C
3	-2.24	1	0	1	0	6.3.5.4 : YPR145W<br>2.6.1.1 : YKL106W
4	-4.88	1	0	1	0	6.3.5.4 : YGR124W<br>2.6.1.1 : YKL106W

Table 14: **Assignments for the pathway "asparagine biosynthesis I" (time series data set). There are 4 possible assignments of genes to reactions.**

Number	Normalized Score	Number of Pairs	Positive Pairs	Negative Pairs	Zero Pairs	Assignments
1	7.91	6	6	0	0	2.7.7.9 : YHL012W<br>5.1.3.2 : YBR019C<br>2.7.7.12 : YBR018C<br>2.7.1.6 : YBR020W
2	7.11	6	6	0	0	2.7.7.9 : YHL012W<br>5.1.3.2 : YBR019C<br>2.7.7.12 : YBR018C<br>2.7.1.6 : YDR009W
3	4.94	6	4	2	0	2.7.7.9 : YKL035W<br>5.1.3.2 : YBR019C<br>2.7.7.12 : YBR018C<br>2.7.1.6 : YBR020W
4	4.62	6	4	2	0	2.7.7.9 : YKL035W<br>5.1.3.2 : YBR019C<br>2.7.7.12 : YBR018C<br>2.7.1.6 : YDR009W

Table 15: **Assignments for the pathway "galactose metabolism" (time series data set). There are 4 possible assignments of genes to reactions.**

Number	Normalized Score	Number of Pairs	Positive Pairs	Negative Pairs	Zero Pairs	Assignments
1	10.14	6	6	0	0	4.1.3.18 : YMR108W<br>1.1.1.86 : YLR355C<br>2.6.1.42 : YHR208W<br>4.2.1.9 : YJR016C
2	9.20	6	6	0	0	4.1.3.18 : YCL009C<br>1.1.1.86 : YLR355C<br>2.6.1.42 : YHR208W<br>4.2.1.9 : YJR016C

Assignments for the pathway "valine biosynthesis" (time series data set). There are 4 possible assignments of genes to reactions.

Number	Normalized Score	Number of Pairs	Positive Pairs	Negative Pairs	Zero Pairs	Assignments
3	6.40	6	6	0	0	4.1.3.18 : YMR108W<br>1.1.1.86 : YLR355C<br>2.6.1.42 : YJR148W<br>4.2.1.9 : YJR016C
4	4.99	6	5	1	0	4.1.3.18 : YCL009C<br>1.1.1.86 : YLR355C<br>2.6.1.42 : YJR148W<br>4.2.1.9 : YJR016C

Table 16: **Assignments for the pathway "valine biosynthesis" (time series data set). There are 4 possible assignments of genes to reactions.**

Number	Normalized Score	Number of Pairs	Positive Pairs	Negative Pairs	Zero Pairs	Assignments
1	10.08	3	3	0	3	4.1.3.12 : YNL104C<br>4.2.1.33 : YGL009C<br>2.6.1.42 : YHR208W<br>1.1.1.85 : 612
2	9.55	3	3	0	3	4.1.3.12 : YOR108W<br>4.2.1.33 : YGL009C<br>2.6.1.42 : YHR208W<br>1.1.1.85 : 612
3	4.31	3	3	0	3	4.1.3.12 : YNL104C<br>4.2.1.33 : YGL009C<br>2.6.1.42 : YJR148W<br>1.1.1.85 : 612
4	4.01	3	3	0	3	4.1.3.12 : YOR108W<br>4.2.1.33 : YGL009C<br>2.6.1.42 : YJR148W<br>1.1.1.85 : 612

Table 17: **Assignments for the pathway "leucine biosynthesis" (time series data set). There are 4 possible assignments of genes to reactions.**

Number	Normalized Score	Number of Pairs	Positive Pairs	Negative Pairs	Zero Pairs	Assignments
1	5.93	1	1	0	0	1.11.1.6 : YGR088W<br>1.15.1.1 : YHR008C
2	5.66	1	1	0	0	1.11.1.6 : YGR088W<br>1.15.1.1 : YJR104C
3	2.29	1	1	0	0	1.11.1.6 : YDR256C<br>1.15.1.1 : YJR104C
4	-0.45	1	0	1	0	1.11.1.6 : YDR256C<br>1.15.1.1 : YHR008C

Table 18: **Assignments for the pathway "removal of superoxide radicals" (time series data set). There are 4 possible assignments of genes to reactions.**

Number	Normalized Score	Number of Pairs	Positive Pairs	Negative Pairs	Zero Pairs	Assignments
1	4.55	34	30	4	2	2.3.1.35 : YMR062C<br>6.3.5.5 : YJR109C<br>2.3.1.1 : YJL071W<br>2.7.2.8 : YER069W<br>1.2.1.38 : YER069W<br>2.6.1.11 : YOL140W<br>2.1.3.3 : YJL088W<br>4.3.2.1 : YHR018C<br>6.3.4.5 : YOL058W
2	3.54	34	30	4	2	2.3.1.35 : YMR062C<br>6.3.5.5 : YOR303W<br>2.3.1.1 : YJL071W<br>2.7.2.8 : YER069W<br>1.2.1.38 : YER069W<br>2.6.1.11 : YOL140W<br>2.1.3.3 : YJL088W<br>4.3.2.1 : YHR018C<br>6.3.4.5 : YOL058W
3	2.69	34	25	9	2	2.3.1.35 : YMR062C<br>6.3.5.5 : YJL130C<br>2.3.1.1 : YJL071W<br>2.7.2.8 : YER069W<br>1.2.1.38 : YER069W<br>2.6.1.11 : YOL140W<br>2.1.3.3 : YJL088W<br>4.3.2.1 : YHR018C<br>6.3.4.5 : YOL058W

Table 19: **Assignments for the pathway "arginine biosynthesis, Bacillus subtilis" (time series data set). There are 3 possible assignments of genes to reactions.**

Number	Normalized Score	Number of Pairs	Positive Pairs	Negative Pairs	Zero Pairs	Assignments
1	4.13	10	9	1	0	4.1.3.27 : YER090W<br>2.4.2.18 : YDR354W<br>5.3.1.24 : YDR007W<br>4.1.1.48 : YKL211C<br>4.2.1.20 : YGL026C
2	3.99	10	8	2	0	4.1.3.27 : YKL211C<br>2.4.2.18 : YDR354W<br>5.3.1.24 : YDR007W<br>4.1.1.48 : YKL211C<br>4.2.1.20 : YGL026C

Table 20: **Assignments for the pathway "tryptophan biosynthesis" (time series data set). There are 2 possible assignments of genes to reactions.**

Number	Normalized Score	Number of Pairs	Positive Pairs	Negative Pairs	Zero Pairs	Assignments
1	11.03	1	1	0	0	2.2.1.1 : YPR074C<br>5.1.3.1 : YJL121C
2	-0.73	1	0	1	0	2.2.1.1 : YBR117C<br>5.1.3.1 : YJL121C

Table 21: **Assignments for the pathway "pentose phosphate pathway, Mycoplasma pneumoniae" (time series data set). There are 2 possible assignments of genes to reactions.**

Number	Normalized Score	Number of Pairs	Positive Pairs	Negative Pairs	Zero Pairs	Assignments
1	6.43	1	1	0	2	1.2.4.1 : YER178W<br>2.3.1.12 : YNL071W<br>1.8.1.4 : 1891
2	4.33	1	1	0	2	1.2.4.1 : YBR221C<br>2.3.1.12 : YNL071W<br>1.8.1.4 : 1891

Table 22: **Assignments for the pathway "pyruvate dehydrogenase" (time series data set). There are 2 possible assignments of genes to reactions.**

Number	Normalized Score	Number of Pairs	Positive Pairs	Negative Pairs	Zero Pairs	Assignments
1	7.73	1	1	0	0	2.1.2.10 : YDR019C<br>1.4.4.2 : YMR189W
2	7.73	1	1	0	0	2.1.2.10 : YMR189W<br>1.4.4.2 : YMR189W

Table 23: **Assignments for the pathway "glycine degradation III" (time series data set). There are 2 possible assignments of genes to reactions.**

Number	Normalized Score	Number of Pairs	Positive Pairs	Negative Pairs	Zero Pairs	Assignments
1	4.19	6	6	0	0	4.2.1.22 : YGR155W<br>3.1.3.3 : YGR208W<br>2.6.1.52 : YOR184W<br>1.1.1.95 : YIL074C
2	0.94	6	4	2	0	4.2.1.22 : YGR155W<br>3.1.3.3 : YGR208W<br>2.6.1.52 : YOR184W<br>1.1.1.95 : YER081W

Table 24: **Assignments for the pathway "cysteine biosynthesis II" (time series data set). There are 2 possible assignments of genes to reactions.**

Number	Normalized Score	Number of Pairs	Positive Pairs	Negative Pairs	Zero Pairs	Assignments
1	7.59	1	1	0	0	3.1.2.6 : YDR272W<br>4.4.1.5 : YML004C
2	0.98	1	1	0	0	3.1.2.6 : YOR040W<br>4.4.1.5 : YML004C

Table 25: **Assignments for the pathway "methylglyoxal degradation" (time series data set). There are 2 possible assignments of genes to reactions.**

Number	Normalized Score	Number of Pairs	Positive Pairs	Negative Pairs	Zero Pairs	Assignments
1	-0.91	1	0	1	0	2.1.2.1 : YBR263W<br>3.1.3.3 : YGR208W
2	-3.60	1	0	1	0	2.1.2.1 : YLR058C<br>3.1.3.3 : YGR208W

Table 26: **Assignments for the pathway "glycine biosynthesis I" (time series data set). There are 2 possible assignments of genes to reactions.**

Number	Normalized Score	Number of Pairs	Positive Pairs	Negative Pairs	Zero Pairs	Assignments
1	2.35	3	2	1	0	4.4.1.8 : YGL184C<br>4.4.1.1 : YAL012W<br>4.2.1.22 : YGR155W
2	2.01	1	1	0	2	4.4.1.8 : 1971<br>4.4.1.1 : YAL012W<br>4.2.1.22 : YGR155W

Table 27: **Assignments for the pathway "homocysteine and cysteine interconversion" (time series data set). There are 2 possible assignments of genes to reactions.**

Number	Normalized Score	Number of Pairs	Positive Pairs	Negative Pairs	Zero Pairs	Assignments
1	-0.24	3	1	2	0	2.5.1.22 : YLR146C<br>2.5.1.16 : YPR069C<br>4.1.1.17 : YKL184W
2	-2.48	3	0	3	0	2.5.1.22 : YLR146C<br>2.5.1.16 : YLR146C<br>4.1.1.17 : YKL184W

Table 28: **Assignments for the pathway "ornithine spermine biosynthesis" (time series data set). There are 2 possible assignments of genes to reactions.**

Number	Normalized Score	Number of Pairs	Positive Pairs	Negative Pairs	Zero Pairs	Assignments
1	4.08	1	1	0	0	1.4.1.4 : YOR375C<br>1.4.1.13 : YDL171C
2	-4.88	1	0	1	0	1.4.1.4 : YAL062W<br>1.4.1.13 : YDL171C

Table 29: **Assignments for the pathway "glutamate biosynthesis I" (time series data set). There are 2 possible assignments of genes to reactions.**

Number	Normalized Score	Number of Pairs	Positive Pairs	Negative Pairs	Zero Pairs	Assignments
1	9.24	21	21	0	0	4.1.2.15 : YBR249C<br>4.6.1.3 : YDR127W<br>4.2.1.10 : YDR127W<br>1.1.1.25 : YDR127W<br>2.7.1.71 : YDR127W<br>2.5.1.19 : YDR127W<br>4.6.1.4 : YGL148W
2	8.19	21	20	1	0	4.1.2.15 : YDR035W<br>4.6.1.3 : YDR127W<br>4.2.1.10 : YDR127W<br>1.1.1.25 : YDR127W<br>2.7.1.71 : YDR127W<br>2.5.1.19 : YDR127W<br>4.6.1.4 : YGL148W

Table 30: **Assignments for the pathway "chorismate biosynthesis" (time series data set). There are 2 possible assignments of genes to reactions.**

Number	Normalized Score	Number of Pairs	Positive Pairs	Negative Pairs	Zero Pairs	Assignments
1	3.58	3	3	0	0	1.1.1.95 : YIL074C<br>3.1.3.3 : YGR208W<br>2.6.1.52 : YOR184W
2	-0.58	3	2	1	0	1.1.1.95 : YER081W<br>3.1.3.3 : YGR208W<br>2.6.1.52 : YOR184W

Table 31: **Assignments for the pathway "serine biosynthesis" (time series data set). There are 2 possible assignments of genes to reactions.**

Number	Normalized Score	Number of Pairs	Positive Pairs	Negative Pairs	Zero Pairs	Assignments
1	-2.09	3	1	2	0	5.4.99.5 : YPR060C<br>4.2.1.51 : YNL316C<br>2.6.1.57 : YHR137W
2	-2.80	3	1	2	0	5.4.99.5 : YPR060C<br>4.2.1.51 : YNL316C<br>2.6.1.57 : YGL202W

Table 32: **Assignments for the pathway "phenylalanine biosynthesis I" (time series data set). There are 2 possible assignments of genes to reactions.**

Number	Normalized Score	Number of Pairs	Positive Pairs	Negative Pairs	Zero Pairs	Assignments
1	-0.53	3	1	2	0	5.4.99.5 : YPR060C<br>1.3.1.13 : YBR166C<br>2.6.1.57 : YHR137W
2	-0.58	3	2	1	0	5.4.99.5 : YPR060C<br>1.3.1.13 : YBR166C<br>2.6.1.57 : YGL202W

Table 33: **Assignments for the pathway "tyrosine biosynthesis I" (time series data set). There are 2 possible assignments of genes to reactions.**

Number	Normalized Score	Number of Pairs	Positive Pairs	Negative Pairs	Zero Pairs	Assignments
1	8.06	1	1	0	2	2.2.1.1 : YPR074C<br>5.3.1.6 : YOR095C<br>2.7.1.15 : 667
2	-1.74	1	0	1	2	2.2.1.1 : YBR117C<br>5.3.1.6 : YOR095C<br>2.7.1.15 : 667

Table 34: **Assignments for the pathway "ribose degradation" (time series data set). There are 2 possible assignments of genes to reactions.**

Number	Normalized Score	Number of Pairs	Positive Pairs	Negative Pairs	Zero Pairs	Assignments
1	10.02	1	1	0	0	3.1.3.12 : YDR074W<br>2.4.1.15 : YBR126C
2	9.65	1	1	0	0	3.1.3.12 : YDR074W<br>2.4.1.15 : YML100W

Table 35: **Assignments for the pathway "trehalose biosynthesis" (time series data set). There are 2 possible assignments of genes to reactions.**

Number	Normalized Score	Number of Pairs	Positive Pairs	Negative Pairs	Zero Pairs	Assignments
1	10.45	1	1	0	0	2.5.1.6 : YDR502C<br>2.1.1.14 : YER091C
2	7.34	1	1	0	0	2.5.1.6 : YLR180W<br>2.1.1.14 : YER091C

Table 36: **Assignments for the pathway "methionine and ¡I¿S¡/I¿-adenosylmethionine synthesis" (time series data set). There are 2 possible assignments of genes to reactions.**

Number	Normalized Score	Number of Pairs	Positive Pairs	Negative Pairs	Zero Pairs	Assignments
1	9.64	3	3	0	0	1.2.1.16 : YBR006W<br>4.1.1.15 : YMR250W<br>2.6.1.19 : YGR019W

Table 37: **Assignments for the pathway "glutamate degradation I" (time series data set). There are 1 possible assignments of genes to reactions.**

Number	Normalized Score	Number of Pairs	Positive Pairs	Negative Pairs	Zero Pairs	Assignments
1	2.27	3	1	2	3	2.3.1.47 : N/A<br>2.6.1.62 : YNR058W<br>2.8.1.6 : YGR286C<br>6.3.3.3 : YNR057C

Table 38: **Assignments for the pathway "biotin biosynthesis I" (time series data set). There are 1 possible assignments of genes to reactions.**

Number	Normalized Score	Number of Pairs	Positive Pairs	Negative Pairs	Zero Pairs	Assignments
1	1.86	8	6	2	2	2.3.1.35 : YMR062C<br>2.6.1.11 : YOL140W<br>1.2.1.38 : YER069W<br>2.7.2.8 : YER069W<br>2.3.1.1 : YJL071W

Table 39: **Assignments for the pathway "glutamate degradation VIII" (time series data set). There are 1 possible assignments of genes to reactions.**

Number	Normalized Score	Number of Pairs	Positive Pairs	Negative Pairs	Zero Pairs	Assignments
1	0.87	1	1	0	0	4.2.99.2 : YCR053W<br>2.7.1.39 : YHR025W

Table 40: **Assignments for the pathway "threonine biosynthesis from homoserine" (time series data set). There are 1 possible assignments of genes to reactions.**

Number	Normalized Score	Number of Pairs	Positive Pairs	Negative Pairs	Zero Pairs	Assignments
1	2.01	1	1	0	0	4.4.1.1 : YAL012W<br>4.2.1.22 : YGR155W

Table 41: **Assignments for the pathway "homocysteine degradation I" (time series data set). There are 1 possible assignments of genes to reactions.**

Number	Normalized Score	Number of Pairs	Positive Pairs	Negative Pairs	Zero Pairs	Assignments
1	7.33	1	1	0	0	2.3.1.31 : YNL277W<br>2.1.1.14 : YER091C

Table 42: **Assignments for the pathway "homoserine methionine biosynthesis" (time series data set). There are 1 possible assignments of genes to reactions.**

Number	Normalized Score	Number of Pairs	Positive Pairs	Negative Pairs	Zero Pairs	Assignments
1	8.43	3	3	0	0	2.7.2.11 : YDR300C<br>1.2.1.41 : YOR323C<br>1.5.1.2 : YER023W

Table 43: **Assignments for the pathway "proline biosynthesis I" (time series data set). There are 1 possible assignments of genes to reactions.**

Number	Normalized Score	Number of Pairs	Positive Pairs	Negative Pairs	Zero Pairs	Assignments
1	11.02	1	1	0	2	1.8.1.2 : 1944<br>2.7.1.25 : YKL001C<br>2.7.7.4 : YJR010W

Table 44: **Assignments for the pathway "sulfate assimilation 2" (time series data set). There are 1 possible assignments of genes to reactions.**

Number	Normalized Score	Number of Pairs	Positive Pairs	Negative Pairs	Zero Pairs	Assignments
1	4.22	1	1	0	0	2.6.1.16 : YKL104C<br>2.7.7.23 : YDL103C

Table 45: **Assignments for the pathway "UDP-N-acetylglucosamine biosynthesis" (time series data set). There are 1 possible assignments of genes to reactions.**

Number	Normalized Score	Number of Pairs	Positive Pairs	Negative Pairs	Zero Pairs	Assignments
1	9.58	3	3	0	0	2.1.3.3 : YJL088W<br>4.3.2.1 : YHR018C<br>6.3.4.5 : YOL058W

Table 46: **Assignments for the pathway "arginine biosynthesis I" (time series data set). There are 1 possible assignments of genes to reactions.**

Number	Normalized Score	Number of Pairs	Positive Pairs	Negative Pairs	Zero Pairs	Assignments
1	2.48	12	10	2	24	2.4.2.17 : YER055C<br>3.5.4.19 : 601<br>3.6.1.31 : 601<br>5.3.1.16 : YIL020C<br>4.2.1.19 : YOR202W<br>2.6.1.9 : YIL116W<br>3.1.3.15 : 1939<br>1.1.1.23 : 601<br>1.1.1.23 : 601

Table 47: **Assignments for the pathway "histidine biosynthesis I" (time series data set). There are 1 possible assignments of genes to reactions.**

Number	Normalized Score	Number of Pairs	Positive Pairs	Negative Pairs	Zero Pairs	Assignments
1	4.00	3	3	0	0	2.6.1.13 : YLR438W<br>1.5.1.12 : YHR037W<br>3.5.3.1 : YPL111W

Table 48: **Assignments for the pathway "arginine degradation I" (time series data set). There are 1 possible assignments of genes to reactions.**

Number	Normalized Score	Number of Pairs	Positive Pairs	Negative Pairs	Zero Pairs	Assignments
1	1.14	3	2	1	0	1.1.1.3 : YJR139C<br>1.2.1.11 : YDR158W<br>2.7.2.4 : YER052C

Table 49: **Assignments for the pathway "homoserine biosynthesis" (time series data set). There are 1 possible assignments of genes to reactions.**

Number	Normalized Score	Number of Pairs	Positive Pairs	Negative Pairs	Zero Pairs	Assignments
1	3.84	3	3	0	0	3.5.3.1 : YPL111W<br>2.6.1.13 : YLR438W<br>1.5.1.2 : YER023W

Table 50: **Assignments for the pathway "arginine proline degradation" (time series data set). There are 1 possible assignments of genes to reactions.**

Number	Normalized Score	Number of Pairs	Positive Pairs	Negative Pairs	Zero Pairs	Assignments
1	4.03	1	1	0	0	6.3.2.3 : YOL049W<br>6.3.2.2 : YJL101C

Table 51: **Assignments for the pathway "glutathione biosynthesis" (time series data set). There are 1 possible assignments of genes to reactions.**

Number	Normalized Score	Number of Pairs	Positive Pairs	Negative Pairs	Zero Pairs	Assignments
1	-2.46	1	0	1	0	1.13.11.11 : YJR078W<br>3.5.1.9 : YJL060W

Table 52: **Assignments for the pathway "tryptophan kynurenine degradation" (time series data set). There are 1 possible assignments of genes to reactions.**
