# Supplementary material for: Automation of gene assignments to metabolic pathways using high-throughput expression data
Source: BMC Bioinformatics. 2005 Aug 31;6:217. doi: 10.1186/1471-2105-6-217 (PMC1239907; doi:10.1186/1471-2105-6-217)
Supplement: Additional File 2 — Assignments of genes to pathways with the rosetta dataset. For each pathway we list the 10 highest scoring and the 10 lowest scoring assignments (or all assignments, if the number of assignments is 100 or less). [file 1471-2105-6-217-S2.pdf]

Number	Normalized Score	Number of Pairs	Positive Pairs	Negative Pairs	Zero Pairs	Assignments
1	9.51	153	144	9	37	6.1.1.14 : YBR121C<br>6.1.1.21 : YPR033C<br>6.1.1.6 : YDR037W<br>6.1.1.10 : YGR264C<br>6.1.1.20 : 1887<br>6.1.1.15 : YHR020W<br>6.1.1.11 : YDR023W<br>6.1.1.3 : 2924<br>6.1.1.2 : YOL097C<br>6.1.1.1 : YGR185C<br>6.1.1.12 : YLL018C<br>6.1.1.22 : YHR019C<br>6.1.1.7 : YOR335C<br>6.1.1.9 : YGR094W<br>6.1.1.4 : YPL160W<br>6.1.1.5 : YBL076C<br>6.1.1.17 : YGL245W<br>6.1.1.18 : YOR168W<br>6.1.1.16 : YNL247W<br>6.1.1.19 : YDR341C
2	9.44	171	162	9	19	6.1.1.14 : YBR121C<br>6.1.1.21 : YPR033C<br>6.1.1.6 : YDR037W<br>6.1.1.10 : YGR264C<br>6.1.1.20 : YLR060W<br>6.1.1.15 : YHR020W<br>6.1.1.11 : YDR023W<br>6.1.1.3 : 2924<br>6.1.1.2 : YOL097C<br>6.1.1.1 : YGR185C<br>6.1.1.12 : YLL018C<br>6.1.1.22 : YHR019C<br>6.1.1.7 : YOR335C<br>6.1.1.9 : YGR094W<br>6.1.1.4 : YPL160W<br>6.1.1.5 : YBL076C<br>6.1.1.17 : YGL245W<br>6.1.1.18 : YOR168W<br>6.1.1.16 : YNL247W<br>6.1.1.19 : YDR341C

Assignments for the pathway "tRNA charging pathway" (rosetta data set). There are 49152 possible assignments of genes to reactions(only the 10 highest scoring and the 10 lowest scoring assignments are displayed).

Number	Normalized Score	Number of Pairs	Positive Pairs	Negative Pairs	Zero Pairs	Assignments
3	8.57	153	136	17	37	6.1.1.14 : YBR121C<br>6.1.1.21 : YPR033C<br>6.1.1.6 : YDR037W<br>6.1.1.10 : YGR264C<br>6.1.1.20 : 1887<br>6.1.1.15 : YHR020W<br>6.1.1.11 : YHR011W<br>6.1.1.3 : 2924<br>6.1.1.2 : YOL097C<br>6.1.1.1 : YGR185C<br>6.1.1.12 : YLL018C<br>6.1.1.22 : YHR019C<br>6.1.1.7 : YOR335C<br>6.1.1.9 : YGR094W<br>6.1.1.4 : YPL160W<br>6.1.1.5 : YBL076C<br>6.1.1.17 : YGL245W<br>6.1.1.18 : YOR168W<br>6.1.1.16 : YNL247W<br>6.1.1.19 : YDR341C
4	8.57	171	154	17	19	6.1.1.14 : YBR121C<br>6.1.1.21 : YPR033C<br>6.1.1.6 : YDR037W<br>6.1.1.10 : YGR264C<br>6.1.1.20 : YLR060W<br>6.1.1.15 : YHR020W<br>6.1.1.11 : YHR011W<br>6.1.1.3 : 2924<br>6.1.1.2 : YOL097C<br>6.1.1.1 : YGR185C<br>6.1.1.12 : YLL018C<br>6.1.1.22 : YHR019C<br>6.1.1.7 : YOR335C<br>6.1.1.9 : YGR094W<br>6.1.1.4 : YPL160W<br>6.1.1.5 : YBL076C<br>6.1.1.17 : YGL245W<br>6.1.1.18 : YOR168W<br>6.1.1.16 : YNL247W<br>6.1.1.19 : YDR341C

Assignments for the pathway "tRNA charging pathway" (rosetta data set). There are 49152 possible assignments of genes to reactions(only the 10 highest scoring and the 10 lowest scoring assignments are displayed).

Number	Normalized Score	Number of Pairs	Positive Pairs	Negative Pairs	Zero Pairs	Assignments
5	8.42	171	150	21	19	6.1.1.14 : YPR081C<br>6.1.1.21 : YPR033C<br>6.1.1.6 : YDR037W<br>6.1.1.10 : YGR264C<br>6.1.1.20 : YLR060W<br>6.1.1.15 : YHR020W<br>6.1.1.11 : YDR023W<br>6.1.1.3 : 2924<br>6.1.1.2 : YOL097C<br>6.1.1.1 : YGR185C<br>6.1.1.12 : YLL018C<br>6.1.1.22 : YHR019C<br>6.1.1.7 : YOR335C<br>6.1.1.9 : YGR094W<br>6.1.1.4 : YPL160W<br>6.1.1.5 : YBL076C<br>6.1.1.17 : YGL245W<br>6.1.1.18 : YOR168W<br>6.1.1.16 : YNL247W<br>6.1.1.19 : YDR341C
6	8.41	153	133	20	37	6.1.1.14 : YPR081C<br>6.1.1.21 : YPR033C<br>6.1.1.6 : YDR037W<br>6.1.1.10 : YGR264C<br>6.1.1.20 : 1887<br>6.1.1.15 : YHR020W<br>6.1.1.11 : YDR023W<br>6.1.1.3 : 2924<br>6.1.1.2 : YOL097C<br>6.1.1.1 : YGR185C<br>6.1.1.12 : YLL018C<br>6.1.1.22 : YHR019C<br>6.1.1.7 : YOR335C<br>6.1.1.9 : YGR094W<br>6.1.1.4 : YPL160W<br>6.1.1.5 : YBL076C<br>6.1.1.17 : YGL245W<br>6.1.1.18 : YOR168W<br>6.1.1.16 : YNL247W<br>6.1.1.19 : YDR341C

Assignments for the pathway "tRNA charging pathway" (rosetta data set). There are 49152 possible assignments of genes to reactions(only the 10 highest scoring and the 10 lowest scoring assignments are displayed).

Number	Normalized Score	Number of Pairs	Positive Pairs	Negative Pairs	Zero Pairs	Assignments
7	8.41	171	153	18	19	6.1.1.14 : YBR121C<br>6.1.1.21 : YPR033C<br>6.1.1.6 : YDR037W<br>6.1.1.10 : YGR264C<br>6.1.1.20 : YLR060W<br>6.1.1.15 : YHR020W<br>6.1.1.11 : YDR023W<br>6.1.1.3 : 2924<br>6.1.1.2 : YOL097C<br>6.1.1.1 : YGR185C<br>6.1.1.12 : YLL018C<br>6.1.1.22 : YHR019C<br>6.1.1.7 : YOR335C<br>6.1.1.9 : YGR094W<br>6.1.1.4 : YPL160W<br>6.1.1.5 : YBL076C<br>6.1.1.17 : YGL245W<br>6.1.1.18 : YOR168W<br>6.1.1.16 : YNL247W<br>6.1.1.19 : YHR091C
8	8.39	171	150	21	19	6.1.1.14 : YBR121C<br>6.1.1.21 : YPR033C<br>6.1.1.6 : YDR037W<br>6.1.1.10 : YGR264C<br>6.1.1.20 : YLR060W<br>6.1.1.15 : YHR020W<br>6.1.1.11 : YDR023W<br>6.1.1.3 : 2924<br>6.1.1.2 : YOL097C<br>6.1.1.1 : YGR185C<br>6.1.1.12 : YLL018C<br>6.1.1.22 : YHR019C<br>6.1.1.7 : YOR335C<br>6.1.1.9 : YGR094W<br>6.1.1.4 : YPL160W<br>6.1.1.5 : YPL040C<br>6.1.1.17 : YGL245W<br>6.1.1.18 : YOR168W<br>6.1.1.16 : YNL247W<br>6.1.1.19 : YDR341C

Assignments for the pathway ”tRNA charging pathway” (rosetta data set). There are 49152 possible assignments of genes to reactions(only the 10 highest scoring and the 10 lowest scoring assignments are displayed).

Number	Normalized Score	Number of Pairs	Positive Pairs	Negative Pairs	Zero Pairs	Assignments
9	8.36	153	132	21	37	6.1.1.14 : YBR121C<br>6.1.1.21 : YPR033C<br>6.1.1.6 : YDR037W<br>6.1.1.10 : YGR264C<br>6.1.1.20 : 1887<br>6.1.1.15 : YHR020W<br>6.1.1.11 : YDR023W<br>6.1.1.3 : 2924<br>6.1.1.2 : YDR268W<br>6.1.1.1 : YGR185C<br>6.1.1.12 : YLL018C<br>6.1.1.22 : YHR019C<br>6.1.1.7 : YOR335C<br>6.1.1.9 : YGR094W<br>6.1.1.4 : YPL160W<br>6.1.1.5 : YBL076C<br>6.1.1.17 : YGL245W<br>6.1.1.18 : YOR168W<br>6.1.1.16 : YNL247W<br>6.1.1.19 : YDR341C
10	8.36	153	136	17	37	6.1.1.14 : YBR121C<br>6.1.1.21 : YPR033C<br>6.1.1.6 : YDR037W<br>6.1.1.10 : YGR264C<br>6.1.1.20 : 1887<br>6.1.1.15 : YHR020W<br>6.1.1.11 : YDR023W<br>6.1.1.3 : 2924<br>6.1.1.2 : YOL097C<br>6.1.1.1 : YGR185C<br>6.1.1.12 : YLL018C<br>6.1.1.22 : YHR019C<br>6.1.1.7 : YOR335C<br>6.1.1.9 : YGR094W<br>6.1.1.4 : YPL160W<br>6.1.1.5 : YBL076C<br>6.1.1.17 : YGL245W<br>6.1.1.18 : YOR168W<br>6.1.1.16 : YNL247W<br>6.1.1.19 : YHR091C
...<br>...						

Assignments for the pathway ”tRNA charging pathway” (rosetta data set). There are 49152 possible assignments of genes to reactions(only the 10 highest scoring and the 10 lowest scoring assignments are displayed).

Number	Normalized Score	Number of Pairs	Positive Pairs	Negative Pairs	Zero Pairs	Assignments
49143	1.18	171	90	81	19	6.1.1.14 : YPR081C<br>6.1.1.21 : YPR033C<br>6.1.1.6 : YDR037W<br>6.1.1.10 : YGR171C<br>6.1.1.20 : YLR060W<br>6.1.1.15 : YHR020W<br>6.1.1.11 : YHR011W<br>6.1.1.3 : 2924<br>6.1.1.2 : YDR268W<br>6.1.1.1 : YPL097W<br>6.1.1.12 : YPL104W<br>6.1.1.22 : YCR024C<br>6.1.1.7 : YOR335C<br>6.1.1.9 : YGR094W<br>6.1.1.4 : YPL160W<br>6.1.1.5 : YPL040C<br>6.1.1.17 : YOL033W<br>6.1.1.18 : YOR168W<br>6.1.1.16 : YNL247W<br>6.1.1.19 : YHR091C
49144	1.17	171	90	81	19	6.1.1.14 : YPR081C<br>6.1.1.21 : YPR033C<br>6.1.1.6 : YDR037W<br>6.1.1.10 : YGR171C<br>6.1.1.20 : YLR060W<br>6.1.1.15 : YER087W<br>6.1.1.11 : YHR011W<br>6.1.1.3 : 2924<br>6.1.1.2 : YDR268W<br>6.1.1.1 : YPL097W<br>6.1.1.12 : YPL104W<br>6.1.1.22 : YCR024C<br>6.1.1.7 : YOR335C<br>6.1.1.9 : YGR094W<br>6.1.1.4 : YPL160W<br>6.1.1.5 : YPL040C<br>6.1.1.17 : YOL033W<br>6.1.1.18 : YOR168W<br>6.1.1.16 : YNL247W<br>6.1.1.19 : YHR091C

Assignments for the pathway "tRNA charging pathway" (rosetta data set). There are 49152 possible assignments of genes to reactions(only the 10 highest scoring and the 10 lowest scoring assignments are displayed).

Number	Normalized Score	Number of Pairs	Positive Pairs	Negative Pairs	Zero Pairs	Assignments
49145	1.17	153	83	70	37	6.1.1.14 : YPR081C<br>6.1.1.21 : YPR033C<br>6.1.1.6 : YDR037W<br>6.1.1.10 : YGR171C<br>6.1.1.20 : 1887<br>6.1.1.15 : YER087W<br>6.1.1.11 : YHR011W<br>6.1.1.3 : 2924<br>6.1.1.2 : YDR268W<br>6.1.1.1 : YPL097W<br>6.1.1.12 : YLL018C<br>6.1.1.22 : YCR024C<br>6.1.1.7 : YOR335C<br>6.1.1.9 : YGR094W<br>6.1.1.4 : YPL160W<br>6.1.1.5 : YPL040C<br>6.1.1.17 : YOL033W<br>6.1.1.18 : YOR168W<br>6.1.1.16 : YNL247W<br>6.1.1.19 : YHR091C
49146	1.16	153	85	68	37	6.1.1.14 : YPR081C<br>6.1.1.21 : YPR033C<br>6.1.1.6 : YDR037W<br>6.1.1.10 : YGR171C<br>6.1.1.20 : 1887<br>6.1.1.15 : YHR020W<br>6.1.1.11 : YHR011W<br>6.1.1.3 : 2924<br>6.1.1.2 : YDR268W<br>6.1.1.1 : YPL097W<br>6.1.1.12 : YPL104W<br>6.1.1.22 : YCR024C<br>6.1.1.7 : YOR335C<br>6.1.1.9 : YGR094W<br>6.1.1.4 : YLR382C<br>6.1.1.5 : YPL040C<br>6.1.1.17 : YOL033W<br>6.1.1.18 : YOR168W<br>6.1.1.16 : YNL247W<br>6.1.1.19 : YHR091C

Assignments for the pathway ”tRNA charging pathway” (rosetta data set). There are 49152 possible assignments of genes to reactions(only the 10 highest scoring and the 10 lowest scoring assignments are displayed).

Number	Normalized Score	Number of Pairs	Positive Pairs	Negative Pairs	Zero Pairs	Assignments
49147	1.15	171	94	77	19	6.1.1.14 : YPR081C<br>6.1.1.21 : YPR033C<br>6.1.1.6 : YDR037W<br>6.1.1.10 : YGR171C<br>6.1.1.20 : 1887<br>6.1.1.15 : YER087W<br>6.1.1.11 : YHR011W<br>6.1.1.3 : YKL194C<br>6.1.1.2 : YDR268W<br>6.1.1.1 : YPL097W<br>6.1.1.12 : YPL104W<br>6.1.1.22 : YCR024C<br>6.1.1.7 : YOR335C<br>6.1.1.9 : YGR094W<br>6.1.1.4 : YPL160W<br>6.1.1.5 : YPL040C<br>6.1.1.17 : YGL245W<br>6.1.1.18 : YOR168W<br>6.1.1.16 : YNL247W<br>6.1.1.19 : YHR091C
49148	1.15	171	93	78	19	6.1.1.14 : YPR081C<br>6.1.1.21 : YPR033C<br>6.1.1.6 : YDR037W<br>6.1.1.10 : YGR171C<br>6.1.1.20 : 1887<br>6.1.1.15 : YHR020W<br>6.1.1.11 : YHR011W<br>6.1.1.3 : YKL194C<br>6.1.1.2 : YDR268W<br>6.1.1.1 : YPL097W<br>6.1.1.12 : YPL104W<br>6.1.1.22 : YCR024C<br>6.1.1.7 : YOR335C<br>6.1.1.9 : YGR094W<br>6.1.1.4 : YPL160W<br>6.1.1.5 : YPL040C<br>6.1.1.17 : YOL033W<br>6.1.1.18 : YOR168W<br>6.1.1.16 : YNL247W<br>6.1.1.19 : YHR091C

Assignments for the pathway "tRNA charging pathway" (rosetta data set). There are 49152 possible assignments of genes to reactions(only the 10 highest scoring and the 10 lowest scoring assignments are displayed).

Number	Normalized Score	Number of Pairs	Positive Pairs	Negative Pairs	Zero Pairs	Assignments
49149	1.15	153	80	73	37	6.1.1.14 : YPR081C<br>6.1.1.21 : YPR033C<br>6.1.1.6 : YDR037W<br>6.1.1.10 : YGR171C<br>6.1.1.20 : 1887<br>6.1.1.15 : YHR020W<br>6.1.1.11 : YHR011W<br>6.1.1.3 : 2924<br>6.1.1.2 : YDR268W<br>6.1.1.1 : YPL097W<br>6.1.1.12 : YPL104W<br>6.1.1.22 : YCR024C<br>6.1.1.7 : YOR335C<br>6.1.1.9 : YGR094W<br>6.1.1.4 : YLR382C<br>6.1.1.5 : YPL040C<br>6.1.1.17 : YOL033W<br>6.1.1.18 : YOR168W<br>6.1.1.16 : YNL247W<br>6.1.1.19 : YDR341C
49150	1.12	153	81	72	37	6.1.1.14 : YPR081C<br>6.1.1.21 : YPR033C<br>6.1.1.6 : YDR037W<br>6.1.1.10 : YGR171C<br>6.1.1.20 : 1887<br>6.1.1.15 : YHR020W<br>6.1.1.11 : YHR011W<br>6.1.1.3 : 2924<br>6.1.1.2 : YDR268W<br>6.1.1.1 : YPL097W<br>6.1.1.12 : YPL104W<br>6.1.1.22 : YCR024C<br>6.1.1.7 : YOR335C<br>6.1.1.9 : YGR094W<br>6.1.1.4 : YPL160W<br>6.1.1.5 : YPL040C<br>6.1.1.17 : YOL033W<br>6.1.1.18 : YOR168W<br>6.1.1.16 : YNL247W<br>6.1.1.19 : YHR091C

Assignments for the pathway ”tRNA charging pathway” (rosetta data set). There are 49152 possible assignments of genes to reactions(only the 10 highest scoring and the 10 lowest scoring assignments are displayed).

Number	Normalized Score	Number of Pairs	Positive Pairs	Negative Pairs	Zero Pairs	Assignments
49151	1.10	153	82	71	37	6.1.1.14 : YPR081C<br>6.1.1.21 : YPR033C<br>6.1.1.6 : YDR037W<br>6.1.1.10 : YGR171C<br>6.1.1.20 : 1887<br>6.1.1.15 : YER087W<br>6.1.1.11 : YHR011W<br>6.1.1.3 : 2924<br>6.1.1.2 : YDR268W<br>6.1.1.1 : YPL097W<br>6.1.1.12 : YPL104W<br>6.1.1.22 : YCR024C<br>6.1.1.7 : YOR335C<br>6.1.1.9 : YGR094W<br>6.1.1.4 : YPL160W<br>6.1.1.5 : YPL040C<br>6.1.1.17 : YGL245W<br>6.1.1.18 : YOR168W<br>6.1.1.16 : YNL247W<br>6.1.1.19 : YHR091C

Table 1: **Assignments for the pathway "tRNA charging pathway" (rosetta data set). There are 49152 possible assignments of genes to reactions(only the 10 highest scoring and the 10 lowest scoring assignments are displayed).**

Number	Normalized Score	Number of Pairs	Positive Pairs	Negative Pairs	Zero Pairs	Assignments
1	9.92	28	25	3	8	1.1.99.5 : YIL155C<br>2.7.1.40 : YOR347C<br>4.2.1.11 : YHR174W<br>5.4.2.1 : YDL021W<br>2.7.2.3 : 639<br>1.2.1.12 : YGR192C<br>5.3.1.1 : YDR050C<br>1.1.1.8 : YDL022W<br>2.7.1.30 : YHL032C
2	9.73	28	23	5	8	1.1.99.5 : YIL155C<br>2.7.1.40 : YOR347C<br>4.2.1.11 : YHR174W<br>5.4.2.1 : YKL152C<br>2.7.2.3 : 639<br>1.2.1.12 : YGR192C<br>5.3.1.1 : YDR050C<br>1.1.1.8 : YDL022W<br>2.7.1.30 : YHL032C

Assignments for the pathway "aerobic glycerol degradation II" (rosetta data set). There are 180 possible assignments of genes to reactions(only the 10 highest scoring and the 10 lowest scoring assignments are displayed).

Number	Normalized Score	Number of Pairs	Positive Pairs	Negative Pairs	Zero Pairs	Assignments
3	9.54	28	22	6	8	1.1.99.5 : YIL155C<br>2.7.1.40 : YAL038W<br>4.2.1.11 : YHR174W<br>5.4.2.1 : YKL152C<br>2.7.2.3 : 639<br>1.2.1.12 : YJL052W<br>5.3.1.1 : YDR050C<br>1.1.1.8 : YDL022W<br>2.7.1.30 : YHL032C
4	9.38	28	22	6	8	1.1.99.5 : YIL155C<br>2.7.1.40 : YAL038W<br>4.2.1.11 : YHR174W<br>5.4.2.1 : YKL152C<br>2.7.2.3 : 639<br>1.2.1.12 : YGR192C<br>5.3.1.1 : YDR050C<br>1.1.1.8 : YDL022W<br>2.7.1.30 : YHL032C
5	9.37	28	25	3	8	1.1.99.5 : YIL155C<br>2.7.1.40 : YOR347C<br>4.2.1.11 : YGR254W<br>5.4.2.1 : YDL021W<br>2.7.2.3 : 639<br>1.2.1.12 : YGR192C<br>5.3.1.1 : YDR050C<br>1.1.1.8 : YDL022W<br>2.7.1.30 : YHL032C
6	9.07	28	23	5	8	1.1.99.5 : YIL155C<br>2.7.1.40 : YOR347C<br>4.2.1.11 : YGR254W<br>5.4.2.1 : YKL152C<br>2.7.2.3 : 639<br>1.2.1.12 : YGR192C<br>5.3.1.1 : YDR050C<br>1.1.1.8 : YDL022W<br>2.7.1.30 : YHL032C
7	8.98	28	22	6	8	1.1.99.5 : YIL155C<br>2.7.1.40 : YAL038W<br>4.2.1.11 : YGR254W<br>5.4.2.1 : YKL152C<br>2.7.2.3 : 639<br>1.2.1.12 : YJL052W<br>5.3.1.1 : YDR050C<br>1.1.1.8 : YDL022W<br>2.7.1.30 : YHL032C

Assignments for the pathway "aerobic glycerol degradation II" (rosetta data set). There are 180 possible assignments of genes to reactions(only the 10 highest scoring and the 10 lowest scoring assignments are displayed).

Number	Normalized Score	Number of Pairs	Positive Pairs	Negative Pairs	Zero Pairs	Assignments
8	8.96	28	23	5	8	1.1.99.5 : YIL155C<br>2.7.1.40 : YOR347C<br>4.2.1.11 : YHR174W<br>5.4.2.1 : YKL152C<br>2.7.2.3 : 639<br>1.2.1.12 : YJL052W<br>5.3.1.1 : YDR050C<br>1.1.1.8 : YDL022W<br>2.7.1.30 : YHL032C
9	8.83	28	22	6	8	1.1.99.5 : YIL155C<br>2.7.1.40 : YAL038W<br>4.2.1.11 : YGR254W<br>5.4.2.1 : YKL152C<br>2.7.2.3 : 639<br>1.2.1.12 : YGR192C<br>5.3.1.1 : YDR050C<br>1.1.1.8 : YDL022W<br>2.7.1.30 : YHL032C
10	8.63	28	25	3	8	1.1.99.5 : YIL155C<br>2.7.1.40 : YOR347C<br>4.2.1.11 : YOR393W<br>5.4.2.1 : YDL021W<br>2.7.2.3 : 639<br>1.2.1.12 : YGR192C<br>5.3.1.1 : YDR050C<br>1.1.1.8 : YDL022W<br>2.7.1.30 : YHL032C
...<br>...						
171	2.23	28	19	9	8	1.1.99.5 : YIL155C<br>2.7.1.40 : YAL038W<br>4.2.1.11 : YMR323W<br>5.4.2.1 : YOL056W<br>2.7.2.3 : 639<br>1.2.1.12 : YJR009C<br>5.3.1.1 : YDR050C<br>1.1.1.8 : YOL059W<br>2.7.1.30 : YHL032C
172	2.21	28	20	8	8	1.1.99.5 : YIL155C<br>2.7.1.40 : YOR347C<br>4.2.1.11 : YMR323W<br>5.4.2.1 : YOL056W<br>2.7.2.3 : 639<br>1.2.1.12 : YJL052W<br>5.3.1.1 : YDR050C<br>1.1.1.8 : YOL059W<br>2.7.1.30 : YHL032C

Assignments for the pathway "aerobic glycerol degradation II" (rosetta data set). There are 180 possible assignments of genes to reactions(only the 10 highest scoring and the 10 lowest scoring assignments are displayed).

Number	Normalized Score	Number of Pairs	Positive Pairs	Negative Pairs	Zero Pairs	Assignments
173	2.04	28	17	11	8	1.1.99.5 : YIL155C<br>2.7.1.40 : YAL038W<br>4.2.1.11 : YPL281C<br>5.4.2.1 : YOL056W<br>2.7.2.3 : 639<br>1.2.1.12 : YJR009C<br>5.3.1.1 : YDR050C<br>1.1.1.8 : YDL022W<br>2.7.1.30 : YHL032C
174	1.98	28	16	12	8	1.1.99.5 : YIL155C<br>2.7.1.40 : YOR347C<br>4.2.1.11 : YPL281C<br>5.4.2.1 : YOL056W<br>2.7.2.3 : 639<br>1.2.1.12 : YJR009C<br>5.3.1.1 : YDR050C<br>1.1.1.8 : YDL022W<br>2.7.1.30 : YHL032C
175	1.57	28	16	12	8	1.1.99.5 : YIL155C<br>2.7.1.40 : YAL038W<br>4.2.1.11 : YPL281C<br>5.4.2.1 : YOL056W<br>2.7.2.3 : 639<br>1.2.1.12 : YJR009C<br>5.3.1.1 : YDR050C<br>1.1.1.8 : YOL059W<br>2.7.1.30 : YHL032C
176	1.55	28	17	11	8	1.1.99.5 : YIL155C<br>2.7.1.40 : YOR347C<br>4.2.1.11 : YPL281C<br>5.4.2.1 : YOL056W<br>2.7.2.3 : 639<br>1.2.1.12 : YJL052W<br>5.3.1.1 : YDR050C<br>1.1.1.8 : YOL059W<br>2.7.1.30 : YHL032C
177	1.52	28	19	9	8	1.1.99.5 : YIL155C<br>2.7.1.40 : YOR347C<br>4.2.1.11 : YOR393W<br>5.4.2.1 : YOL056W<br>2.7.2.3 : 639<br>1.2.1.12 : YJR009C<br>5.3.1.1 : YDR050C<br>1.1.1.8 : YOL059W<br>2.7.1.30 : YHL032C

Assignments for the pathway "aerobic glycerol degradation II" (rosetta data set). There are 180 possible assignments of genes to reactions(only the 10 highest scoring and the 10 lowest scoring assignments are displayed).

Number	Normalized Score	Number of Pairs	Positive Pairs	Negative Pairs	Zero Pairs	Assignments
178	1.32	28	18	10	8	1.1.99.5 : YIL155C<br>2.7.1.40 : YOR347C<br>4.2.1.11 : YMR323W<br>5.4.2.1 : YOL056W<br>2.7.2.3 : 639<br>1.2.1.12 : YJR009C<br>5.3.1.1 : YDR050C<br>1.1.1.8 : YOL059W<br>2.7.1.30 : YHL032C
179	0.67	28	15	13	8	1.1.99.5 : YIL155C<br>2.7.1.40 : YOR347C<br>4.2.1.11 : YPL281C<br>5.4.2.1 : YOL056W<br>2.7.2.3 : 639<br>1.2.1.12 : YJR009C<br>5.3.1.1 : YDR050C<br>1.1.1.8 : YOL059W<br>2.7.1.30 : YHL032C

Table 2: **Assignments for the pathway "aerobic glycerol degradation II" (rosetta data set). There are 180 possible assignments of genes to reactions(only the 10 highest scoring and the 10 lowest scoring assignments are displayed).**

Number	Normalized Score	Number of Pairs	Positive Pairs	Negative Pairs	Zero Pairs	Assignments
1	16.01	28	28	0	8	5.3.1.9 : YBR196C<br>2.7.1.11 : YMR205C<br>4.1.2.13 : YKL060C<br>5.3.1.1 : YDR050C<br>1.2.1.12 : YJL052W<br>2.7.2.3 : 639<br>5.4.2.1 : YKL152C<br>4.2.1.11 : YHR174W<br>2.7.1.40 : YAL038W
2	15.72	28	28	0	8	5.3.1.9 : YBR196C<br>2.7.1.11 : YGR240C<br>4.1.2.13 : YKL060C<br>5.3.1.1 : YDR050C<br>1.2.1.12 : YJL052W<br>2.7.2.3 : 639<br>5.4.2.1 : YKL152C<br>4.2.1.11 : YHR174W<br>2.7.1.40 : YAL038W

Assignments for the pathway "glycolysis" (rosetta data set). There are 180 possible assignments of genes to reactions(only the 10 highest scoring and the 10 lowest scoring assignments are displayed).

Number	Normalized Score	Number of Pairs	Positive Pairs	Negative Pairs	Zero Pairs	Assignments
3	15.05	28	28	0	8	5.3.1.9 : YBR196C<br>2.7.1.11 : YMR205C<br>4.1.2.13 : YKL060C<br>5.3.1.1 : YDR050C<br>1.2.1.12 : YJL052W<br>2.7.2.3 : 639<br>5.4.2.1 : YKL152C<br>4.2.1.11 : YGR254W<br>2.7.1.40 : YAL038W
4	15.01	28	28	0	8	5.3.1.9 : YBR196C<br>2.7.1.11 : YGR240C<br>4.1.2.13 : YKL060C<br>5.3.1.1 : YDR050C<br>1.2.1.12 : YJL052W<br>2.7.2.3 : 639<br>5.4.2.1 : YKL152C<br>4.2.1.11 : YGR254W<br>2.7.1.40 : YAL038W
5	14.49	28	28	0	8	5.3.1.9 : YBR196C<br>2.7.1.11 : YMR205C<br>4.1.2.13 : YKL060C<br>5.3.1.1 : YDR050C<br>1.2.1.12 : YGR192C<br>2.7.2.3 : 639<br>5.4.2.1 : YKL152C<br>4.2.1.11 : YHR174W<br>2.7.1.40 : YAL038W
6	14.27	28	28	0	8	5.3.1.9 : YBR196C<br>2.7.1.11 : YGR240C<br>4.1.2.13 : YKL060C<br>5.3.1.1 : YDR050C<br>1.2.1.12 : YGR192C<br>2.7.2.3 : 639<br>5.4.2.1 : YKL152C<br>4.2.1.11 : YHR174W<br>2.7.1.40 : YAL038W
7	14.15	28	28	0	8	5.3.1.9 : YBR196C<br>2.7.1.11 : YGR240C<br>4.1.2.13 : YKL060C<br>5.3.1.1 : YDR050C<br>1.2.1.12 : YJL052W<br>2.7.2.3 : 639<br>5.4.2.1 : YKL152C<br>4.2.1.11 : YHR174W<br>2.7.1.40 : YOR347C

Assignments for the pathway "glycolysis" (rosetta data set). There are 180 possible assignments of genes to reactions(only the 10 highest scoring and the 10 lowest scoring assignments are displayed).

Number	Normalized Score	Number of Pairs	Positive Pairs	Negative Pairs	Zero Pairs	Assignments
8	13.96	28	28	0	8	5.3.1.9 : YBR196C<br>2.7.1.11 : YMR205C<br>4.1.2.13 : YKL060C<br>5.3.1.1 : YDR050C<br>1.2.1.12 : YJL052W<br>2.7.2.3 : 639<br>5.4.2.1 : YKL152C<br>4.2.1.11 : YHR174W<br>2.7.1.40 : YOR347C
9	13.63	28	28	0	8	5.3.1.9 : YBR196C<br>2.7.1.11 : YGR240C<br>4.1.2.13 : YKL060C<br>5.3.1.1 : YDR050C<br>1.2.1.12 : YGR192C<br>2.7.2.3 : 639<br>5.4.2.1 : YKL152C<br>4.2.1.11 : YHR174W<br>2.7.1.40 : YOR347C
10	13.57	28	28	0	8	5.3.1.9 : YBR196C<br>2.7.1.11 : YGR240C<br>4.1.2.13 : YKL060C<br>5.3.1.1 : YDR050C<br>1.2.1.12 : YGR192C<br>2.7.2.3 : 639<br>5.4.2.1 : YKL152C<br>4.2.1.11 : YGR254W<br>2.7.1.40 : YAL038W
...<br>...						
171	5.98	28	23	5	8	5.3.1.9 : YBR196C<br>2.7.1.11 : YGR240C<br>4.1.2.13 : YKL060C<br>5.3.1.1 : YDR050C<br>1.2.1.12 : YJR009C<br>2.7.2.3 : 639<br>5.4.2.1 : YDL021W<br>4.2.1.11 : YMR323W<br>2.7.1.40 : YAL038W
172	5.96	28	24	4	8	5.3.1.9 : YBR196C<br>2.7.1.11 : YGR240C<br>4.1.2.13 : YKL060C<br>5.3.1.1 : YDR050C<br>1.2.1.12 : YJR009C<br>2.7.2.3 : 639<br>5.4.2.1 : YOL056W<br>4.2.1.11 : YMR323W<br>2.7.1.40 : YAL038W

Assignments for the pathway "glycolysis" (rosetta data set). There are 180 possible assignments of genes to reactions(only the 10 highest scoring and the 10 lowest scoring assignments are displayed).

Number	Normalized Score	Number of Pairs	Positive Pairs	Negative Pairs	Zero Pairs	Assignments
173	5.95	28	23	5	8	5.3.1.9 : YBR196C<br>2.7.1.11 : YMR205C<br>4.1.2.13 : YKL060C<br>5.3.1.1 : YDR050C<br>1.2.1.12 : YJR009C<br>2.7.2.3 : 639<br>5.4.2.1 : YDL021W<br>4.2.1.11 : YMR323W<br>2.7.1.40 : YOR347C
174	5.73	28	25	3	8	5.3.1.9 : YBR196C<br>2.7.1.11 : YGR240C<br>4.1.2.13 : YKL060C<br>5.3.1.1 : YDR050C<br>1.2.1.12 : YJR009C<br>2.7.2.3 : 639<br>5.4.2.1 : YOL056W<br>4.2.1.11 : YOR393W<br>2.7.1.40 : YOR347C
175	5.48	28	25	3	8	5.3.1.9 : YBR196C<br>2.7.1.11 : YMR205C<br>4.1.2.13 : YKL060C<br>5.3.1.1 : YDR050C<br>1.2.1.12 : YJR009C<br>2.7.2.3 : 639<br>5.4.2.1 : YOL056W<br>4.2.1.11 : YOR393W<br>2.7.1.40 : YOR347C
176	5.27	28	24	4	8	5.3.1.9 : YBR196C<br>2.7.1.11 : YGR240C<br>4.1.2.13 : YKL060C<br>5.3.1.1 : YDR050C<br>1.2.1.12 : YJR009C<br>2.7.2.3 : 639<br>5.4.2.1 : YOL056W<br>4.2.1.11 : YPL281C<br>2.7.1.40 : YOR347C
177	4.91	28	22	6	8	5.3.1.9 : YBR196C<br>2.7.1.11 : YGR240C<br>4.1.2.13 : YKL060C<br>5.3.1.1 : YDR050C<br>1.2.1.12 : YJR009C<br>2.7.2.3 : 639<br>5.4.2.1 : YOL056W<br>4.2.1.11 : YMR323W<br>2.7.1.40 : YOR347C

Assignments for the pathway "glycolysis" (rosetta data set). There are 180 possible assignments of genes to reactions(only the 10 highest scoring and the 10 lowest scoring assignments are displayed).

Number	Normalized Score	Number of Pairs	Positive Pairs	Negative Pairs	Zero Pairs	Assignments
178	4.84	28	23	5	8	5.3.1.9 : YBR196C<br>2.7.1.11 : YMR205C<br>4.1.2.13 : YKL060C<br>5.3.1.1 : YDR050C<br>1.2.1.12 : YJR009C<br>2.7.2.3 : 639<br>5.4.2.1 : YOL056W<br>4.2.1.11 : YMR323W<br>2.7.1.40 : YOR347C
179	4.80	28	23	5	8	5.3.1.9 : YBR196C<br>2.7.1.11 : YMR205C<br>4.1.2.13 : YKL060C<br>5.3.1.1 : YDR050C<br>1.2.1.12 : YJR009C<br>2.7.2.3 : 639<br>5.4.2.1 : YOL056W<br>4.2.1.11 : YPL281C<br>2.7.1.40 : YOR347C

Table 3: **Assignments for the pathway "glycolysis" (rosetta data set). There are 180 possible assignments of genes to reactions(only the 10 highest scoring and the 10 lowest scoring assignments are displayed).**

Number	Normalized Score	Number of Pairs	Positive Pairs	Negative Pairs	Zero Pairs	Assignments
1	5.02	10	7	3	0	4.2.1.3 : YLR304C<br>4.1.3.7 : YNR001C<br>4.1.3.1 : YPR006C<br>4.1.3.2 : YIR031C<br>1.1.1.37 : YDL078C
2	4.93	10	9	1	0	4.2.1.3 : YJL200C<br>4.1.3.7 : YPR001W<br>4.1.3.1 : YER065C<br>4.1.3.2 : YIR031C<br>1.1.1.37 : YDL078C
3	4.76	10	9	1	0	4.2.1.3 : YJL200C<br>4.1.3.7 : YPR001W<br>4.1.3.1 : YER065C<br>4.1.3.2 : YNL117W<br>1.1.1.37 : YDL078C
4	4.76	10	6	4	0	4.2.1.3 : YLR304C<br>4.1.3.7 : YNR001C<br>4.1.3.1 : YPR006C<br>4.1.3.2 : YNL117W<br>1.1.1.37 : YKL085W

Assignments for the pathway "glyoxylate cycle" (rosetta data set). There are 72 possible assignments of genes to reactions.

Number	Normalized Score	Number of Pairs	Positive Pairs	Negative Pairs	Zero Pairs	Assignments
5	4.74	10	8	2	0	4.2.1.3 : YLR304C<br>4.1.3.7 : YNR001C<br>4.1.3.1 : YPR006C<br>4.1.3.2 : YNL117W<br>1.1.1.37 : YDL078C
6	4.41	10	5	5	0	4.2.1.3 : YLR304C<br>4.1.3.7 : YNR001C<br>4.1.3.1 : YPR006C<br>4.1.3.2 : YIR031C<br>1.1.1.37 : YKL085W
7	4.32	10	9	1	0	4.2.1.3 : YLR304C<br>4.1.3.7 : YCR005C<br>4.1.3.1 : YPR006C<br>4.1.3.2 : YNL117W<br>1.1.1.37 : YDL078C
8	4.22	10	7	3	0	4.2.1.3 : YLR304C<br>4.1.3.7 : YCR005C<br>4.1.3.1 : YPR006C<br>4.1.3.2 : YIR031C<br>1.1.1.37 : YDL078C
9	3.82	10	6	4	0	4.2.1.3 : YJL200C<br>4.1.3.7 : YPR001W<br>4.1.3.1 : YER065C<br>4.1.3.2 : YNL117W<br>1.1.1.37 : YOL126C
10	3.55	10	6	4	0	4.2.1.3 : YJL200C<br>4.1.3.7 : YPR001W<br>4.1.3.1 : YPR006C<br>4.1.3.2 : YIR031C<br>1.1.1.37 : YDL078C
11	3.51	10	6	4	0	4.2.1.3 : YJL200C<br>4.1.3.7 : YPR001W<br>4.1.3.1 : YER065C<br>4.1.3.2 : YIR031C<br>1.1.1.37 : YOL126C
12	3.37	10	6	4	0	4.2.1.3 : YJL200C<br>4.1.3.7 : YPR001W<br>4.1.3.1 : YPR006C<br>4.1.3.2 : YNL117W<br>1.1.1.37 : YDL078C
13	3.22	10	7	3	0	4.2.1.3 : YLR304C<br>4.1.3.7 : YNR001C<br>4.1.3.1 : YER065C<br>4.1.3.2 : YIR031C<br>1.1.1.37 : YDL078C

Assignments for the pathway "glyoxylate cycle" (rosetta data set). There are 72 possible assignments of genes to reactions.

Number	Normalized Score	Number of Pairs	Positive Pairs	Negative Pairs	Zero Pairs	Assignments
14	3.19	10	6	4	0	4.2.1.3 : YLR304C<br>4.1.3.7 : YCR005C<br>4.1.3.1 : YPR006C<br>4.1.3.2 : YNL117W<br>1.1.1.37 : YKL085W
15	3.11	10	6	4	0	4.2.1.3 : YLR304C<br>4.1.3.7 : YNR001C<br>4.1.3.1 : YPR006C<br>4.1.3.2 : YNL117W<br>1.1.1.37 : YOL126C
16	3.02	10	6	4	0	4.2.1.3 : YLR304C<br>4.1.3.7 : YNR001C<br>4.1.3.1 : YER065C<br>4.1.3.2 : YNL117W<br>1.1.1.37 : YKL085W
17	2.96	10	8	2	0	4.2.1.3 : YLR304C<br>4.1.3.7 : YNR001C<br>4.1.3.1 : YER065C<br>4.1.3.2 : YNL117W<br>1.1.1.37 : YDL078C
18	2.92	10	5	5	0	4.2.1.3 : YLR304C<br>4.1.3.7 : YNR001C<br>4.1.3.1 : YPR006C<br>4.1.3.2 : YIR031C<br>1.1.1.37 : YOL126C
19	2.82	10	6	4	0	4.2.1.3 : YLR304C<br>4.1.3.7 : YCR005C<br>4.1.3.1 : YPR006C<br>4.1.3.2 : YNL117W<br>1.1.1.37 : YOL126C
20	2.66	10	5	5	0	4.2.1.3 : YLR304C<br>4.1.3.7 : YNR001C<br>4.1.3.1 : YER065C<br>4.1.3.2 : YIR031C<br>1.1.1.37 : YKL085W
21	2.51	10	5	5	0	4.2.1.3 : YJL200C<br>4.1.3.7 : YNR001C<br>4.1.3.1 : YPR006C<br>4.1.3.2 : YIR031C<br>1.1.1.37 : YDL078C
22	2.48	10	6	4	0	4.2.1.3 : YJL200C<br>4.1.3.7 : YPR001W<br>4.1.3.1 : YER065C<br>4.1.3.2 : YNL117W<br>1.1.1.37 : YKL085W

Assignments for the pathway "glyoxylate cycle" (rosetta data set). There are 72 possible assignments of genes to reactions.

Number	Normalized Score	Number of Pairs	Positive Pairs	Negative Pairs	Zero Pairs	Assignments
23	2.48	10	6	4	0	4.2.1.3 : YJL200C<br>4.1.3.7 : YCR005C<br>4.1.3.1 : YPR006C<br>4.1.3.2 : YIR031C<br>1.1.1.37 : YDL078C
24	2.45	10	4	6	0	4.2.1.3 : YLR304C<br>4.1.3.7 : YCR005C<br>4.1.3.1 : YPR006C<br>4.1.3.2 : YIR031C<br>1.1.1.37 : YKL085W
25	2.25	10	4	6	0	4.2.1.3 : YLR304C<br>4.1.3.7 : YCR005C<br>4.1.3.1 : YPR006C<br>4.1.3.2 : YIR031C<br>1.1.1.37 : YOL126C
26	2.24	10	4	6	0	4.2.1.3 : YJL200C<br>4.1.3.7 : YPR001W<br>4.1.3.1 : YPR006C<br>4.1.3.2 : YNL117W<br>1.1.1.37 : YOL126C
27	2.22	10	8	2	0	4.2.1.3 : YLR304C<br>4.1.3.7 : YCR005C<br>4.1.3.1 : YER065C<br>4.1.3.2 : YNL117W<br>1.1.1.37 : YDL078C
28	2.14	10	7	3	0	4.2.1.3 : YLR304C<br>4.1.3.7 : YPR001W<br>4.1.3.1 : YPR006C<br>4.1.3.2 : YNL117W<br>1.1.1.37 : YDL078C
29	2.13	10	8	2	0	4.2.1.3 : YLR304C<br>4.1.3.7 : YPR001W<br>4.1.3.1 : YER065C<br>4.1.3.2 : YNL117W<br>1.1.1.37 : YDL078C
30	2.11	10	7	3	0	4.2.1.3 : YJL200C<br>4.1.3.7 : YNR001C<br>4.1.3.1 : YER065C<br>4.1.3.2 : YIR031C<br>1.1.1.37 : YDL078C
31	2.10	10	6	4	0	4.2.1.3 : YLR304C<br>4.1.3.7 : YCR005C<br>4.1.3.1 : YER065C<br>4.1.3.2 : YIR031C<br>1.1.1.37 : YDL078C

Assignments for the pathway "glyoxylate cycle" (rosetta data set). There are 72 possible assignments of genes to reactions.

Number	Normalized Score	Number of Pairs	Positive Pairs	Negative Pairs	Zero Pairs	Assignments
32	2.08	10	7	3	0	4.2.1.3 : YJL200C<br>4.1.3.7 : YCR005C<br>4.1.3.1 : YPR006C<br>4.1.3.2 : YNL117W<br>1.1.1.37 : YDL078C
33	2.01	10	6	4	0	4.2.1.3 : YJL200C<br>4.1.3.7 : YPR001W<br>4.1.3.1 : YER065C<br>4.1.3.2 : YIR031C<br>1.1.1.37 : YKL085W
34	1.96	10	4	6	0	4.2.1.3 : YJL200C<br>4.1.3.7 : YPR001W<br>4.1.3.1 : YPR006C<br>4.1.3.2 : YIR031C<br>1.1.1.37 : YOL126C
35	1.82	10	6	4	0	4.2.1.3 : YLR304C<br>4.1.3.7 : YPR001W<br>4.1.3.1 : YPR006C<br>4.1.3.2 : YIR031C<br>1.1.1.37 : YDL078C
36	1.80	10	7	3	0	4.2.1.3 : YLR304C<br>4.1.3.7 : YPR001W<br>4.1.3.1 : YER065C<br>4.1.3.2 : YIR031C<br>1.1.1.37 : YDL078C
37	1.76	10	7	3	0	4.2.1.3 : YJL200C<br>4.1.3.7 : YCR005C<br>4.1.3.1 : YER065C<br>4.1.3.2 : YIR031C<br>1.1.1.37 : YDL078C
38	1.73	10	5	5	0	4.2.1.3 : YJL200C<br>4.1.3.7 : YNR001C<br>4.1.3.1 : YPR006C<br>4.1.3.2 : YNL117W<br>1.1.1.37 : YDL078C
39	1.50	10	5	5	0	4.2.1.3 : YLR304C<br>4.1.3.7 : YNR001C<br>4.1.3.1 : YER065C<br>4.1.3.2 : YNL117W<br>1.1.1.37 : YOL126C
40	1.39	10	8	2	0	4.2.1.3 : YJL200C<br>4.1.3.7 : YCR005C<br>4.1.3.1 : YER065C<br>4.1.3.2 : YNL117W<br>1.1.1.37 : YDL078C

Assignments for the pathway "glyoxylate cycle" (rosetta data set). There are 72 possible assignments of genes to reactions.

Number	Normalized Score	Number of Pairs	Positive Pairs	Negative Pairs	Zero Pairs	Assignments
41	1.35	10	7	3	0	4.2.1.3 : YJL200C<br>4.1.3.7 : YNR001C<br>4.1.3.1 : YER065C<br>4.1.3.2 : YNL117W<br>1.1.1.37 : YDL078C
42	1.30	10	4	6	0	4.2.1.3 : YLR304C<br>4.1.3.7 : YNR001C<br>4.1.3.1 : YER065C<br>4.1.3.2 : YIR031C<br>1.1.1.37 : YOL126C
43	1.14	10	5	5	0	4.2.1.3 : YLR304C<br>4.1.3.7 : YCR005C<br>4.1.3.1 : YER065C<br>4.1.3.2 : YNL117W<br>1.1.1.37 : YKL085W
44	1.11	10	4	6	0	4.2.1.3 : YLR304C<br>4.1.3.7 : YPR001W<br>4.1.3.1 : YER065C<br>4.1.3.2 : YNL117W<br>1.1.1.37 : YOL126C
45	1.03	10	3	7	0	4.2.1.3 : YJL200C<br>4.1.3.7 : YPR001W<br>4.1.3.1 : YPR006C<br>4.1.3.2 : YNL117W<br>1.1.1.37 : YKL085W
46	1.02	10	5	5	0	4.2.1.3 : YLR304C<br>4.1.3.7 : YPR001W<br>4.1.3.1 : YER065C<br>4.1.3.2 : YNL117W<br>1.1.1.37 : YKL085W
47	0.98	10	4	6	0	4.2.1.3 : YLR304C<br>4.1.3.7 : YPR001W<br>4.1.3.1 : YPR006C<br>4.1.3.2 : YNL117W<br>1.1.1.37 : YKL085W
48	0.94	10	4	6	0	4.2.1.3 : YLR304C<br>4.1.3.7 : YPR001W<br>4.1.3.1 : YPR006C<br>4.1.3.2 : YNL117W<br>1.1.1.37 : YOL126C
49	0.90	10	4	6	0	4.2.1.3 : YLR304C<br>4.1.3.7 : YCR005C<br>4.1.3.1 : YER065C<br>4.1.3.2 : YNL117W<br>1.1.1.37 : YOL126C

Assignments for the pathway "glyoxylate cycle" (rosetta data set). There are 72 possible assignments of genes to reactions.

Number	Normalized Score	Number of Pairs	Positive Pairs	Negative Pairs	Zero Pairs	Assignments
50	0.71	10	3	7	0	4.2.1.3 : YJL200C<br>4.1.3.7 : YNR001C<br>4.1.3.1 : YPR006C<br>4.1.3.2 : YIR031C<br>1.1.1.37 : YKL085W
51	0.66	10	5	5	0	4.2.1.3 : YJL200C<br>4.1.3.7 : YCR005C<br>4.1.3.1 : YPR006C<br>4.1.3.2 : YNL117W<br>1.1.1.37 : YOL126C
52	0.58	10	4	6	0	4.2.1.3 : YJL200C<br>4.1.3.7 : YCR005C<br>4.1.3.1 : YPR006C<br>4.1.3.2 : YIR031C<br>1.1.1.37 : YOL126C
53	0.58	10	3	7	0	4.2.1.3 : YJL200C<br>4.1.3.7 : YPR001W<br>4.1.3.1 : YPR006C<br>4.1.3.2 : YIR031C<br>1.1.1.37 : YKL085W
54	0.57	10	3	7	0	4.2.1.3 : YJL200C<br>4.1.3.7 : YNR001C<br>4.1.3.1 : YPR006C<br>4.1.3.2 : YNL117W<br>1.1.1.37 : YKL085W
55	0.48	10	4	6	0	4.2.1.3 : YJL200C<br>4.1.3.7 : YNR001C<br>4.1.3.1 : YPR006C<br>4.1.3.2 : YIR031C<br>1.1.1.37 : YOL126C
56	0.39	10	3	7	0	4.2.1.3 : YLR304C<br>4.1.3.7 : YCR005C<br>4.1.3.1 : YER065C<br>4.1.3.2 : YIR031C<br>1.1.1.37 : YKL085W
57	0.37	10	5	5	0	4.2.1.3 : YJL200C<br>4.1.3.7 : YNR001C<br>4.1.3.1 : YER065C<br>4.1.3.2 : YIR031C<br>1.1.1.37 : YKL085W
58	0.32	10	2	8	0	4.2.1.3 : YLR304C<br>4.1.3.7 : YCR005C<br>4.1.3.1 : YER065C<br>4.1.3.2 : YIR031C<br>1.1.1.37 : YOL126C

Assignments for the pathway "glyoxylate cycle" (rosetta data set). There are 72 possible assignments of genes to reactions.

Number	Normalized Score	Number of Pairs	Positive Pairs	Negative Pairs	Zero Pairs	Assignments
59	0.31	10	3	7	0	4.2.1.3 : YLR304C<br>4.1.3.7 : YPR001W<br>4.1.3.1 : YER065C<br>4.1.3.2 : YIR031C<br>1.1.1.37 : YOL126C
60	0.26	10	5	5	0	4.2.1.3 : YJL200C<br>4.1.3.7 : YNR001C<br>4.1.3.1 : YER065C<br>4.1.3.2 : YIR031C<br>1.1.1.37 : YOL126C
61	0.24	10	5	5	0	4.2.1.3 : YJL200C<br>4.1.3.7 : YNR001C<br>4.1.3.1 : YER065C<br>4.1.3.2 : YNL117W<br>1.1.1.37 : YKL085W
62	0.17	10	4	6	0	4.2.1.3 : YJL200C<br>4.1.3.7 : YNR001C<br>4.1.3.1 : YPR006C<br>4.1.3.2 : YNL117W<br>1.1.1.37 : YOL126C
63	0.16	10	3	7	0	4.2.1.3 : YLR304C<br>4.1.3.7 : YPR001W<br>4.1.3.1 : YPR006C<br>4.1.3.2 : YIR031C<br>1.1.1.37 : YOL126C
64	0.14	10	5	5	0	4.2.1.3 : YJL200C<br>4.1.3.7 : YCR005C<br>4.1.3.1 : YER065C<br>4.1.3.2 : YNL117W<br>1.1.1.37 : YOL126C
65	0.06	10	4	6	0	4.2.1.3 : YLR304C<br>4.1.3.7 : YPR001W<br>4.1.3.1 : YER065C<br>4.1.3.2 : YIR031C<br>1.1.1.37 : YKL085W
66	0.05	10	4	6	0	4.2.1.3 : YJL200C<br>4.1.3.7 : YCR005C<br>4.1.3.1 : YER065C<br>4.1.3.2 : YIR031C<br>1.1.1.37 : YOL126C
67	0.03	10	3	7	0	4.2.1.3 : YLR304C<br>4.1.3.7 : YPR001W<br>4.1.3.1 : YPR006C<br>4.1.3.2 : YIR031C<br>1.1.1.37 : YKL085W

Assignments for the pathway "glyoxylate cycle" (rosetta data set). There are 72 possible assignments of genes to reactions.

Number	Normalized Score	Number of Pairs	Positive Pairs	Negative Pairs	Zero Pairs	Assignments
68	-0.03	10	5	5	0	4.2.1.3 : YJL200C<br>4.1.3.7 : YNR001C<br>4.1.3.1 : YER065C<br>4.1.3.2 : YNL117W<br>1.1.1.37 : YOL126C
69	-0.23	10	4	6	0	4.2.1.3 : YJL200C<br>4.1.3.7 : YCR005C<br>4.1.3.1 : YPR006C<br>4.1.3.2 : YNL117W<br>1.1.1.37 : YKL085W
70	-0.47	10	3	7	0	4.2.1.3 : YJL200C<br>4.1.3.7 : YCR005C<br>4.1.3.1 : YPR006C<br>4.1.3.2 : YIR031C<br>1.1.1.37 : YKL085W
71	-0.87	10	5	5	0	4.2.1.3 : YJL200C<br>4.1.3.7 : YCR005C<br>4.1.3.1 : YER065C<br>4.1.3.2 : YNL117W<br>1.1.1.37 : YKL085W
72	-1.13	10	4	6	0	4.2.1.3 : YJL200C<br>4.1.3.7 : YCR005C<br>4.1.3.1 : YER065C<br>4.1.3.2 : YIR031C<br>1.1.1.37 : YKL085W

Table 4: **Assignments for the pathway "glyoxylate cycle" (rosetta data set). There are 72 possible assignments of genes to reactions.**

Number	Normalized Score	Number of Pairs	Positive Pairs	Negative Pairs	Zero Pairs	Assignments
1	6.57	55	36	19	0	6.3.4.3 : YBR084W<br>6.3.2.17 : YKL132C<br>3.5.4.16 : YGR267C<br>4.1.2.25 : YNL256W<br>2.7.6.3 : YNL256W<br>1.5.1.3 : YOR236W<br>6.3.2.12 : YMR113W<br>2.1.2.1 : YLR058C<br>3.5.4.9 : YBR084W<br>1.5.1.5 : YBR084W<br>2.5.1.15 : YNL256W

Assignments for the pathway "folic acid biosynthesis" (rosetta data set). There are 48 possible assignments of genes to reactions.

Number	Normalized Score	Number of Pairs	Positive Pairs	Negative Pairs	Zero Pairs	Assignments
2	6.24	55	36	19	0	6.3.4.3 : YBR084W<br>6.3.2.17 : YKL132C<br>3.5.4.16 : YGR267C<br>4.1.2.25 : YNL256W<br>2.7.6.3 : YNL256W<br>1.5.1.3 : YOR236W<br>6.3.2.12 : YMR113W<br>2.1.2.1 : YBR263W<br>3.5.4.9 : YBR084W<br>1.5.1.5 : YBR084W<br>2.5.1.15 : YNL256W
3	5.72	55	33	22	0	6.3.4.3 : YBR084W<br>6.3.2.17 : YOR241W<br>3.5.4.16 : YGR267C<br>4.1.2.25 : YNL256W<br>2.7.6.3 : YNL256W<br>1.5.1.3 : YOR236W<br>6.3.2.12 : YMR113W<br>2.1.2.1 : YLR058C<br>3.5.4.9 : YBR084W<br>1.5.1.5 : YBR084W<br>2.5.1.15 : YNL256W
4	5.39	55	34	21	0	6.3.4.3 : YBR084W<br>6.3.2.17 : YOR241W<br>3.5.4.16 : YGR267C<br>4.1.2.25 : YNL256W<br>2.7.6.3 : YNL256W<br>1.5.1.3 : YOR236W<br>6.3.2.12 : YMR113W<br>2.1.2.1 : YBR263W<br>3.5.4.9 : YBR084W<br>1.5.1.5 : YBR084W<br>2.5.1.15 : YNL256W
5	5.32	55	35	20	0	6.3.4.3 : YBR084W<br>6.3.2.17 : YMR113W<br>3.5.4.16 : YGR267C<br>4.1.2.25 : YNL256W<br>2.7.6.3 : YNL256W<br>1.5.1.3 : YOR236W<br>6.3.2.12 : YMR113W<br>2.1.2.1 : YLR058C<br>3.5.4.9 : YBR084W<br>1.5.1.5 : YBR084W<br>2.5.1.15 : YNL256W

Assignments for the pathway "folic acid biosynthesis" (rosetta data set). There are 48 possible assignments of genes to reactions.

Number	Normalized Score	Number of Pairs	Positive Pairs	Negative Pairs	Zero Pairs	Assignments
6	4.89	55	35	20	0	6.3.4.3 : YBR084W<br>6.3.2.17 : YMR113W<br>3.5.4.16 : YGR267C<br>4.1.2.25 : YNL256W<br>2.7.6.3 : YNL256W<br>1.5.1.3 : YOR236W<br>6.3.2.12 : YMR113W<br>2.1.2.1 : YBR263W<br>3.5.4.9 : YBR084W<br>1.5.1.5 : YBR084W<br>2.5.1.15 : YNL256W
7	4.37	55	32	23	0	6.3.4.3 : YBR084W<br>6.3.2.17 : YKL132C<br>3.5.4.16 : YGR267C<br>4.1.2.25 : YNL256W<br>2.7.6.3 : YNL256W<br>1.5.1.3 : YOR236W<br>6.3.2.12 : YMR113W<br>2.1.2.1 : YLR058C<br>3.5.4.9 : YGR204W<br>1.5.1.5 : YBR084W<br>2.5.1.15 : YNL256W
8	4.37	55	32	23	0	6.3.4.3 : YGR204W<br>6.3.2.17 : YKL132C<br>3.5.4.16 : YGR267C<br>4.1.2.25 : YNL256W<br>2.7.6.3 : YNL256W<br>1.5.1.3 : YOR236W<br>6.3.2.12 : YMR113W<br>2.1.2.1 : YLR058C<br>3.5.4.9 : YBR084W<br>1.5.1.5 : YBR084W<br>2.5.1.15 : YNL256W
9	4.37	55	32	23	0	6.3.4.3 : YBR084W<br>6.3.2.17 : YKL132C<br>3.5.4.16 : YGR267C<br>4.1.2.25 : YNL256W<br>2.7.6.3 : YNL256W<br>1.5.1.3 : YOR236W<br>6.3.2.12 : YMR113W<br>2.1.2.1 : YLR058C<br>3.5.4.9 : YBR084W<br>1.5.1.5 : YGR204W<br>2.5.1.15 : YNL256W

Assignments for the pathway "folic acid biosynthesis" (rosetta data set). There are 48 possible assignments of genes to reactions.

Number	Normalized Score	Number of Pairs	Positive Pairs	Negative Pairs	Zero Pairs	Assignments
10	3.89	55	31	24	0	6.3.4.3 : YBR084W<br>6.3.2.17 : YKL132C<br>3.5.4.16 : YGR267C<br>4.1.2.25 : YNL256W<br>2.7.6.3 : YNL256W<br>1.5.1.3 : YOR236W<br>6.3.2.12 : YMR113W<br>2.1.2.1 : YBR263W<br>3.5.4.9 : YGR204W<br>1.5.1.5 : YBR084W<br>2.5.1.15 : YNL256W
11	3.89	55	31	24	0	6.3.4.3 : YBR084W<br>6.3.2.17 : YKL132C<br>3.5.4.16 : YGR267C<br>4.1.2.25 : YNL256W<br>2.7.6.3 : YNL256W<br>1.5.1.3 : YOR236W<br>6.3.2.12 : YMR113W<br>2.1.2.1 : YBR263W<br>3.5.4.9 : YBR084W<br>1.5.1.5 : YGR204W<br>2.5.1.15 : YNL256W
12	3.89	55	31	24	0	6.3.4.3 : YGR204W<br>6.3.2.17 : YKL132C<br>3.5.4.16 : YGR267C<br>4.1.2.25 : YNL256W<br>2.7.6.3 : YNL256W<br>1.5.1.3 : YOR236W<br>6.3.2.12 : YMR113W<br>2.1.2.1 : YBR263W<br>3.5.4.9 : YBR084W<br>1.5.1.5 : YBR084W<br>2.5.1.15 : YNL256W
13	3.55	55	29	26	0	6.3.4.3 : YBR084W<br>6.3.2.17 : YOR241W<br>3.5.4.16 : YGR267C<br>4.1.2.25 : YNL256W<br>2.7.6.3 : YNL256W<br>1.5.1.3 : YOR236W<br>6.3.2.12 : YMR113W<br>2.1.2.1 : YLR058C<br>3.5.4.9 : YGR204W<br>1.5.1.5 : YBR084W<br>2.5.1.15 : YNL256W

Assignments for the pathway "folic acid biosynthesis" (rosetta data set). There are 48 possible assignments of genes to reactions.

Number	Normalized Score	Number of Pairs	Positive Pairs	Negative Pairs	Zero Pairs	Assignments
14	3.55	55	29	26	0	6.3.4.3 : YGR204W<br>6.3.2.17 : YOR241W<br>3.5.4.16 : YGR267C<br>4.1.2.25 : YNL256W<br>2.7.6.3 : YNL256W<br>1.5.1.3 : YOR236W<br>6.3.2.12 : YMR113W<br>2.1.2.1 : YLR058C<br>3.5.4.9 : YBR084W<br>1.5.1.5 : YBR084W<br>2.5.1.15 : YNL256W
15	3.55	55	29	26	0	6.3.4.3 : YBR084W<br>6.3.2.17 : YOR241W<br>3.5.4.16 : YGR267C<br>4.1.2.25 : YNL256W<br>2.7.6.3 : YNL256W<br>1.5.1.3 : YOR236W<br>6.3.2.12 : YMR113W<br>2.1.2.1 : YLR058C<br>3.5.4.9 : YBR084W<br>1.5.1.5 : YGR204W<br>2.5.1.15 : YNL256W
16	3.46	55	31	24	0	6.3.4.3 : YBR084W<br>6.3.2.17 : YMR113W<br>3.5.4.16 : YGR267C<br>4.1.2.25 : YNL256W<br>2.7.6.3 : YNL256W<br>1.5.1.3 : YOR236W<br>6.3.2.12 : YMR113W<br>2.1.2.1 : YLR058C<br>3.5.4.9 : YGR204W<br>1.5.1.5 : YBR084W<br>2.5.1.15 : YNL256W
17	3.46	55	31	24	0	6.3.4.3 : YGR204W<br>6.3.2.17 : YMR113W<br>3.5.4.16 : YGR267C<br>4.1.2.25 : YNL256W<br>2.7.6.3 : YNL256W<br>1.5.1.3 : YOR236W<br>6.3.2.12 : YMR113W<br>2.1.2.1 : YLR058C<br>3.5.4.9 : YBR084W<br>1.5.1.5 : YBR084W<br>2.5.1.15 : YNL256W

Assignments for the pathway "folic acid biosynthesis" (rosetta data set). There are 48 possible assignments of genes to reactions.

Number	Normalized Score	Number of Pairs	Positive Pairs	Negative Pairs	Zero Pairs	Assignments
18	3.46	55	31	24	0	6.3.4.3 : YBR084W<br>6.3.2.17 : YMR113W<br>3.5.4.16 : YGR267C<br>4.1.2.25 : YNL256W<br>2.7.6.3 : YNL256W<br>1.5.1.3 : YOR236W<br>6.3.2.12 : YMR113W<br>2.1.2.1 : YLR058C<br>3.5.4.9 : YBR084W<br>1.5.1.5 : YGR204W<br>2.5.1.15 : YNL256W
19	3.23	55	30	25	0	6.3.4.3 : YGR204W<br>6.3.2.17 : YKL132C<br>3.5.4.16 : YGR267C<br>4.1.2.25 : YNL256W<br>2.7.6.3 : YNL256W<br>1.5.1.3 : YOR236W<br>6.3.2.12 : YMR113W<br>2.1.2.1 : YLR058C<br>3.5.4.9 : YGR204W<br>1.5.1.5 : YBR084W<br>2.5.1.15 : YNL256W
20	3.23	55	30	25	0	6.3.4.3 : YGR204W<br>6.3.2.17 : YKL132C<br>3.5.4.16 : YGR267C<br>4.1.2.25 : YNL256W<br>2.7.6.3 : YNL256W<br>1.5.1.3 : YOR236W<br>6.3.2.12 : YMR113W<br>2.1.2.1 : YLR058C<br>3.5.4.9 : YBR084W<br>1.5.1.5 : YGR204W<br>2.5.1.15 : YNL256W
21	3.23	55	30	25	0	6.3.4.3 : YBR084W<br>6.3.2.17 : YKL132C<br>3.5.4.16 : YGR267C<br>4.1.2.25 : YNL256W<br>2.7.6.3 : YNL256W<br>1.5.1.3 : YOR236W<br>6.3.2.12 : YMR113W<br>2.1.2.1 : YLR058C<br>3.5.4.9 : YGR204W<br>1.5.1.5 : YGR204W<br>2.5.1.15 : YNL256W

Assignments for the pathway ”folic acid biosynthesis” (rosetta data set). There are 48 possible assignments of genes to reactions.

Number	Normalized Score	Number of Pairs	Positive Pairs	Negative Pairs	Zero Pairs	Assignments
22	3.17	55	30	25	0	6.3.4.3 : YGR204W<br>6.3.2.17 : YKL132C<br>3.5.4.16 : YGR267C<br>4.1.2.25 : YNL256W<br>2.7.6.3 : YNL256W<br>1.5.1.3 : YOR236W<br>6.3.2.12 : YMR113W<br>2.1.2.1 : YLR058C<br>3.5.4.9 : YGR204W<br>1.5.1.5 : YGR204W<br>2.5.1.15 : YNL256W
23	3.07	55	29	26	0	6.3.4.3 : YGR204W<br>6.3.2.17 : YOR241W<br>3.5.4.16 : YGR267C<br>4.1.2.25 : YNL256W<br>2.7.6.3 : YNL256W<br>1.5.1.3 : YOR236W<br>6.3.2.12 : YMR113W<br>2.1.2.1 : YBR263W<br>3.5.4.9 : YBR084W<br>1.5.1.5 : YBR084W<br>2.5.1.15 : YNL256W
24	3.07	55	29	26	0	6.3.4.3 : YBR084W<br>6.3.2.17 : YOR241W<br>3.5.4.16 : YGR267C<br>4.1.2.25 : YNL256W<br>2.7.6.3 : YNL256W<br>1.5.1.3 : YOR236W<br>6.3.2.12 : YMR113W<br>2.1.2.1 : YBR263W<br>3.5.4.9 : YGR204W<br>1.5.1.5 : YBR084W<br>2.5.1.15 : YNL256W
25	3.07	55	29	26	0	6.3.4.3 : YBR084W<br>6.3.2.17 : YOR241W<br>3.5.4.16 : YGR267C<br>4.1.2.25 : YNL256W<br>2.7.6.3 : YNL256W<br>1.5.1.3 : YOR236W<br>6.3.2.12 : YMR113W<br>2.1.2.1 : YBR263W<br>3.5.4.9 : YBR084W<br>1.5.1.5 : YGR204W<br>2.5.1.15 : YNL256W

Assignments for the pathway ”folic acid biosynthesis” (rosetta data set). There are 48 possible assignments of genes to reactions.

Number	Normalized Score	Number of Pairs	Positive Pairs	Negative Pairs	Zero Pairs	Assignments
26	2.97	55	29	26	0	6.3.4.3 : YGR204W<br>6.3.2.17 : YMR113W<br>3.5.4.16 : YGR267C<br>4.1.2.25 : YNL256W<br>2.7.6.3 : YNL256W<br>1.5.1.3 : YOR236W<br>6.3.2.12 : YMR113W<br>2.1.2.1 : YLR058C<br>3.5.4.9 : YGR204W<br>1.5.1.5 : YGR204W<br>2.5.1.15 : YNL256W
27	2.89	55	30	25	0	6.3.4.3 : YBR084W<br>6.3.2.17 : YMR113W<br>3.5.4.16 : YGR267C<br>4.1.2.25 : YNL256W<br>2.7.6.3 : YNL256W<br>1.5.1.3 : YOR236W<br>6.3.2.12 : YMR113W<br>2.1.2.1 : YBR263W<br>3.5.4.9 : YBR084W<br>1.5.1.5 : YGR204W<br>2.5.1.15 : YNL256W
28	2.89	55	30	25	0	6.3.4.3 : YGR204W<br>6.3.2.17 : YMR113W<br>3.5.4.16 : YGR267C<br>4.1.2.25 : YNL256W<br>2.7.6.3 : YNL256W<br>1.5.1.3 : YOR236W<br>6.3.2.12 : YMR113W<br>2.1.2.1 : YBR263W<br>3.5.4.9 : YBR084W<br>1.5.1.5 : YBR084W<br>2.5.1.15 : YNL256W
29	2.89	55	30	25	0	6.3.4.3 : YBR084W<br>6.3.2.17 : YMR113W<br>3.5.4.16 : YGR267C<br>4.1.2.25 : YNL256W<br>2.7.6.3 : YNL256W<br>1.5.1.3 : YOR236W<br>6.3.2.12 : YMR113W<br>2.1.2.1 : YBR263W<br>3.5.4.9 : YGR204W<br>1.5.1.5 : YBR084W<br>2.5.1.15 : YNL256W

Assignments for the pathway "folic acid biosynthesis" (rosetta data set). There are 48 possible assignments of genes to reactions.

Number	Normalized Score	Number of Pairs	Positive Pairs	Negative Pairs	Zero Pairs	Assignments
30	2.68	55	29	26	0	6.3.4.3 : YGR204W<br>6.3.2.17 : YMR113W<br>3.5.4.16 : YGR267C<br>4.1.2.25 : YNL256W<br>2.7.6.3 : YNL256W<br>1.5.1.3 : YOR236W<br>6.3.2.12 : YMR113W<br>2.1.2.1 : YLR058C<br>3.5.4.9 : YGR204W<br>1.5.1.5 : YBR084W<br>2.5.1.15 : YNL256W
31	2.68	55	29	26	0	6.3.4.3 : YGR204W<br>6.3.2.17 : YMR113W<br>3.5.4.16 : YGR267C<br>4.1.2.25 : YNL256W<br>2.7.6.3 : YNL256W<br>1.5.1.3 : YOR236W<br>6.3.2.12 : YMR113W<br>2.1.2.1 : YLR058C<br>3.5.4.9 : YBR084W<br>1.5.1.5 : YGR204W<br>2.5.1.15 : YNL256W
32	2.68	55	29	26	0	6.3.4.3 : YBR084W<br>6.3.2.17 : YMR113W<br>3.5.4.16 : YGR267C<br>4.1.2.25 : YNL256W<br>2.7.6.3 : YNL256W<br>1.5.1.3 : YOR236W<br>6.3.2.12 : YMR113W<br>2.1.2.1 : YLR058C<br>3.5.4.9 : YGR204W<br>1.5.1.5 : YGR204W<br>2.5.1.15 : YNL256W
33	2.62	55	28	27	0	6.3.4.3 : YGR204W<br>6.3.2.17 : YKL132C<br>3.5.4.16 : YGR267C<br>4.1.2.25 : YNL256W<br>2.7.6.3 : YNL256W<br>1.5.1.3 : YOR236W<br>6.3.2.12 : YMR113W<br>2.1.2.1 : YBR263W<br>3.5.4.9 : YBR084W<br>1.5.1.5 : YGR204W<br>2.5.1.15 : YNL256W

Assignments for the pathway "folic acid biosynthesis" (rosetta data set). There are 48 possible assignments of genes to reactions.

Number	Normalized Score	Number of Pairs	Positive Pairs	Negative Pairs	Zero Pairs	Assignments
34	2.62	55	28	27	0	6.3.4.3 : YGR204W<br>6.3.2.17 : YKL132C<br>3.5.4.16 : YGR267C<br>4.1.2.25 : YNL256W<br>2.7.6.3 : YNL256W<br>1.5.1.3 : YOR236W<br>6.3.2.12 : YMR113W<br>2.1.2.1 : YBR263W<br>3.5.4.9 : YGR204W<br>1.5.1.5 : YBR084W<br>2.5.1.15 : YNL256W
35	2.62	55	28	27	0	6.3.4.3 : YBR084W<br>6.3.2.17 : YKL132C<br>3.5.4.16 : YGR267C<br>4.1.2.25 : YNL256W<br>2.7.6.3 : YNL256W<br>1.5.1.3 : YOR236W<br>6.3.2.12 : YMR113W<br>2.1.2.1 : YBR263W<br>3.5.4.9 : YGR204W<br>1.5.1.5 : YGR204W<br>2.5.1.15 : YNL256W
36	2.44	55	27	28	0	6.3.4.3 : YGR204W<br>6.3.2.17 : YOR241W<br>3.5.4.16 : YGR267C<br>4.1.2.25 : YNL256W<br>2.7.6.3 : YNL256W<br>1.5.1.3 : YOR236W<br>6.3.2.12 : YMR113W<br>2.1.2.1 : YLR058C<br>3.5.4.9 : YBR084W<br>1.5.1.5 : YGR204W<br>2.5.1.15 : YNL256W
37	2.44	55	27	28	0	6.3.4.3 : YBR084W<br>6.3.2.17 : YOR241W<br>3.5.4.16 : YGR267C<br>4.1.2.25 : YNL256W<br>2.7.6.3 : YNL256W<br>1.5.1.3 : YOR236W<br>6.3.2.12 : YMR113W<br>2.1.2.1 : YLR058C<br>3.5.4.9 : YGR204W<br>1.5.1.5 : YGR204W<br>2.5.1.15 : YNL256W

Assignments for the pathway ”folic acid biosynthesis” (rosetta data set). There are 48 possible assignments of genes to reactions.

Number	Normalized Score	Number of Pairs	Positive Pairs	Negative Pairs	Zero Pairs	Assignments
38	2.44	55	27	28	0	6.3.4.3 : YGR204W<br>6.3.2.17 : YOR241W<br>3.5.4.16 : YGR267C<br>4.1.2.25 : YNL256W<br>2.7.6.3 : YNL256W<br>1.5.1.3 : YOR236W<br>6.3.2.12 : YMR113W<br>2.1.2.1 : YLR058C<br>3.5.4.9 : YGR204W<br>1.5.1.5 : YBR084W<br>2.5.1.15 : YNL256W
39	2.41	55	27	28	0	6.3.4.3 : YGR204W<br>6.3.2.17 : YKL132C<br>3.5.4.16 : YGR267C<br>4.1.2.25 : YNL256W<br>2.7.6.3 : YNL256W<br>1.5.1.3 : YOR236W<br>6.3.2.12 : YMR113W<br>2.1.2.1 : YBR263W<br>3.5.4.9 : YGR204W<br>1.5.1.5 : YGR204W<br>2.5.1.15 : YNL256W
40	2.41	55	27	28	0	6.3.4.3 : YGR204W<br>6.3.2.17 : YOR241W<br>3.5.4.16 : YGR267C<br>4.1.2.25 : YNL256W<br>2.7.6.3 : YNL256W<br>1.5.1.3 : YOR236W<br>6.3.2.12 : YMR113W<br>2.1.2.1 : YLR058C<br>3.5.4.9 : YGR204W<br>1.5.1.5 : YGR204W<br>2.5.1.15 : YNL256W
41	2.11	55	26	29	0	6.3.4.3 : YGR204W<br>6.3.2.17 : YMR113W<br>3.5.4.16 : YGR267C<br>4.1.2.25 : YNL256W<br>2.7.6.3 : YNL256W<br>1.5.1.3 : YOR236W<br>6.3.2.12 : YMR113W<br>2.1.2.1 : YBR263W<br>3.5.4.9 : YGR204W<br>1.5.1.5 : YGR204W<br>2.5.1.15 : YNL256W

Assignments for the pathway "folic acid biosynthesis" (rosetta data set). There are 48 possible assignments of genes to reactions.

Number	Normalized Score	Number of Pairs	Positive Pairs	Negative Pairs	Zero Pairs	Assignments
42	1.96	55	27	28	0	6.3.4.3 : YGR204W<br>6.3.2.17 : YMR113W<br>3.5.4.16 : YGR267C<br>4.1.2.25 : YNL256W<br>2.7.6.3 : YNL256W<br>1.5.1.3 : YOR236W<br>6.3.2.12 : YMR113W<br>2.1.2.1 : YBR263W<br>3.5.4.9 : YGR204W<br>1.5.1.5 : YBR084W<br>2.5.1.15 : YNL256W
43	1.96	55	27	28	0	6.3.4.3 : YGR204W<br>6.3.2.17 : YMR113W<br>3.5.4.16 : YGR267C<br>4.1.2.25 : YNL256W<br>2.7.6.3 : YNL256W<br>1.5.1.3 : YOR236W<br>6.3.2.12 : YMR113W<br>2.1.2.1 : YBR263W<br>3.5.4.9 : YBR084W<br>1.5.1.5 : YGR204W<br>2.5.1.15 : YNL256W
44	1.96	55	27	28	0	6.3.4.3 : YBR084W<br>6.3.2.17 : YMR113W<br>3.5.4.16 : YGR267C<br>4.1.2.25 : YNL256W<br>2.7.6.3 : YNL256W<br>1.5.1.3 : YOR236W<br>6.3.2.12 : YMR113W<br>2.1.2.1 : YBR263W<br>3.5.4.9 : YGR204W<br>1.5.1.5 : YGR204W<br>2.5.1.15 : YNL256W
45	1.83	55	26	29	0	6.3.4.3 : YGR204W<br>6.3.2.17 : YOR241W<br>3.5.4.16 : YGR267C<br>4.1.2.25 : YNL256W<br>2.7.6.3 : YNL256W<br>1.5.1.3 : YOR236W<br>6.3.2.12 : YMR113W<br>2.1.2.1 : YBR263W<br>3.5.4.9 : YGR204W<br>1.5.1.5 : YBR084W<br>2.5.1.15 : YNL256W

Assignments for the pathway ”folic acid biosynthesis” (rosetta data set). There are 48 possible assignments of genes to reactions.

Number	Normalized Score	Number of Pairs	Positive Pairs	Negative Pairs	Zero Pairs	Assignments
46	1.83	55	26	29	0	6.3.4.3 : YGR204W<br>6.3.2.17 : YOR241W<br>3.5.4.16 : YGR267C<br>4.1.2.25 : YNL256W<br>2.7.6.3 : YNL256W<br>1.5.1.3 : YOR236W<br>6.3.2.12 : YMR113W<br>2.1.2.1 : YBR263W<br>3.5.4.9 : YBR084W<br>1.5.1.5 : YGR204W<br>2.5.1.15 : YNL256W
47	1.83	55	26	29	0	6.3.4.3 : YBR084W<br>6.3.2.17 : YOR241W<br>3.5.4.16 : YGR267C<br>4.1.2.25 : YNL256W<br>2.7.6.3 : YNL256W<br>1.5.1.3 : YOR236W<br>6.3.2.12 : YMR113W<br>2.1.2.1 : YBR263W<br>3.5.4.9 : YGR204W<br>1.5.1.5 : YGR204W<br>2.5.1.15 : YNL256W
48	1.66	55	25	30	0	6.3.4.3 : YGR204W<br>6.3.2.17 : YOR241W<br>3.5.4.16 : YGR267C<br>4.1.2.25 : YNL256W<br>2.7.6.3 : YNL256W<br>1.5.1.3 : YOR236W<br>6.3.2.12 : YMR113W<br>2.1.2.1 : YBR263W<br>3.5.4.9 : YGR204W<br>1.5.1.5 : YGR204W<br>2.5.1.15 : YNL256W

Table 5: **Assignments for the pathway ”folic acid biosynthesis” (rosetta data set). There are 48 possible assignments of genes to reactions.**

Number	Normalized Score	Number of Pairs	Positive Pairs	Negative Pairs	Zero Pairs	Assignments
1	18.24	77	73	4	14	6.3.5.2 : YMR217W<br>1.1.1.205 : 4401<br>4.3.2.2 : YLR359W<br>6.3.4.4 : YNL220W<br>3.5.4.10 : YMR120C<br>2.1.2.3 : YMR120C<br>4.3.2.2 : YLR359W<br>6.3.2.6 : YAR015W<br>4.1.1.21 : YOR128C<br>6.3.3.1 : YGL234W<br>6.3.5.3 : YGR061C<br>2.1.2.2 : YDR408C<br>6.3.4.13 : YGL234W<br>2.4.2.14 : YMR300C
2	15.89	90	80	10	1	6.3.5.2 : YMR217W<br>1.1.1.205 : YAR073W<br>4.3.2.2 : YLR359W<br>6.3.4.4 : YNL220W<br>3.5.4.10 : YMR120C<br>2.1.2.3 : YMR120C<br>4.3.2.2 : YLR359W<br>6.3.2.6 : YAR015W<br>4.1.1.21 : YOR128C<br>6.3.3.1 : YGL234W<br>6.3.5.3 : YGR061C<br>2.1.2.2 : YDR408C<br>6.3.4.13 : YGL234W<br>2.4.2.14 : YMR300C
3	15.88	90	79	11	1	6.3.5.2 : YMR217W<br>1.1.1.205 : YHR216W<br>4.3.2.2 : YLR359W<br>6.3.4.4 : YNL220W<br>3.5.4.10 : YMR120C<br>2.1.2.3 : YMR120C<br>4.3.2.2 : YLR359W<br>6.3.2.6 : YAR015W<br>4.1.1.21 : YOR128C<br>6.3.3.1 : YGL234W<br>6.3.5.3 : YGR061C<br>2.1.2.2 : YDR408C<br>6.3.4.13 : YGL234W<br>2.4.2.14 : YMR300C

Assignments for the pathway "purine biosynthesis 2" (rosetta data set). There are 16 possible assignments of genes to reactions.

Number	Normalized Score	Number of Pairs	Positive Pairs	Negative Pairs	Zero Pairs	Assignments
4	15.88	90	79	11	1	6.3.5.2 : YMR217W<br>1.1.1.205 : YLR432W<br>4.3.2.2 : YLR359W<br>6.3.4.4 : YNL220W<br>3.5.4.10 : YMR120C<br>2.1.2.3 : YMR120C<br>4.3.2.2 : YLR359W<br>6.3.2.6 : YAR015W<br>4.1.1.21 : YOR128C<br>6.3.3.1 : YGL234W<br>6.3.5.3 : YGR061C<br>2.1.2.2 : YDR408C<br>6.3.4.13 : YGL234W<br>2.4.2.14 : YMR300C
5	15.08	77	70	7	14	6.3.5.2 : YMR217W<br>1.1.1.205 : 4401<br>4.3.2.2 : YLR359W<br>6.3.4.4 : YNL220W<br>3.5.4.10 : YMR120C<br>2.1.2.3 : YLR028C<br>4.3.2.2 : YLR359W<br>6.3.2.6 : YAR015W<br>4.1.1.21 : YOR128C<br>6.3.3.1 : YGL234W<br>6.3.5.3 : YGR061C<br>2.1.2.2 : YDR408C<br>6.3.4.13 : YGL234W<br>2.4.2.14 : YMR300C
6	15.08	77	70	7	14	6.3.5.2 : YMR217W<br>1.1.1.205 : 4401<br>4.3.2.2 : YLR359W<br>6.3.4.4 : YNL220W<br>3.5.4.10 : YLR028C<br>2.1.2.3 : YMR120C<br>4.3.2.2 : YLR359W<br>6.3.2.6 : YAR015W<br>4.1.1.21 : YOR128C<br>6.3.3.1 : YGL234W<br>6.3.5.3 : YGR061C<br>2.1.2.2 : YDR408C<br>6.3.4.13 : YGL234W<br>2.4.2.14 : YMR300C

Assignments for the pathway ”purine biosynthesis 2” (rosetta data set). There are 16 possible assignments of genes to reactions.

Number	Normalized Score	Number of Pairs	Positive Pairs	Negative Pairs	Zero Pairs	Assignments
7	13.23	90	78	12	1	6.3.5.2 : YMR217W<br>1.1.1.205 : YAR073W<br>4.3.2.2 : YLR359W<br>6.3.4.4 : YNL220W<br>3.5.4.10 : YMR120C<br>2.1.2.3 : YLR028C<br>4.3.2.2 : YLR359W<br>6.3.2.6 : YAR015W<br>4.1.1.21 : YOR128C<br>6.3.3.1 : YGL234W<br>6.3.5.3 : YGR061C<br>2.1.2.2 : YDR408C<br>6.3.4.13 : YGL234W<br>2.4.2.14 : YMR300C
8	13.23	90	78	12	1	6.3.5.2 : YMR217W<br>1.1.1.205 : YAR073W<br>4.3.2.2 : YLR359W<br>6.3.4.4 : YNL220W<br>3.5.4.10 : YLR028C<br>2.1.2.3 : YMR120C<br>4.3.2.2 : YLR359W<br>6.3.2.6 : YAR015W<br>4.1.1.21 : YOR128C<br>6.3.3.1 : YGL234W<br>6.3.5.3 : YGR061C<br>2.1.2.2 : YDR408C<br>6.3.4.13 : YGL234W<br>2.4.2.14 : YMR300C
9	13.22	90	76	14	1	6.3.5.2 : YMR217W<br>1.1.1.205 : YLR432W<br>4.3.2.2 : YLR359W<br>6.3.4.4 : YNL220W<br>3.5.4.10 : YMR120C<br>2.1.2.3 : YLR028C<br>4.3.2.2 : YLR359W<br>6.3.2.6 : YAR015W<br>4.1.1.21 : YOR128C<br>6.3.3.1 : YGL234W<br>6.3.5.3 : YGR061C<br>2.1.2.2 : YDR408C<br>6.3.4.13 : YGL234W<br>2.4.2.14 : YMR300C

Assignments for the pathway ”purine biosynthesis 2” (rosetta data set). There are 16 possible assignments of genes to reactions.

Number	Normalized Score	Number of Pairs	Positive Pairs	Negative Pairs	Zero Pairs	Assignments
10	13.22	90	76	14	1	6.3.5.2 : YMR217W<br>1.1.1.205 : YLR432W<br>4.3.2.2 : YLR359W<br>6.3.4.4 : YNL220W<br>3.5.4.10 : YLR028C<br>2.1.2.3 : YMR120C<br>4.3.2.2 : YLR359W<br>6.3.2.6 : YAR015W<br>4.1.1.21 : YOR128C<br>6.3.3.1 : YGL234W<br>6.3.5.3 : YGR061C<br>2.1.2.2 : YDR408C<br>6.3.4.13 : YGL234W<br>2.4.2.14 : YMR300C
11	13.22	90	77	13	1	6.3.5.2 : YMR217W<br>1.1.1.205 : YHR216W<br>4.3.2.2 : YLR359W<br>6.3.4.4 : YNL220W<br>3.5.4.10 : YMR120C<br>2.1.2.3 : YLR028C<br>4.3.2.2 : YLR359W<br>6.3.2.6 : YAR015W<br>4.1.1.21 : YOR128C<br>6.3.3.1 : YGL234W<br>6.3.5.3 : YGR061C<br>2.1.2.2 : YDR408C<br>6.3.4.13 : YGL234W<br>2.4.2.14 : YMR300C
12	13.22	90	77	13	1	6.3.5.2 : YMR217W<br>1.1.1.205 : YHR216W<br>4.3.2.2 : YLR359W<br>6.3.4.4 : YNL220W<br>3.5.4.10 : YLR028C<br>2.1.2.3 : YMR120C<br>4.3.2.2 : YLR359W<br>6.3.2.6 : YAR015W<br>4.1.1.21 : YOR128C<br>6.3.3.1 : YGL234W<br>6.3.5.3 : YGR061C<br>2.1.2.2 : YDR408C<br>6.3.4.13 : YGL234W<br>2.4.2.14 : YMR300C

Assignments for the pathway "purine biosynthesis 2" (rosetta data set). There are 16 possible assignments of genes to reactions.

Number	Normalized Score	Number of Pairs	Positive Pairs	Negative Pairs	Zero Pairs	Assignments
13	12.95	77	67	10	14	6.3.5.2 : YMR217W<br>1.1.1.205 : 4401<br>4.3.2.2 : YLR359W<br>6.3.4.4 : YNL220W<br>3.5.4.10 : YLR028C<br>2.1.2.3 : YLR028C<br>4.3.2.2 : YLR359W<br>6.3.2.6 : YAR015W<br>4.1.1.21 : YOR128C<br>6.3.3.1 : YGL234W<br>6.3.5.3 : YGR061C<br>2.1.2.2 : YDR408C<br>6.3.4.13 : YGL234W<br>2.4.2.14 : YMR300C
14	11.46	90	76	14	1	6.3.5.2 : YMR217W<br>1.1.1.205 : YAR073W<br>4.3.2.2 : YLR359W<br>6.3.4.4 : YNL220W<br>3.5.4.10 : YLR028C<br>2.1.2.3 : YLR028C<br>4.3.2.2 : YLR359W<br>6.3.2.6 : YAR015W<br>4.1.1.21 : YOR128C<br>6.3.3.1 : YGL234W<br>6.3.5.3 : YGR061C<br>2.1.2.2 : YDR408C<br>6.3.4.13 : YGL234W<br>2.4.2.14 : YMR300C
15	11.45	90	73	17	1	6.3.5.2 : YMR217W<br>1.1.1.205 : YLR432W<br>4.3.2.2 : YLR359W<br>6.3.4.4 : YNL220W<br>3.5.4.10 : YLR028C<br>2.1.2.3 : YLR028C<br>4.3.2.2 : YLR359W<br>6.3.2.6 : YAR015W<br>4.1.1.21 : YOR128C<br>6.3.3.1 : YGL234W<br>6.3.5.3 : YGR061C<br>2.1.2.2 : YDR408C<br>6.3.4.13 : YGL234W<br>2.4.2.14 : YMR300C

Assignments for the pathway "purine biosynthesis 2" (rosetta data set). There are 16 possible assignments of genes to reactions.

Number	Normalized Score	Number of Pairs	Positive Pairs	Negative Pairs	Zero Pairs	Assignments
16	11.44	90	75	15	1	6.3.5.2 : YMR217W<br>1.1.1.205 : YHR216W<br>4.3.2.2 : YLR359W<br>6.3.4.4 : YNL220W<br>3.5.4.10 : YLR028C<br>2.1.2.3 : YLR028C<br>4.3.2.2 : YLR359W<br>6.3.2.6 : YAR015W<br>4.1.1.21 : YOR128C<br>6.3.3.1 : YGL234W<br>6.3.5.3 : YGR061C<br>2.1.2.2 : YDR408C<br>6.3.4.13 : YGL234W<br>2.4.2.14 : YMR300C

Table 6: **Assignments for the pathway "purine biosynthesis 2" (rosetta data set). There are 16 possible assignments of genes to reactions.**

Number	Normalized Score	Number of Pairs	Positive Pairs	Negative Pairs	Zero Pairs	Assignments
1	1.93	43	23	20	2	2.7.4.6 : YKL067W<br>2.4.2.10 : YML106W<br>6.3.4.2 : YJR103W<br>2.7.4.6 : YKL067W<br>4.1.1.23 : YEL021W<br>2.4.2.10 : YML106W<br>1.3.3.1 : YKL216W<br>3.5.2.3 : YLR420W<br>2.1.3.2 : YJL130C<br>6.3.5.5 : YJL130C
2	1.71	43	21	22	2	2.7.4.6 : YKL067W<br>2.4.2.10 : YMR271C<br>6.3.4.2 : YJR103W<br>2.7.4.6 : YKL067W<br>4.1.1.23 : YEL021W<br>2.4.2.10 : YMR271C<br>1.3.3.1 : YKL216W<br>3.5.2.3 : YLR420W<br>2.1.3.2 : YJL130C<br>6.3.5.5 : YJL130C

Assignments for the pathway "de novo biosynthesis of pyrimidine ribonucleotides" (rosetta data set). There are 12 possible assignments of genes to reactions.

Number	Normalized Score	Number of Pairs	Positive Pairs	Negative Pairs	Zero Pairs	Assignments
3	1.58	43	23	20	2	2.7.4.6 : YKL067W<br>2.4.2.10 : YML106W<br>6.3.4.2 : YBL039C<br>2.7.4.6 : YKL067W<br>4.1.1.23 : YEL021W<br>2.4.2.10 : YML106W<br>1.3.3.1 : YKL216W<br>3.5.2.3 : YLR420W<br>2.1.3.2 : YJL130C<br>6.3.5.5 : YJL130C
4	1.26	43	21	22	2	2.7.4.6 : YKL067W<br>2.4.2.10 : YMR271C<br>6.3.4.2 : YJR103W<br>2.7.4.6 : YKL067W<br>4.1.1.23 : YEL021W<br>2.4.2.10 : YMR271C<br>1.3.3.1 : YKL216W<br>3.5.2.3 : YLR420W<br>2.1.3.2 : YJL130C<br>6.3.5.5 : YJR109C
5	1.00	43	21	22	2	2.7.4.6 : YKL067W<br>2.4.2.10 : YMR271C<br>6.3.4.2 : YJR103W<br>2.7.4.6 : YKL067W<br>4.1.1.23 : YEL021W<br>2.4.2.10 : YMR271C<br>1.3.3.1 : YKL216W<br>3.5.2.3 : YLR420W<br>2.1.3.2 : YJL130C<br>6.3.5.5 : YOR303W
6	0.28	43	19	24	2	2.7.4.6 : YKL067W<br>2.4.2.10 : YMR271C<br>6.3.4.2 : YBL039C<br>2.7.4.6 : YKL067W<br>4.1.1.23 : YEL021W<br>2.4.2.10 : YMR271C<br>1.3.3.1 : YKL216W<br>3.5.2.3 : YLR420W<br>2.1.3.2 : YJL130C<br>6.3.5.5 : YJL130C

Assignments for the pathway ”de novo biosynthesis of pyrimidine ribonucleotides” (rosetta data set). There are 12 possible assignments of genes to reactions.

Number	Normalized Score	Number of Pairs	Positive Pairs	Negative Pairs	Zero Pairs	Assignments
7	-0.05	43	19	24	2	2.7.4.6 : YKL067W<br>2.4.2.10 : YMR271C<br>6.3.4.2 : YBL039C<br>2.7.4.6 : YKL067W<br>4.1.1.23 : YEL021W<br>2.4.2.10 : YMR271C<br>1.3.3.1 : YKL216W<br>3.5.2.3 : YLR420W<br>2.1.3.2 : YJL130C<br>6.3.5.5 : YJR109C
8	-0.16	43	19	24	2	2.7.4.6 : YKL067W<br>2.4.2.10 : YML106W<br>6.3.4.2 : YJR103W<br>2.7.4.6 : YKL067W<br>4.1.1.23 : YEL021W<br>2.4.2.10 : YML106W<br>1.3.3.1 : YKL216W<br>3.5.2.3 : YLR420W<br>2.1.3.2 : YJL130C<br>6.3.5.5 : YOR303W
9	-0.27	43	19	24	2	2.7.4.6 : YKL067W<br>2.4.2.10 : YML106W<br>6.3.4.2 : YJR103W<br>2.7.4.6 : YKL067W<br>4.1.1.23 : YEL021W<br>2.4.2.10 : YML106W<br>1.3.3.1 : YKL216W<br>3.5.2.3 : YLR420W<br>2.1.3.2 : YJL130C<br>6.3.5.5 : YJR109C
10	-0.36	43	19	24	2	2.7.4.6 : YKL067W<br>2.4.2.10 : YMR271C<br>6.3.4.2 : YBL039C<br>2.7.4.6 : YKL067W<br>4.1.1.23 : YEL021W<br>2.4.2.10 : YMR271C<br>1.3.3.1 : YKL216W<br>3.5.2.3 : YLR420W<br>2.1.3.2 : YJL130C<br>6.3.5.5 : YOR303W

Assignments for the pathway ”de novo biosynthesis of pyrimidine ribonucleotides” (rosetta data set). There are 12 possible assignments of genes to reactions.

Number	Normalized Score	Number of Pairs	Positive Pairs	Negative Pairs	Zero Pairs	Assignments
11	-0.44	43	19	24	2	2.7.4.6 : YKL067W<br>2.4.2.10 : YML106W<br>6.3.4.2 : YBL039C<br>2.7.4.6 : YKL067W<br>4.1.1.23 : YEL021W<br>2.4.2.10 : YML106W<br>1.3.3.1 : YKL216W<br>3.5.2.3 : YLR420W<br>2.1.3.2 : YJL130C<br>6.3.5.5 : YOR303W
12	-0.50	43	19	24	2	2.7.4.6 : YKL067W<br>2.4.2.10 : YML106W<br>6.3.4.2 : YBL039C<br>2.7.4.6 : YKL067W<br>4.1.1.23 : YEL021W<br>2.4.2.10 : YML106W<br>1.3.3.1 : YKL216W<br>3.5.2.3 : YLR420W<br>2.1.3.2 : YJL130C<br>6.3.5.5 : YJR109C

Table 7: **Assignments for the pathway "de novo biosynthesis of pyrimidine ribonucleotides" (rosetta data set). There are 12 possible assignments of genes to reactions.**

Number	Normalized Score	Number of Pairs	Positive Pairs	Negative Pairs	Zero Pairs	Assignments
1	32.66	6	6	0	4	4.2.1.16 : 570<br>4.1.3.18 : YMR108W<br>1.1.1.86 : YLR355C<br>2.6.1.42 : YHR208W<br>4.2.1.9 : YJR016C
2	31.33	10	10	0	0	4.2.1.16 : YER086W<br>4.1.3.18 : YMR108W<br>1.1.1.86 : YLR355C<br>2.6.1.42 : YHR208W<br>4.2.1.9 : YJR016C
3	28.87	10	10	0	0	4.2.1.16 : YKL218C<br>4.1.3.18 : YMR108W<br>1.1.1.86 : YLR355C<br>2.6.1.42 : YHR208W<br>4.2.1.9 : YJR016C
4	27.91	10	10	0	0	4.2.1.16 : YER086W<br>4.1.3.18 : YMR108W<br>1.1.1.86 : YLR355C<br>2.6.1.42 : YJR148W<br>4.2.1.9 : YJR016C

Assignments for the pathway "isoleucine biosynthesis I" (rosetta data set). There are 12 possible assignments of genes to reactions.

Number	Normalized Score	Number of Pairs	Positive Pairs	Negative Pairs	Zero Pairs	Assignments
5	27.84	6	6	0	4	4.2.1.16 : 570<br>4.1.3.18 : YMR108W<br>1.1.1.86 : YLR355C<br>2.6.1.42 : YJR148W<br>4.2.1.9 : YJR016C
6	27.67	10	10	0	0	4.2.1.16 : YER086W<br>4.1.3.18 : YCL009C<br>1.1.1.86 : YLR355C<br>2.6.1.42 : YHR208W<br>4.2.1.9 : YJR016C
7	27.10	6	6	0	4	4.2.1.16 : 570<br>4.1.3.18 : YCL009C<br>1.1.1.86 : YLR355C<br>2.6.1.42 : YHR208W<br>4.2.1.9 : YJR016C
8	26.19	10	10	0	0	4.2.1.16 : YKL218C<br>4.1.3.18 : YMR108W<br>1.1.1.86 : YLR355C<br>2.6.1.42 : YJR148W<br>4.2.1.9 : YJR016C
9	25.04	10	10	0	0	4.2.1.16 : YER086W<br>4.1.3.18 : YCL009C<br>1.1.1.86 : YLR355C<br>2.6.1.42 : YJR148W<br>4.2.1.9 : YJR016C
10	24.58	10	10	0	0	4.2.1.16 : YKL218C<br>4.1.3.18 : YCL009C<br>1.1.1.86 : YLR355C<br>2.6.1.42 : YHR208W<br>4.2.1.9 : YJR016C
11	23.59	6	6	0	4	4.2.1.16 : 570<br>4.1.3.18 : YCL009C<br>1.1.1.86 : YLR355C<br>2.6.1.42 : YJR148W<br>4.2.1.9 : YJR016C
12	22.68	10	10	0	0	4.2.1.16 : YKL218C<br>4.1.3.18 : YCL009C<br>1.1.1.86 : YLR355C<br>2.6.1.42 : YJR148W<br>4.2.1.9 : YJR016C

Table 8: **Assignments for the pathway "isoleucine biosynthesis I" (rosetta data set). There are 12 possible assignments of genes to reactions.**

Number	Normalized Score	Number of Pairs	Positive Pairs	Negative Pairs	Zero Pairs	Assignments
1	12.00	10	9	1	0	1.1.1.44 : YHR183W<br>5.3.1.6 : YOR095C<br>5.1.3.1 : YJL121C<br>2.2.1.1 : YPR074C<br>2.2.1.2 : YLR354C
2	7.27	10	8	2	0	1.1.1.44 : YGR256W<br>5.3.1.6 : YOR095C<br>5.1.3.1 : YJL121C<br>2.2.1.1 : YPR074C<br>2.2.1.2 : YLR354C
3	5.58	10	8	2	0	1.1.1.44 : YHR183W<br>5.3.1.6 : YOR095C<br>5.1.3.1 : YJL121C<br>2.2.1.1 : YBR117C<br>2.2.1.2 : YLR354C
4	4.66	10	6	4	0	1.1.1.44 : YHR183W<br>5.3.1.6 : YOR095C<br>5.1.3.1 : YJL121C<br>2.2.1.1 : YPR074C<br>2.2.1.2 : YGR043C
5	2.25	10	7	3	0	1.1.1.44 : YGR256W<br>5.3.1.6 : YOR095C<br>5.1.3.1 : YJL121C<br>2.2.1.1 : YBR117C<br>2.2.1.2 : YLR354C
6	2.11	10	6	4	0	1.1.1.44 : YGR256W<br>5.3.1.6 : YOR095C<br>5.1.3.1 : YJL121C<br>2.2.1.1 : YPR074C<br>2.2.1.2 : YGR043C
7	0.58	10	6	4	0	1.1.1.44 : YHR183W<br>5.3.1.6 : YOR095C<br>5.1.3.1 : YJL121C<br>2.2.1.1 : YBR117C<br>2.2.1.2 : YGR043C
8	-0.57	10	6	4	0	1.1.1.44 : YGR256W<br>5.3.1.6 : YOR095C<br>5.1.3.1 : YJL121C<br>2.2.1.1 : YBR117C<br>2.2.1.2 : YGR043C

Table 9: **Assignments for the pathway "non-oxidative branch of the pentose phosphate pathway" (rosetta data set). There are 8 possible assignments of genes to reactions.**

Number	Normalized Score	Number of Pairs	Positive Pairs	Negative Pairs	Zero Pairs	Assignments
1	28.64	6	6	0	0	3.1.3.12 : YDR074W<br>2.4.1.15 : YBR126C<br>2.7.7.9 : YKL035W<br>5.4.2.2 : YMR105C
2	27.40	6	6	0	0	3.1.3.12 : YDR074W<br>2.4.1.15 : YML100W<br>2.7.7.9 : YKL035W<br>5.4.2.2 : YMR105C
3	15.65	6	6	0	0	3.1.3.12 : YDR074W<br>2.4.1.15 : YBR126C<br>2.7.7.9 : YKL035W<br>5.4.2.2 : YKL127W
4	13.02	6	5	1	0	3.1.3.12 : YDR074W<br>2.4.1.15 : YML100W<br>2.7.7.9 : YKL035W<br>5.4.2.2 : YKL127W
5	12.16	6	4	2	0	3.1.3.12 : YDR074W<br>2.4.1.15 : YML100W<br>2.7.7.9 : YHL012W<br>5.4.2.2 : YMR105C
6	10.16	6	3	3	0	3.1.3.12 : YDR074W<br>2.4.1.15 : YBR126C<br>2.7.7.9 : YHL012W<br>5.4.2.2 : YMR105C
7	2.23	6	4	2	0	3.1.3.12 : YDR074W<br>2.4.1.15 : YML100W<br>2.7.7.9 : YHL012W<br>5.4.2.2 : YKL127W
8	1.63	6	4	2	0	3.1.3.12 : YDR074W<br>2.4.1.15 : YBR126C<br>2.7.7.9 : YHL012W<br>5.4.2.2 : YKL127W

Table 10: **Assignments for the pathway ”trehalose anabolism” (rosetta data set). There are 8 possible assignments of genes to reactions.**

Number	Normalized Score	Number of Pairs	Positive Pairs	Negative Pairs	Zero Pairs	Assignments
1	10.45	3	3	0	0	1.1.1.49 : YNL241C<br>1.1.1.44 : YGR256W<br>3.1.1.31 : YNR034W
2	8.52	3	3	0	0	1.1.1.49 : YNL241C<br>1.1.1.44 : YGR256W<br>3.1.1.31 : YGR248W
3	7.70	3	3	0	0	1.1.1.49 : YNL241C<br>1.1.1.44 : YHR183W<br>3.1.1.31 : YNR034W

Assignments for the pathway ”oxidative branch of the pentose phosphate pathway” (rosetta data set). There are 6 possible assignments of genes to reactions.

Number	Normalized Score	Number of Pairs	Positive Pairs	Negative Pairs	Zero Pairs	Assignments
4	5.37	3	2	1	0	1.1.1.49 : YNL241C<br>1.1.1.44 : YGR256W<br>3.1.1.31 : YHR163W
5	4.97	3	2	1	0	1.1.1.49 : YNL241C<br>1.1.1.44 : YHR183W<br>3.1.1.31 : YHR163W
6	1.53	3	2	1	0	1.1.1.49 : YNL241C<br>1.1.1.44 : YHR183W<br>3.1.1.31 : YGR248W

Table 11: **Assignments for the pathway "oxidative branch of the pentose phosphate pathway" (rosetta data set). There are 6 possible assignments of genes to reactions.**

Number	Normalized Score	Number of Pairs	Positive Pairs	Negative Pairs	Zero Pairs	Assignments
1	9.91	3	3	0	0	3.6.1.1 : YBR011C<br>5.1.3.2 : YBR019C<br>2.7.7.9 : YKL035W
2	6.43	3	3	0	0	3.6.1.1 : YMR267W<br>5.1.3.2 : YBR019C<br>2.7.7.9 : YKL035W
3	-5.72	3	1	2	0	3.6.1.1 : YMR267W<br>5.1.3.2 : YBR019C<br>2.7.7.9 : YHL012W
4	-6.41	3	1	2	0	3.6.1.1 : YBR011C<br>5.1.3.2 : YBR019C<br>2.7.7.9 : YHL012W

Table 12: **Assignments for the pathway "UDP-glucose conversion" (rosetta data set). There are 4 possible assignments of genes to reactions.**

Number	Normalized Score	Number of Pairs	Positive Pairs	Negative Pairs	Zero Pairs	Assignments
1	25.06	1	1	0	0	2.6.1.1 : YLR027C<br>6.4.1.1 : YBR218C
2	17.11	1	1	0	0	2.6.1.1 : YLR027C<br>6.4.1.1 : YGL062W
3	13.08	1	1	0	0	2.6.1.1 : YKL106W<br>6.4.1.1 : YBR218C
4	6.66	1	1	0	0	2.6.1.1 : YKL106W<br>6.4.1.1 : YGL062W

Table 13: **Assignments for the pathway "aspartate biosynthesis II" (rosetta data set). There are 4 possible assignments of genes to reactions.**

Number	Normalized Score	Number of Pairs	Positive Pairs	Negative Pairs	Zero Pairs	Assignments
1	27.27	1	1	0	0	6.3.5.4 : YPR145W<br>2.6.1.1 : YLR027C
2	24.28	1	1	0	0	6.3.5.4 : YGR124W<br>2.6.1.1 : YLR027C
3	12.50	1	1	0	0	6.3.5.4 : YPR145W<br>2.6.1.1 : YKL106W
4	10.60	1	1	0	0	6.3.5.4 : YGR124W<br>2.6.1.1 : YKL106W

Table 14: **Assignments for the pathway "asparagine biosynthesis I" (rosetta data set). There are 4 possible assignments of genes to reactions.**

Number	Normalized Score	Number of Pairs	Positive Pairs	Negative Pairs	Zero Pairs	Assignments
1	9.54	6	6	0	0	2.7.7.9 : YKL035W<br>5.1.3.2 : YBR019C<br>2.7.7.12 : YBR018C<br>2.7.1.6 : YDR009W
2	5.35	6	6	0	0	2.7.7.9 : YKL035W<br>5.1.3.2 : YBR019C<br>2.7.7.12 : YBR018C<br>2.7.1.6 : YBR020W
3	-2.36	6	4	2	0	2.7.7.9 : YHL012W<br>5.1.3.2 : YBR019C<br>2.7.7.12 : YBR018C<br>2.7.1.6 : YBR020W
4	-3.34	6	3	3	0	2.7.7.9 : YHL012W<br>5.1.3.2 : YBR019C<br>2.7.7.12 : YBR018C<br>2.7.1.6 : YDR009W

Table 15: **Assignments for the pathway "galactose metabolism" (rosetta data set). There are 4 possible assignments of genes to reactions.**

Number	Normalized Score	Number of Pairs	Positive Pairs	Negative Pairs	Zero Pairs	Assignments
1	32.66	6	6	0	0	4.1.3.18 : YMR108W<br>1.1.1.86 : YLR355C<br>2.6.1.42 : YHR208W<br>4.2.1.9 : YJR016C
2	27.84	6	6	0	0	4.1.3.18 : YMR108W<br>1.1.1.86 : YLR355C<br>2.6.1.42 : YJR148W<br>4.2.1.9 : YJR016C

Assignments for the pathway "valine biosynthesis" (rosetta data set). There are 4 possible assignments of genes to reactions.

Number	Normalized Score	Number of Pairs	Positive Pairs	Negative Pairs	Zero Pairs	Assignments
3	27.10	6	6	0	0	4.1.3.18 : YCL009C<br>1.1.1.86 : YLR355C<br>2.6.1.42 : YHR208W<br>4.2.1.9 : YJR016C
4	23.59	6	6	0	0	4.1.3.18 : YCL009C<br>1.1.1.86 : YLR355C<br>2.6.1.42 : YJR148W<br>4.2.1.9 : YJR016C

Table 16: **Assignments for the pathway "valine biosynthesis" (rosetta data set). There are 4 possible assignments of genes to reactions.**

Number	Normalized Score	Number of Pairs	Positive Pairs	Negative Pairs	Zero Pairs	Assignments
1	26.89	3	3	0	3	4.1.3.12 : YNL104C<br>4.2.1.33 : YGL009C<br>2.6.1.42 : YHR208W<br>1.1.1.85 : 612
2	25.45	3	3	0	3	4.1.3.12 : YOR108W<br>4.2.1.33 : YGL009C<br>2.6.1.42 : YHR208W<br>1.1.1.85 : 612
3	20.02	3	3	0	3	4.1.3.12 : YNL104C<br>4.2.1.33 : YGL009C<br>2.6.1.42 : YJR148W<br>1.1.1.85 : 612
4	18.80	3	3	0	3	4.1.3.12 : YOR108W<br>4.2.1.33 : YGL009C<br>2.6.1.42 : YJR148W<br>1.1.1.85 : 612

Table 17: **Assignments for the pathway "leucine biosynthesis" (rosetta data set). There are 4 possible assignments of genes to reactions.**

Number	Normalized Score	Number of Pairs	Positive Pairs	Negative Pairs	Zero Pairs	Assignments
1	8.28	1	1	0	0	1.11.1.6 : YGR088W<br>1.15.1.1 : YHR008C
2	1.92	1	1	0	0	1.11.1.6 : YGR088W<br>1.15.1.1 : YJR104C
3	-1.62	1	0	1	0	1.11.1.6 : YDR256C<br>1.15.1.1 : YJR104C
4	-2.55	1	0	1	0	1.11.1.6 : YDR256C<br>1.15.1.1 : YHR008C

Table 18: **Assignments for the pathway "removal of superoxide radicals" (rosetta data set). There are 4 possible assignments of genes to reactions.**

Number	Normalized Score	Number of Pairs	Positive Pairs	Negative Pairs	Zero Pairs	Assignments
1	27.64	36	36	0	0	2.3.1.35 : YMR062C<br>6.3.5.5 : YJR109C<br>2.3.1.1 : YJL071W<br>2.7.2.8 : YER069W<br>1.2.1.38 : YER069W<br>2.6.1.11 : YOL140W<br>2.1.3.3 : YJL088W<br>4.3.2.1 : YHR018C<br>6.3.4.5 : YOL058W
2	26.46	36	36	0	0	2.3.1.35 : YMR062C<br>6.3.5.5 : YOR303W<br>2.3.1.1 : YJL071W<br>2.7.2.8 : YER069W<br>1.2.1.38 : YER069W<br>2.6.1.11 : YOL140W<br>2.1.3.3 : YJL088W<br>4.3.2.1 : YHR018C<br>6.3.4.5 : YOL058W
3	22.22	36	36	0	0	2.3.1.35 : YMR062C<br>6.3.5.5 : YJL130C<br>2.3.1.1 : YJL071W<br>2.7.2.8 : YER069W<br>1.2.1.38 : YER069W<br>2.6.1.11 : YOL140W<br>2.1.3.3 : YJL088W<br>4.3.2.1 : YHR018C<br>6.3.4.5 : YOL058W

Table 19: **Assignments for the pathway "arginine biosynthesis, Bacillus subtilis" (rosetta data set). There are 3 possible assignments of genes to reactions.**

Number	Normalized Score	Number of Pairs	Positive Pairs	Negative Pairs	Zero Pairs	Assignments
1	18.20	10	6	4	0	4.1.3.27 : YER090W<br>2.4.2.18 : YDR354W<br>5.3.1.24 : YDR007W<br>4.1.1.48 : YKL211C<br>4.2.1.20 : YGL026C
2	17.37	10	6	4	0	4.1.3.27 : YKL211C<br>2.4.2.18 : YDR354W<br>5.3.1.24 : YDR007W<br>4.1.1.48 : YKL211C<br>4.2.1.20 : YGL026C

Table 20: **Assignments for the pathway "tryptophan biosynthesis" (rosetta data set). There are 2 possible assignments of genes to reactions.**

Number	Normalized Score	Number of Pairs	Positive Pairs	Negative Pairs	Zero Pairs	Assignments
1	25.75	1	1	0	0	2.2.1.1 : YPR074C<br>5.1.3.1 : YJL121C
2	0.81	1	1	0	0	2.2.1.1 : YBR117C<br>5.1.3.1 : YJL121C

Table 21: **Assignments for the pathway "pentose phosphate pathway, Mycoplasma pneumoniae" (rosetta data set). There are 2 possible assignments of genes to reactions.**

Number	Normalized Score	Number of Pairs	Positive Pairs	Negative Pairs	Zero Pairs	Assignments
1	17.63	1	1	0	2	1.2.4.1 : YER178W<br>2.3.1.12 : YNL071W<br>1.8.1.4 : 1891
2	5.23	1	1	0	2	1.2.4.1 : YBR221C<br>2.3.1.12 : YNL071W<br>1.8.1.4 : 1891

Table 22: **Assignments for the pathway "pyruvate dehydrogenase" (rosetta data set). There are 2 possible assignments of genes to reactions.**

Number	Normalized Score	Number of Pairs	Positive Pairs	Negative Pairs	Zero Pairs	Assignments
1	32.06	1	1	0	0	2.1.2.10 : YDR019C<br>1.4.4.2 : YMR189W
2	32.06	1	1	0	0	2.1.2.10 : YMR189W<br>1.4.4.2 : YMR189W

Table 23: **Assignments for the pathway "glycine degradation III" (rosetta data set). There are 2 possible assignments of genes to reactions.**

Number	Normalized Score	Number of Pairs	Positive Pairs	Negative Pairs	Zero Pairs	Assignments
1	9.18	6	6	0	0	4.2.1.22 : YGR155W<br>3.1.3.3 : YGR208W<br>2.6.1.52 : YOR184W<br>1.1.1.95 : YIL074C
2	7.01	6	4	2	0	4.2.1.22 : YGR155W<br>3.1.3.3 : YGR208W<br>2.6.1.52 : YOR184W<br>1.1.1.95 : YER081W

Table 24: **Assignments for the pathway "cysteine biosynthesis II" (rosetta data set). There are 2 possible assignments of genes to reactions.**

Number	Normalized Score	Number of Pairs	Positive Pairs	Negative Pairs	Zero Pairs	Assignments
1	7.49	1	1	0	0	3.1.2.6 : YDR272W<br>4.4.1.5 : YML004C
2	3.22	1	1	0	0	3.1.2.6 : YOR040W<br>4.4.1.5 : YML004C

Table 25: **Assignments for the pathway "methylglyoxal degradation" (rosetta data set). There are 2 possible assignments of genes to reactions.**

Number	Normalized Score	Number of Pairs	Positive Pairs	Negative Pairs	Zero Pairs	Assignments
1	-0.68	1	0	1	0	2.1.2.1 : YLR058C<br>3.1.3.3 : YGR208W
2	-1.75	1	0	1	0	2.1.2.1 : YBR263W<br>3.1.3.3 : YGR208W

Table 26: **Assignments for the pathway "glycine biosynthesis I" (rosetta data set). There are 2 possible assignments of genes to reactions.**

Number	Normalized Score	Number of Pairs	Positive Pairs	Negative Pairs	Zero Pairs	Assignments
1	6.69	1	1	0	2	4.4.1.8 : 1971<br>4.4.1.1 : YAL012W<br>4.2.1.22 : YGR155W
2	5.58	3	3	0	0	4.4.1.8 : YGL184C<br>4.4.1.1 : YAL012W<br>4.2.1.22 : YGR155W

Table 27: **Assignments for the pathway "homocysteine and cysteine interconversion" (rosetta data set). There are 2 possible assignments of genes to reactions.**

Number	Normalized Score	Number of Pairs	Positive Pairs	Negative Pairs	Zero Pairs	Assignments
1	3.43	3	1	2	0	2.5.1.22 : YLR146C<br>2.5.1.16 : YPR069C<br>4.1.1.17 : YKL184W
2	-3.00	3	0	3	0	2.5.1.22 : YLR146C<br>2.5.1.16 : YLR146C<br>4.1.1.17 : YKL184W

Table 28: **Assignments for the pathway "ornithine spermine biosynthesis" (rosetta data set). There are 2 possible assignments of genes to reactions.**

Number	Normalized Score	Number of Pairs	Positive Pairs	Negative Pairs	Zero Pairs	Assignments
1	0.34	1	1	0	0	1.4.1.4 : YOR375C<br>1.4.1.13 : YDL171C
2	-0.72	1	0	1	0	1.4.1.4 : YAL062W<br>1.4.1.13 : YDL171C

Table 29: **Assignments for the pathway "glutamate biosynthesis I" (rosetta data set). There are 2 possible assignments of genes to reactions.**

Number	Normalized Score	Number of Pairs	Positive Pairs	Negative Pairs	Zero Pairs	Assignments
1	32.78	21	21	0	0	4.1.2.15 : YBR249C<br>4.6.1.3 : YDR127W<br>4.2.1.10 : YDR127W<br>1.1.1.25 : YDR127W<br>2.7.1.71 : YDR127W<br>2.5.1.19 : YDR127W<br>4.6.1.4 : YGL148W
2	31.34	21	21	0	0	4.1.2.15 : YDR035W<br>4.6.1.3 : YDR127W<br>4.2.1.10 : YDR127W<br>1.1.1.25 : YDR127W<br>2.7.1.71 : YDR127W<br>2.5.1.19 : YDR127W<br>4.6.1.4 : YGL148W

Table 30: **Assignments for the pathway "chorismate biosynthesis" (rosetta data set). There are 2 possible assignments of genes to reactions.**

Number	Normalized Score	Number of Pairs	Positive Pairs	Negative Pairs	Zero Pairs	Assignments
1	12.04	3	3	0	0	1.1.1.95 : YIL074C<br>3.1.3.3 : YGR208W<br>2.6.1.52 : YOR184W
2	9.68	3	2	1	0	1.1.1.95 : YER081W<br>3.1.3.3 : YGR208W<br>2.6.1.52 : YOR184W

Table 31: **Assignments for the pathway "serine biosynthesis" (rosetta data set). There are 2 possible assignments of genes to reactions.**

Number	Normalized Score	Number of Pairs	Positive Pairs	Negative Pairs	Zero Pairs	Assignments
1	2.93	3	2	1	0	5.4.99.5 : YPR060C<br>4.2.1.51 : YNL316C<br>2.6.1.57 : YGL202W
2	1.11	3	2	1	0	5.4.99.5 : YPR060C<br>4.2.1.51 : YNL316C<br>2.6.1.57 : YHR137W

Table 32: **Assignments for the pathway "phenylalanine biosynthesis I" (rosetta data set). There are 2 possible assignments of genes to reactions.**

Number	Normalized Score	Number of Pairs	Positive Pairs	Negative Pairs	Zero Pairs	Assignments
1	1.99	3	1	2	0	5.4.99.5 : YPR060C<br>1.3.1.13 : YBR166C<br>2.6.1.57 : YGL202W
2	1.55	3	1	2	0	5.4.99.5 : YPR060C<br>1.3.1.13 : YBR166C<br>2.6.1.57 : YHR137W

Table 33: **Assignments for the pathway "tyrosine biosynthesis I" (rosetta data set). There are 2 possible assignments of genes to reactions.**

Number	Normalized Score	Number of Pairs	Positive Pairs	Negative Pairs	Zero Pairs	Assignments
1	5.44	1	1	0	2	2.2.1.1 : YPR074C<br>5.3.1.6 : YOR095C<br>2.7.1.15 : 667
2	-3.48	1	0	1	2	2.2.1.1 : YBR117C<br>5.3.1.6 : YOR095C<br>2.7.1.15 : 667

Table 34: **Assignments for the pathway "ribose degradation" (rosetta data set). There are 2 possible assignments of genes to reactions.**

Number	Normalized Score	Number of Pairs	Positive Pairs	Negative Pairs	Zero Pairs	Assignments
1	29.96	1	1	0	0	3.1.3.12 : YDR074W<br>2.4.1.15 : YBR126C
2	28.06	1	1	0	0	3.1.3.12 : YDR074W<br>2.4.1.15 : YML100W

Table 35: **Assignments for the pathway "trehalose biosynthesis" (rosetta data set). There are 2 possible assignments of genes to reactions.**

Number	Normalized Score	Number of Pairs	Positive Pairs	Negative Pairs	Zero Pairs	Assignments
1	18.36	1	1	0	0	2.5.1.6 : YDR502C<br>2.1.1.14 : YER091C
2	17.24	1	1	0	0	2.5.1.6 : YLR180W<br>2.1.1.14 : YER091C

Table 36: **Assignments for the pathway "methionine and S-adenosylmethionine synthesis" (rosetta data set). There are 2 possible assignments of genes to reactions.**

Number	Normalized Score	Number of Pairs	Positive Pairs	Negative Pairs	Zero Pairs	Assignments
1	11.20	3	3	0	0	1.2.1.16 : YBR006W<br>4.1.1.15 : YMR250W<br>2.6.1.19 : YGR019W

Table 37: **Assignments for the pathway "glutamate degradation I" (rosetta data set). There are 1 possible assignments of genes to reactions.**

Number	Normalized Score	Number of Pairs	Positive Pairs	Negative Pairs	Zero Pairs	Assignments
1	11.64	3	3	0	3	2.3.1.47 : N/A<br>2.6.1.62 : YNR058W<br>2.8.1.6 : YGR286C<br>6.3.3.3 : YNR057C

Table 38: **Assignments for the pathway "biotin biosynthesis I" (rosetta data set). There are 1 possible assignments of genes to reactions.**

Number	Normalized Score	Number of Pairs	Positive Pairs	Negative Pairs	Zero Pairs	Assignments
1	23.14	10	10	0	0	2.3.1.35 : YMR062C<br>2.6.1.11 : YOL140W<br>1.2.1.38 : YER069W<br>2.7.2.8 : YER069W<br>2.3.1.1 : YJL071W

Table 39: **Assignments for the pathway "glutamate degradation VIII" (rosetta data set). There are 1 possible assignments of genes to reactions.**

Number	Normalized Score	Number of Pairs	Positive Pairs	Negative Pairs	Zero Pairs	Assignments
1	19.35	1	1	0	0	4.2.99.2 : YCR053W<br>2.7.1.39 : YHR025W

Table 40: **Assignments for the pathway "threonine biosynthesis from homoserine" (rosetta data set). There are 1 possible assignments of genes to reactions.**

Number	Normalized Score	Number of Pairs	Positive Pairs	Negative Pairs	Zero Pairs	Assignments
1	6.69	1	1	0	0	4.4.1.1 : YAL012W<br>4.2.1.22 : YGR155W

Table 41: **Assignments for the pathway "homocysteine degradation I" (rosetta data set). There are 1 possible assignments of genes to reactions.**

Number	Normalized Score	Number of Pairs	Positive Pairs	Negative Pairs	Zero Pairs	Assignments
1	7.27	1	1	0	0	2.3.1.31 : YNL277W<br>2.1.1.14 : YER091C

Table 42: **Assignments for the pathway "homoserine methionine biosynthesis" (rosetta data set). There are 1 possible assignments of genes to reactions.**

Number	Normalized Score	Number of Pairs	Positive Pairs	Negative Pairs	Zero Pairs	Assignments
1	3.09	3	2	1	0	2.7.2.11 : YDR300C<br>1.2.1.41 : YOR323C<br>1.5.1.2 : YER023W

Table 43: **Assignments for the pathway "proline biosynthesis I" (rosetta data set). There are 1 possible assignments of genes to reactions.**

Number	Normalized Score	Number of Pairs	Positive Pairs	Negative Pairs	Zero Pairs	Assignments
1	37.35	1	1	0	2	1.8.1.2 : 1944<br>2.7.1.25 : YKL001C<br>2.7.7.4 : YJR010W

Table 44: **Assignments for the pathway "sulfate assimilation 2" (rosetta data set). There are 1 possible assignments of genes to reactions.**

Number	Normalized Score	Number of Pairs	Positive Pairs	Negative Pairs	Zero Pairs	Assignments
1	-1.64	1	0	1	0	2.6.1.16 : YKL104C<br>2.7.7.23 : YDL103C

Table 45: **Assignments for the pathway "UDP-N-acetylglucosamine biosynthesis" (rosetta data set). There are 1 possible assignments of genes to reactions.**

Number	Normalized Score	Number of Pairs	Positive Pairs	Negative Pairs	Zero Pairs	Assignments
1	35.31	3	3	0	0	2.1.3.3 : YJL088W<br>4.3.2.1 : YHR018C<br>6.3.4.5 : YOL058W

Table 46: **Assignments for the pathway "arginine biosynthesis I" (rosetta data set). There are 1 possible assignments of genes to reactions.**

Number	Normalized Score	Number of Pairs	Positive Pairs	Negative Pairs	Zero Pairs	Assignments
1	24.88	12	12	0	24	2.4.2.17 : YER055C<br>3.5.4.19 : 601<br>3.6.1.31 : 601<br>5.3.1.16 : YIL020C<br>4.2.1.19 : YOR202W<br>2.6.1.9 : YIL116W<br>3.1.3.15 : 1939<br>1.1.1.23 : 601<br>1.1.1.23 : 601

Table 47: **Assignments for the pathway "histidine biosynthesis I" (rosetta data set). There are 1 possible assignments of genes to reactions.**

Number	Normalized Score	Number of Pairs	Positive Pairs	Negative Pairs	Zero Pairs	Assignments
1	13.90	3	3	0	0	2.6.1.13 : YLR438W<br>1.5.1.12 : YHR037W<br>3.5.3.1 : YPL111W

Table 48: **Assignments for the pathway "arginine degradation I" (rosetta data set). There are 1 possible assignments of genes to reactions.**

Number	Normalized Score	Number of Pairs	Positive Pairs	Negative Pairs	Zero Pairs	Assignments
1	6.68	3	1	2	0	1.1.1.3 : YJR139C<br>1.2.1.11 : YDR158W<br>2.7.2.4 : YER052C

Table 49: **Assignments for the pathway "homoserine biosynthesis" (rosetta data set). There are 1 possible assignments of genes to reactions.**

Number	Normalized Score	Number of Pairs	Positive Pairs	Negative Pairs	Zero Pairs	Assignments
1	13.43	3	3	0	0	3.5.3.1 : YPL111W<br>2.6.1.13 : YLR438W<br>1.5.1.2 : YER023W

Table 50: **Assignments for the pathway "arginine proline degradation" (rosetta data set). There are 1 possible assignments of genes to reactions.**

Number	Normalized Score	Number of Pairs	Positive Pairs	Negative Pairs	Zero Pairs	Assignments
1	1.98	1	1	0	0	6.3.2.3 : YOL049W<br>6.3.2.2 : YJL101C

Table 51: **Assignments for the pathway "glutathione biosynthesis" (rosetta data set). There are 1 possible assignments of genes to reactions.**

Number	Normalized Score	Number of Pairs	Positive Pairs	Negative Pairs	Zero Pairs	Assignments
1	2.86	1	1	0	0	1.13.11.11 : YJR078W<br>3.5.1.9 : YJL060W

Table 52: **Assignments for the pathway "tryptophan kynurenine degradation" (rosetta data set). There are 1 possible assignments of genes to reactions.**
